# Programmable Construction of Supramolecular Polymers Achieved in Neutral Lipid Environments

**DOI:** 10.1038/s41467-026-74683-9

**Published:** 2026-07-06

**Authors:** Miku Naruse, Natsumi Fukaya, Yoshiki Imai, Soichiro Ogi, Masayasu Taki, Shigehiro Yamaguchi

**Affiliations:** 1 Department of Chemistry, Graduate School of Science, Nagoya University, Furo, Chikusa, Nagoya, 464-8602 Japan; 2 Integrated Research Consortium on Chemical Science (IRCCS), Nagoya University, Furo, Chikusa, Nagoya, 464-8602 Japan; 3 Institute of Transformative Bio-Molecules (WPI-ITbM), Nagoya University, Furo, Chikusa, Nagoya, 464-8601 Japan; 4 Institute for Glyco-core Research (iGCORE), Gifu University, 1-1 Yanagido, Gifu, 501-1193 Japan

**Keywords:** Supramolecular polymers, Self-assembly, Polymers

## Abstract

Supramolecular polymerization within living cells has emerged as a promising strategy for controlling cellular functions. Lipid droplets, intracellular organelles mainly composed of neutral lipids, provide low-polarity environments, yet their role in regulating supramolecular assembly pathways remains unclear. Here we show that triolein, a representative triacylglycerol featuring three ester groups and cis-9-octadecenyl chains, acts as an effective medium for kinetically controlled supramolecular polymerization. An alanine-based diamide-functionalized fluorophore forms supramolecular polymers in triolein through a nucleation-elongation mechanism, in which an initial nucleus triggers subsequent growth. Although the thermodynamic stability is comparable to that in di-*n*-butyl ether, a commonly used organic solvent, triolein suppresses spontaneous nucleation and inter-fiber bundling, creating a lag phase during which seeded polymerization guides monomers along a defined assembly pathway. Kinetic studies with ethyl oleate, a structural analogue of triolein, indicate that transient solute-solvent interactions contribute to the suppression of both nucleation and bundling events. This kinetic control enables stepwise seeded growth of multiblock nanostructures and establishes neutral lipids as functional media for the precision-controlled construction of supramolecular polymers.

## Abstract (web summary)

Lipid droplets provide low-polarity environments, yet their role in regulating supramolecular assembly pathways remains unclear. Here the authors demonstrate that triolein suppresses spontaneous nucleation and inter-fiber bundling in nucleation–elongation supramolecular polymerization of diamide-functionalized fluorophores, enabling stepwise seeded growth of multiblock nanostructures.

## Introduction

Lipid droplets (LDs) are cellular organelles composed mainly of neutral lipids, such as triacylglycerols and cholesterol esters, and play essential roles in cellular functions through regulated lipid storage and interactions with other organelles^1–3^. Dysregulation of lipid storage and inter-organelle communication has been linked to metabolic and proliferative diseases, including obesity, diabetes, and cancer^4–8^. Consequently, increasing attention has been directed toward understanding LD behavior in cells, propelled by the development of LD-specific fluorescent probes^9–19^ and advanced fluorescence imaging techniques^20–22^. Despite this progress in visualization, molecular strategies that can operate in LD-relevant environments and actively manipulate LD-associated processes remain limited. In this context, supramolecular polymerization within living cells has developed over the past two decades as a promising strategy for controlling cellular functions^23–36^. This prompted us to explore whether neutral lipid media, which serve as simplified model environments relevant to LDs, might offer an opportunity not only to study supramolecular assembly under LD-related conditions but also to examine how these media regulate assembly pathways. Recently, we demonstrated that a self-assembling fluorescent probe, designed by introducing two phenylalanine-based diamide units into our previously reported super-photostable LD marker^14^, forms and grows into emissive supramolecular assemblies inside artificial LDs dispersed in water^37^. However, the influence of neutral-lipid media on the thermodynamics and kinetics of supramolecular polymerization remained unexplored.

**Fig. 1:**
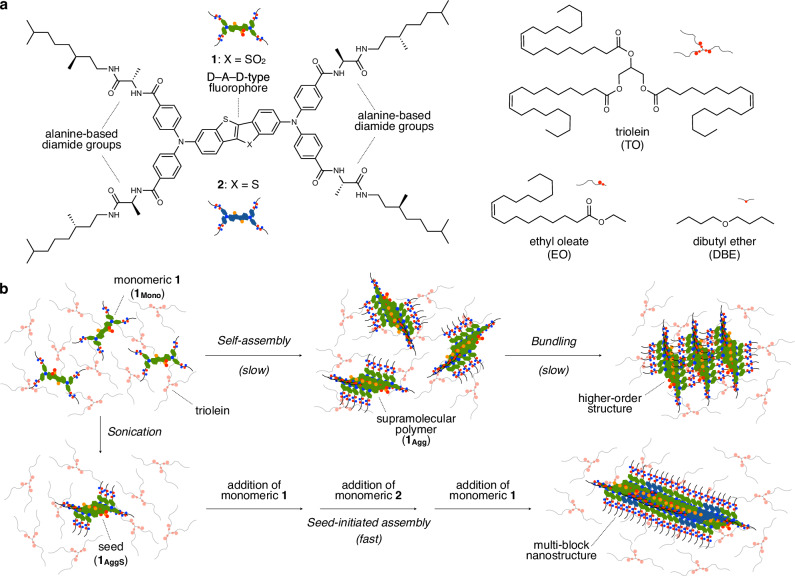
Molecular design and supramolecular assembly in triolein. **a** Chemical structures of diamide-functionalized fluorophores (**1,** **2**), triolein (TO), and reference solvents, ethyl oleate (EO) and di-*n*-butyl ether (DBE). **b** Schematic representation of the spontaneous self-assembly (top) and the seed-initiated growth of supramolecular block copolymers (bottom).

To harness the functional potential of low-polarity neutral lipids in supramolecular polymerization, we focused on their role in controlling nucleation, fiber growth, and inter-fiber bundling through solvent-solute interactions. Previous studies have demonstrated that solvent compatibility critically guides supramolecular building blocks into well-defined helical or hierarchical architectures under thermodynamic control^38–48^. Solvent composition can likewise exert a pronounced effect on kinetically controlled assembly pathways^49,50^. However, these insights have largely been established in conventional organic solvents, and it remains unclear whether neutral-lipid media can exert assembly-regulating effects beyond those expected from bulk polarity alone. Because the early stages of aggregation are often governed by subtle solvation differences and specific intermolecular interactions, bulk properties such as polarity or viscosity may not fully capture solvent behavior. On this basis, we identified triolein (TO), a representative triacylglycerol, as an LD-relevant model medium for testing this idea. Although TO has a dielectric constant (*ε* = 3.20 at *T* = 25 °C) similar to that of di-*n*-butyl ether (DBE; *ε* = 3.06 at *T* = 25 °C)^51^, its molecular structure is markedly distinct, featuring three ester groups and three *cis*−9-octadecenyl chains (Fig. 1a, right). This combination of hydrogen-bond-accepting carbonyl groups and a hydrocarbon-rich environment is expected to influence nucleation and bundling behavior in a manner not accessible in conventional low-polarity solvents.

To this end, we conducted a combined thermodynamic and kinetic investigation to uncover how TO enables programmable control over polymer growth and morphology. To this end, we designed a supramolecular building block, **1**, featuring a donor-acceptor-donor-type fluorophore core functionalized with alanine-based diamide groups (Fig. 1a, left)^52–57^. In our previous studies on amino acid-based diamide systems, we established a kinetic framework for controlling supramolecular polymerization pathways under metastable, non-equilibrium conditions^54^. Building on this background, we investigated whether a neutral lipid medium could actively reshape such kinetically controlled assembly pathways beyond what would be expected from bulk polarity alone. Spectroscopic and microscopic characterization revealed that **1** undergoes cooperative supramolecular polymerization in TO, yielding green-emissive fibrous assemblies with thermodynamic stability comparable to those formed in DBE. Further investigation using ethyl oleate (EO; *ε* = 3.17 at *T* = 28 °C), a reference solvent bearing a partial structure of TO, demonstrated that TO delays spontaneous nucleation and promotes dispersion of the fibrous assemblies, thereby preventing inter-fiber bundling into larger aggregated structures. Building on these solvent-dependent kinetic features, we successfully constructed fluorescent supramolecular multiblock copolymers by seeded co-assembly of **1** with a blue-emissive analog, **2** (Fig. 1b). These results demonstrate that neutral-lipid media are not simply passive low-polarity environments, but can contribute to the kinetically controlled construction of supramolecular nanostructures.

## Results

### Synthesis

The green-emitting compound **1** was synthesized in four steps starting from a 2,7-dibrominated precursor of [1]benzothieno[3,2-*b*][1]benzothiophene (BTBT) (Supplementary Figs. 1 and 2). Oxidation of one sulfur atom in the BTBT core using *m*-chloroperoxybenzoic acid, followed by Buchwald-Hartwig cross-coupling with *N*,*N*-bis(4-ethoxycarbonylphenyl)amine, afforded a D–A–D-type intermediate. Subsequent hydrolysis of the four ethyl ester groups under basic conditions, followed by amidation with (*S*)−2-amino-*N*-((*S*)−3,7-dimethyloctanyl)propaneamide, yielded the final compound. Compound **2**, the non-oxidized analogue of **1**, was synthesized in three steps from 2,7-diamino-substituted BTBT via Ullmann coupling with ethyl 4-iodobenzoate, followed by ester hydrolysis and final amidation (Supplementary Fig. 3). Detailed synthetic procedures and characterization data for the supramolecular building blocks are provided in the Supplementary Information.

### Self-assembly behavior of 1 in triolein

**Fig. 2:**
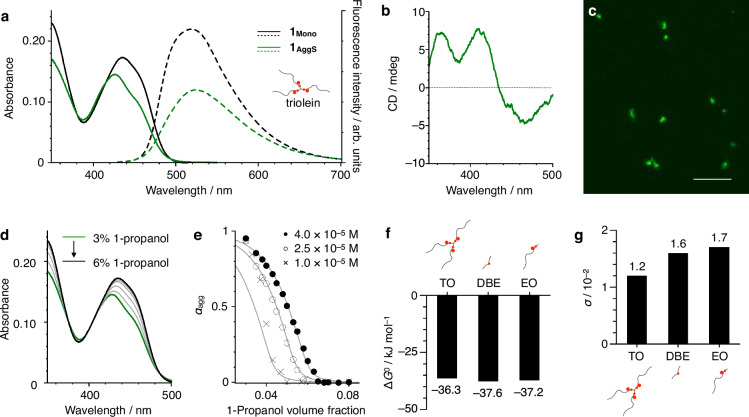
Thermodynamic studies of the self-assembly behavior of 1 in triolein. **a** UV-vis absorption (solid lines) and fluorescence (dashed lines) spectra of **1**_**Mono**_ (black) and **1**_**AggS**_ (green). **b** Circular dichroism (CD) spectrum of **1**_**AggS**_ in 97:3 TO/1-propanol at *c*_T_ = 1.0 × 10^–5^ M and *T* = 293 K. **c** CLSM image of **1**_**AggS**_; *λ*_ex_ = 405 nm; *λ*_em_ = 490–590 nm; scale bar: 5 μm. **d** Solvent-dependent UV–vis absorption spectra of **1** in binary mixtures of TO and 1-propanol at *c*_T_ = 1.0 × 10^–5^ M and *T* = 293 K. **e** Aggregation parameter (*α*_agg_) calculated from the raw absorbance data at *λ*_abs_ = 433 nm based on the global-fit result of the solvent-dependent cooperative model, plotted against the volume fraction of 1-propanol. Solid lines represent fitted curves. **f,g** Comparison of (**f**) Δ*G*^0^ and (**g**) *σ* values obtained from fitting the denaturation curves of **1**_**AggS**_ in TO (Fig. 2e), in DBE (Supplementary Fig. S6d), and in EO (Supplementary Fig. S7d), each containing 3 vol% of 1-propanol. TO, DBE, and EO denote triolein, di-*n*-butyl ether, and ethyl oleate, respectively. *c*_T_ denotes the total concentration of **1**. *λ*_ex_, *λ*_em_, and *λ*_abs_ denote the excitation, emission, and absorption wavelengths, respectively.

We first investigated the self-assembly behavior of **1** in TO using UV-vis absorption spectroscopy. For sample preparation, TO was added to a solution of **1** in 1-propanol until the final composition reached 97 vol%, with a total concentration (*c*_T_) of 1.0 × 10^–5^ M at a temperature (*T*) of 293 K. Under these mixing conditions, fluorescence microscopy of the TO/1-propanol mixture did not reveal any micrometer-scale droplet-like phase separation. The resulting solution exhibited a low-energy absorption with a maximum (*λ*_abs_) at 433 nm (Fig. 2a, black solid line). This wavelength was similar to that of molecularly dispersed **1** (**1**_**Mono**_) in 1-propanol (Supplementary Fig. 4a). However, the emission maximum (*λ*_em_) showed a hypsochromic shift from 545 nm in 1-propanol to 519 nm in TO (black dashed line in Fig. 2a). This *λ*_em_ value closely resembles that in toluene (Supplementary Fig. 4b), suggesting that the shift arises from the polarity-sensitive nature of D–A–D-type fluorophores. Upon subjecting the solution to sonication, a distinct hypsochromic shift in the absorption band to 420 nm was observed (green solid line in Fig. 2a). This spectral change was accompanied by the emergence of positive circular dichroism (CD) signals at 362 nm and 406 nm and a negative signal at 466 nm (Fig. 2b), indicating aggregate formation with chiral excitonic coupling between chromophores. Notably, the resulting aggregates retained moderate fluorescence (green dashed line in Fig. 2a), which enabled visualization of green-emissive nanostructures with a size of less than 1 µm in TO using fluorescence imaging (Fig. 2c). Hereafter, this sonication-generated aggregate state is referred to as **1**_**AggS**_.

To gain mechanistic insights into the self-assembly process, denaturation experiments were conducted by gradually adding a solution of **1**_**Mono**_ in 1-propanol to a solution of **1**_**AggS**_ in TO/1-propanol (97:3, *v*/*v*) at *T* = 293 K. Increasing the proportion of 1-propanol led to a bathochromic shift of the absorption maximum from 420 nm to 433 nm, with a clear isosbestic point at 375 nm, indicating a transition from **1**_**AggS**_ to **1**_**Mono**_ (Fig. 2d and Supplementary Fig. 5). This transition followed a non-sigmoidal profile, which was well fitted using a cooperative denaturation model with global fitting (Fig. 2e)^58^. The fitting yielded a standard Gibbs free energy (Δ*G*^0^) of –36.3 kJ·mol^–1^ and a cooperative parameter (*σ*) of 1.2 × 10^–2^ (Supplementary Table 1).

To assess the thermodynamic stability of **1**_**AggS**_ in TO, analogous assemblies were prepared in DBE and EO. The UV-vis and CD spectra of these samples were comparable to those of **1**_**AggS**_ in 97:3 TO/1-propanol (Supplementary Fig. 6). Transmission electron microscopy (TEM) confirmed that **1**_**AggS**_ in DBE adopted one-dimensional fibrous morphologies (Supplementary Fig. 7). Fourier transform infrared (FT-IR) spectroscopy revealed a shift in the N–H stretching band from 3436 cm^–1^ and 3318 cm^–1^ in chloroform to 3287 cm^–1^ in the dried aggregates obtained from a solution of **1** in a 97:3 mixture of DBE and 1-propanol, indicating intermolecular hydrogen bonding between amide moieties within the aggregates (Supplementary Fig. 8). Solvent-dependent denaturation experiments were also conducted using 97:3 mixtures of DBE/1-propanol and EO/1-propanol (Supplementary Figs. 9 and 10). These experiments yielded Δ*G*^0^ values of –37.6 kJ·mol^–1^ and –37.2 kJ·mol^–1^, respectively, with *σ* values comparable to that obtained in TO (Supplementary Table 1). These results indicate no substantial variation in the thermodynamic stability of the supramolecular assemblies formed in these three low-polarity environments, despite their distinct molecular structures.

### Effects of triolein on assembly kinetic

**Fig. 3:**
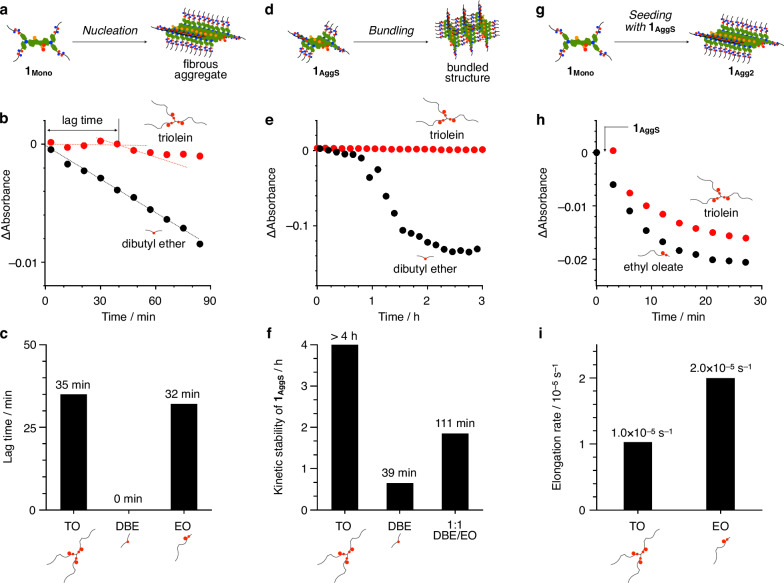
Kinetic studies of the self-assembly behavior of 1 in triolein and related solvents. **a, d, g** General schematic illustrations of the processes examined in this figure: spontaneous nucleation of **1**_**Mono**_ (**a**), bundling of **1**_**AggS**_ (**d**), and seed-initiated assembly of **1** (**g**). **b, e, h** Time-dependent changes in absorbance at 433 nm for **1**_**Mono**_ (**b**) and **1**_**AggS**_ (**e**) in 97:3 TO/1-propanol (*c*_T_ = 1.0 × 10^–5^ M; red filled circles) and in 97:3 DBE/1-propanol (*c*_T_ = 1.0 × 10^–5^ M; black filled circles), and before and after addition of a solution of **1**_**AggS**_ (*c*_T_ = 1.0 × 10^–5^ M; 0.12 mL) to a fresh solution of **1**_**Mono**_ (*c*_T_ = 1.0 × 10^–5^ M; 1.2 mL) in 97:3 TO/1-propanol (red) and in EO/1-propanol (black) at *T* = 293 K (**h**). **c, f, i** Comparison of the lag time before nucleation (**c**), the kinetic stability of **1**_**AggS**_ (**f**), and the elongation rate determined at *λ*_abs_ = 433 nm in each solvent containing 3 vol% of 1-propanol (**i**).

We next examined the effect of TO on the early stage of self-assembly of **1**_**Mono**_ (Fig. 3a). A freshly prepared solution of **1** in TO/1-propanol (97:3, *v*/*v*; 1.0 × 10^–5^ M) displayed a UV–vis absorption spectrum at 433 nm, characteristic of the monomeric state (Supplementary Fig. 11a), with negligible spectral change observed over 30 min (red filled circles in Fig. 3b). This indicates the presence of a pronounced lag phase prior to nucleation. In contrast, supramolecular polymerization of **1** in DBE proceeded rapidly upon sample preparation (Supplementary Fig. 11b), as evidenced by immediate spectral changes and the absence of any detectable lag time (black filled circles in Fig. 3b). To examine whether this kinetic difference can be explained by a simple bulk solvent property, we investigated EO, a solvent bearing a long alkyl chain and a carbonyl group. In this solvent, we again observed a measurable lag phase (~30 min) before the onset of self-assembly, similar to that seen in TO (Fig. 3c; Supplementary Fig. 11c, d). This behavior is inconsistent with a simple viscosity-based explanation, because TO is considerably more viscous (44.7 mm^2^·s^–1^) than EO (7.01 mm^2^·s^–1^). To further clarify the origin of this delay, we performed additional experiments using mixed solvents. In DBE/EO mixtures, the lag time changed nonlinearly and increased sharply in EO-rich regimes (Supplementary Fig. 12). Moreover, in EO/TO mixtures, a lag time comparable to that in pure TO was retained even when TO was diluted with 50% EO (Supplementary Fig. 13). These nonlinear composition dependences are difficult to reconcile with viscosity alone and instead point to a major contribution from specific solvent-monomer interactions. Indeed, FT-IR spectroscopy revealed that the carbonyl group of EO engages in hydrogen bonding with the diamide moiety of **1** (Supplementary Fig. 14). Such interactions likely stabilize the monomeric state and retard nucleation. These results suggest that specific solvent–monomer interactions, particularly hydrogen bonding between the carbonyl groups of the neutral lipids and the diamide moiety of **1**, play a more dominant role than viscosity in determining the kinetics of self-assembly.

We then turned to the kinetic stability of the sonication-generated aggregate **1**_**AggS**_ before its evolution into bundled structures, as evaluated by time-dependent UV–vis absorption spectroscopy and fluorescence imaging (Fig. 3d). In TO, **1**_**AggS**_ retained its spectral features and morphology for several hours at 293 K (red closed circles in Fig. 3e; Supplementary Fig. 15a). By contrast, in DBE, the absorption spectrum of **1**_**AggS**_ remained largely unchanged for several tens of minutes but subsequently exhibited a gradual decline in absorbance over time (black filled circles in Fig. 3e; Supplementary Fig. 15b). Notably, the kinetic stability of **1**_**AggS**_ was enhanced upon the addition of 50 vol% EO to DBE (Fig. 3f; Supplementary Fig. 15c, d). A similarly high kinetic stability was also observed in pure EO, indicating that this stabilizing effect persists in EO-rich media (Supplementary Fig. 16). In addition, in EO/TO mixtures, a lag time longer than 4 h was retained even when only 25 vol% TO was added to EO (Supplementary Fig. 16). These results suggest the presence of solvent-specific interactions that strongly influence the kinetic stability of **1**_**AggS**_. This behavior is likely related to the cis-9-octadecenyl-chain-rich hydrocarbon environment in TO and EO, which suppresses inter-fiber association and thereby inhibits the formation of bundled structures. These observations highlight TO’s potential as a medium that not only preserves nanostructural integrity but also promotes homogeneous dispersion of supramolecular assemblies, thereby preventing undesired bundling.

### Seed-initiated assembly in triolein

Using the aggregates (**1**_**AggS**_ shown in Fig. 2c) and the kinetically trapped monomers identified in the previous experiment (Fig. 3a), we further investigated the kinetics of the elongation process by mixing these solutions in TO/1-propanol (97:3, *v*/*v*) at a **1**_**Mono**_/**1**_**AggS**_ volume ratio of 10:1 (Fig. 3g) The UV–vis absorption spectrum of the resulting mixture exhibited an immediate blue shift to 420 nm and a concurrent decrease in absorbance at 433 nm, indicating the onset of polymer growth without any lag phase (red filled circles in Fig. 3h). In contrast, no spectral changes were observed in the monomer-only solution for up to 35 min (red filled circles in Fig. 3b), confirming that the added **1**_**AggS**_ served as effective seeds to initiate supramolecular polymerization. To assess the effect of the low-polarity solvent on the elongation rate, we repeated the seeding experiment using EO. Compared to TO, EO facilitated a faster elongation process, increasing the rate from –d*Abs*/d*t* = 1.0 × 10^–5^ s^–1^ in TO to 2.0 × 10^–5^ s^–1^ in EO (Fig. 3i; Supplementary Fig. 17). We further validated the feasibility of a sequential seeded growth process by introducing the elongated aggregates with a length of around 3 µm, hereafter denoted as **1**_**AggL**_ to distinguish them from the sonication-prepared **1**_**AggS**_ (Supplementary Fig. 18c, d), into a fresh solution of **1**_**Mono**_ in TO/1-propanol (97:3, *v*/*v*) at a 30:1 volume ratio of **1**_**Mono**_ to **1**_**AggL**_. A clear transition from monomer to aggregate was observed (Supplementary Fig. 18a, b). CLSM imaging confirmed the formation of supramolecular polymers extending over 15 μm in length (Supplementary Fig. 18e), underscoring the robustness of this seeding method in low-polarity neutral lipid environments.

**Fig. 4:**
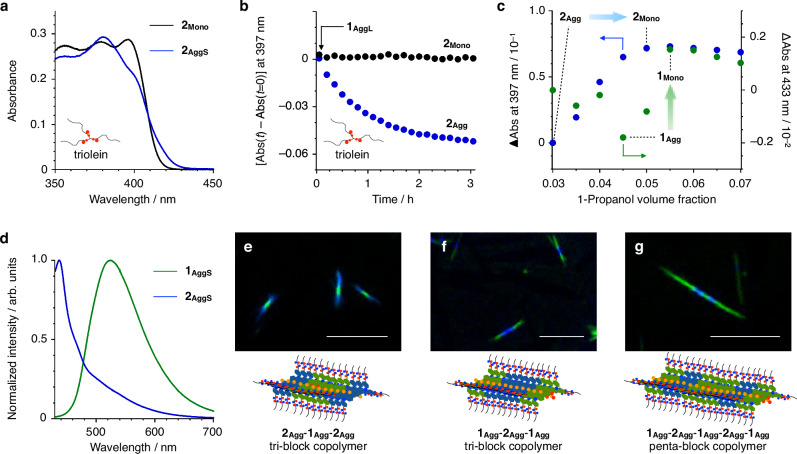
Seed-initiated supramolecular polymerization of 1 and 2 in triolein. **a** UV–vis absorption spectra of **2** in 97:3 TO/1-propanol at *c*_T_ = 1.0 × 10^–5^ M and *T* = 293 K before (black line) and after sonication (blue line). **b** Time-dependent changes in absorbance at *λ*_abs_ = 397 nm for **2**_**Mono**_ (1.0 × 10^–5^ M; 1.2 mL) upon seeding with **1**_**AggL**_ (0.04 mL) in 97:3 TO/1-propanol at *T* = 293 K. **c** Solvent-dependent changes in the absorbance (ΔAbs) at *λ*_abs_ = 397 nm and *λ*_abs_ = 433 nm upon addition of 1-propanol to the solution of copolymers of **1** and **2**. **d** Fluorescence spectra of **1**_**AggS**_ and **2**_**AggS**_ in 97:3 TO/1-propanol. **e**–**g** CLSM images of supramolecular (**e**, **f**) triblock and (**g**) pentablock copolymers of **1** and **2**; *λ*_ex_ = 405 nm; blue channel: *λ*_em_ = 420–460 nm; green channel: *λ*_em_ = 580–610 nm; scale bars: 5 µm.

The successful demonstration of seed-initiated polymerization of **1** in TO points to potential for further kinetic control over supramolecular copolymer nanostructures derived from binary mixtures. To explore this possibility, we turned our attention to a blue-emissive analogue, **2**, and examined its self-assembly behavior in TO/1-propanol (97:3, *v*/*v*; 1.0 × 10^–5^ M). As with **1**, the UV–vis absorption spectrum of molecularly dispersed **2** (**2**_**Mono**_) was first observed, which transitioned to an aggregated state upon sonication (Fig. 4a). This was accompanied by the emergence of a bisignate CD signal (Supplementary Fig. 19a, b). The resulting aggregate solution (**2**_**AggS**_) exhibited blue fluorescence at 437 nm (Supplementary Fig. 19c), and its supramolecular structures were confirmed by CLSM imaging (Supplementary Fig. 19d). Denaturation experiments revealed that the disassembly of **2**_**AggS**_ to **2**_**Mono**_ followed a cooperative nucleation–elongation mechanism (Supplementary Fig. 20), making **2** amenable to seeded growth (Supplementary Fig. 21).

Motivated by the lag time observed prior to spontaneous nucleation of **2**, we examined whether **2**_**Mono**_ could preferentially assemble onto externally added **1**_**AggL**_ seeds. A solution of **1**_**AggL**_ was prepared in advance by mixing **1**_**Mono**_ and **1**_**AggS**_ at a volume ratio of 10:1, followed by incubation at 293 K for 60 min. Subsequently, time-dependent UV–vis absorption spectra of a freshly prepared solution of **2**_**Mono**_ were recorded at 293 K. While the spectrum of **2**_**Mono**_ remained unchanged in the absence of **1**_**AggL**_ (black filled circles in Fig. 4b), the addition of **1**_**AggL**_ led to a rapid decrease in absorbance at 397 nm (blue closed circles in Fig. 4b; Supplementary Fig. 22a). The resulting CD spectrum closely resembled that of aggregated **2** (Supplementary Fig. 22b). These results indicate that supramolecular polymerization of **2**_**Mono**_ was initiated by the presence of aggregated **1**.

To gain insight into the resulting copolymer structure, absorbance changes at 397 nm and 433 nm were monitored during the gradual addition of 1-propanol to the solution of copolymers. Disassembly of the **2**_**Agg**_ segment into monomers was observed as the 1-propanol content increased from 3 vol% to 4.5 vol%, whereas an increase in **1**_**Mono**_ absorbance was detected only at 4.5–5.5 vol% (Fig. 4c; Supplementary Fig. 23). Compared to the denaturation behavior of **1** homopolymers (Fig. 2e), the domain of the aggregated **1** in the copolymer appeared more persistent upon addition of 1-propanol. A related kinetic effect has been reported for block supramolecular polymers formed in low-polarity media through intermolecular hydrogen-bonding interactions, in which disassembly proceeds preferentially from the polymer termini, leading to enhanced apparent persistence of the central block^59^. In the present system, we therefore interpret this behavior in terms of structural and kinetic effects.

### Fluorescence imaging of multiblock copolymers

Finally, leveraging the distinct emission wavelengths of **1** and **2** in their aggregated states (Fig. 4d), CLSM imaging was employed to visualize the structural features of supramolecular copolymers prepared via seeded growth. When aggregated **1** was used as a seed for the growth of **2** (Supplementary Fig. 24a, b), the resulting copolymer exhibited clearly distinguishable terminal and central domains, emitting blue (*λ*_ex_ = 405 nm; *λ*_em_ = 420–460 nm) and green (*λ*_ex_ = 405 nm; *λ*_em_ = 580–610 nm) fluorescence, respectively (Supplementary Fig. 24c, d). The merged CLSM image confirmed that aggregated **2** domains grew from both ends of aggregated **1**, resulting in the formation of a triblock copolymer, **2**_**Agg**_-**1**_**Agg**_-**2**_**Agg**_ (Fig. 4e). Additionally, elongation of **1** from aggregated **2** was also successfully achieved, yielding green-blue-green nanofibers as observed by CLSM (Fig. 4f; Supplementary Fig. 25). Furthermore, **1**_**Mono**_ was added to the triblock **2**_**Agg**_-**1**_**Agg**_-**2**_**Agg**_, resulting in a well-defined pentablock copolymer. Time-dependent UV–vis absorption measurements confirmed the gradual consumption of **1**_**Mono**_ (Supplementary Fig. 26), and CLSM imaging revealed supramolecular nanofibers with alternating green-blue-green-blue-green emission patterns, characteristic of pentablock architectures (Fig. 4g). These results underscore the utility of TO as a medium whose kinetic influence on supramolecular polymerization enables sequential seeded growth in a controlled manner, leading to pathway-selective construction of multiblock supramolecular polymer architectures.

## Discussion

To harness the structural features of neutral lipid environments for controlled supramolecular polymerization, we integrated thermodynamic and kinetic analysis across three solvents (TO, EO, and DBE) while keeping 1-propanol fixed as the good solvent. Our findings demonstrate that TO functions as an effective solvent for promoting the cooperative nucleation–elongation assembly of diamide-functionalized fluorophores into nanofibers, in a manner comparable to DBE, a conventional low-polarity organic solvent with a similar dielectric constant. A key distinction, however, emerged in the *m*-values, which quantify how the Gibbs free energy of supramolecular polymerization depends on the fraction of a denaturing solvent (1-propanol in this case) in a mixed solvent system^58^. Specifically, our denaturation analysis revealed significantly higher *m*-values in TO (202 kJ·mol^–1^) than in DBE (123 kJ·mol^–1^) (Supplementary Table 1), indicating that TO offers an enhanced dynamic range for modulating the polymer-monomer equilibria. This pronounced solvent responsiveness does not arise from differences in intrinsic thermodynamic stability or cooperativity, as both Δ*G*^0^ and *σ* values were comparable. Rather, the elevated *m*-value reflects differences in aggregate morphology. In DBE, supramolecular polymers tend to evolve into more bundled aggregates upon standing, as suggested by the absorption changes (black filled circles in Fig. 3e; Supplementary Fig. 15b) and by TEM observations showing a more bundled morphology after standing of **1**_**AggS**_ for 120 min (Supplementary Fig. 27a, b). Such bundling likely shields the hydrogen-bonding diamide moieties and thereby dampens the response to 1-propanol. In contrast, TO facilitates the formation of well-dispersed supramolecular polymers, as supported by CLSM observations showing similarly fragmented fibrous aggregates even after 4 h (Fig. 2 and Supplementary Fig. 27c, d). This dispersed morphology, likely stabilized by van der Waals interactions with the three cis-9-octadecenyl chains, exposes the diamide groups to 1-propanol and enables more efficient depolymerization.

These observations align with the reported theoretical insights suggesting that good solvents do not depolymerize supramolecular assemblies by destabilizing the polymer, but rather by stabilizing monomers through favorable solvation^38,60,61^. The *m*-value, in this context, emerges as a useful descriptor for evaluating how effectively monomeric states are stabilized in different environments. In TO, the combination of a dispersed polymer morphology and weak interactions with 1-propanol enables efficient solvation of monomers, thereby enhancing depolymerization and resulting in a correspondingly higher *m*-value. Among the three solvents, EO exhibited the highest *m*-value (230 kJ·mol^–1^), likely due to two synergistic factors: its lower viscosity, which accelerates solute–solvent exchange, and its higher miscibility with 1-propanol^62^, which ensures more uniform solvation. In addition, FT-IR spectroscopy confirmed that EO can form hydrogen bonds with the monomer (Supplementary Fig. 14), further stabilizing the monomeric state. These results underscore the utility of the *m*-value as a sensitive descriptor for capturing both monomer stabilization and morphological features (bundling vs. dispersion) in the aggregated state.

We propose that TO suppresses spontaneous nucleation by stabilizing the monomeric state through hydrogen bonding between its ester carbonyl groups and the diamide moieties of **1**, while its cis-9-octadecenyl-chain-rich hydrocarbon environment stabilizes the dispersed supramolecular polymer state by suppressing van der Waals-driven inter-fiber bundling. Consistent with this proposal, our kinetic studies revealed delayed nucleation, stabilization of the dispersed aggregated state, and exceptional responsiveness to seeded growth (red filled circles in Fig. 3h), demonstrating that these solvent-specific effects enable precise kinetic control over supramolecular polymer growth. The observation of related kinetic features in EO further supports the view that neutral lipids can influence the kinetic pathway of supramolecular polymerization in ways not captured by conventional low-polarity solvents alone. Although the present FT-IR data support the participation of such solvent–monomer hydrogen bonding, the detailed organization and dynamic nature of these interactions remain to be clarified. Molecular dynamics simulations will be valuable for elucidating the preferred hydrogen-bonding motifs, their dynamics, and their roles in nucleation and bundling^63,64^. Importantly, we applied these solvent-dependent characteristics to achieve stepwise seeded growth of supramolecular multiblock nanostructures in TO. These findings identify low-polarity neutral lipids as useful media for directing pathway-selective supramolecular polymerization. Given that TO is a representative triacylglycerol present in LDs, these results also provide a basis for future studies aimed at designing self-assembling molecular systems that operate in LD-relevant environments.

## Methods

### Characterization

^1^H and ^13^C NMR spectra were recorded using a JEOL AL-400 spectrometer (400 MHz for ^1^H, 100 MHz for ^13^C) or a JEOL JNM-ECS400 (400 MHz for ^1^H, 100 MHz for ^13^C) in CDCl_3_, acetone-*d*_6_, CD_2_Cl_2_, or DMSO-*d*_6_. Chemical shifts are reported in *δ* ppm relative to the residual solvent peaks or the solvent signals as internal standards (^1^H NMR: CHCl_3_ *δ* 7.26, acetone *δ* 2.05, CH_2_Cl_2_ *δ* 5.32, and DMSO *δ* 2.50; ^13^C NMR: CDCl_3_ *δ* 77.16, acetone-*d*_6_ *δ* 29.84, and DMSO-*d*_6_ *δ* 39.52). Melting points (Mp) were determined using a Yanaco MP-S3 melting point apparatus. Mass spectra were acquired using a Thermo Fisher Scientific Exactive Plus Orbitrap MS System via electrospray ionization (ESI) or a Bruker Daltonics ultrafleXtreme MALDI-TOF/TOF mass spectrometer. The kinematic viscosities of triolein and ethyl oleate were measured at ambient temperature using an Ubbelohde-type viscometer (TN-102-05 and TN-102-07, Takao Manufacturing Co., Ltd.) and are reported in units of mm^2^ s^–1^.

### Synthesis

All reactions were carried out using dried glassware and under a nitrogen atmosphere unless otherwise noted. Thin-layer chromatography (TLC) was performed on silica gel 60F254-coated glass plates (0.25 mm thickness, Merck). Column chromatography was conducted using PSQ100B silica gel (Fuji Silysia Chemicals). Preparative gel permeation chromatography (GPC) was performed on a Japan Analytical Industry LC-918 instrument equipped with JAIGEL-2.5H and −3H columns, using CHCl_3_ as the eluent. All reagents were purchased from commercial suppliers and used without further purification unless otherwise stated. Anhydrous DMF was obtained from Kanto Chemicals and further purified using the Glass Contour solvent system. Full synthetic details and characterization data for the new compounds are provided in the Supplementary Information.

### Spectroscopy

Spectroscopic measurements were carried out under ambient conditions. 1-Propanol, toluene, di-*n*-butyl ether (DBE), and chloroform were purchased from NACALAI TESQUE, INC. and used without further purification. Triolein (TO) was purchased from Tokyo Chemical Industry (TCI) and further purified by silica gel column chromatography with toluene as the eluent. Ethyl oleate (EO) was obtained from FUJIFILM Wako Pure Chemical Corporation and used as received. Fourier transform infrared (FT-IR) spectra were recorded using a JASCO FT/IR-4200 spectrometer at ambient temperature. UV–vis absorption spectra were collected with a JASCO V-750 spectrophotometer using quartz cuvettes of 5 mm path length and a JASCO ETCR-762 cell holder for temperature control. Fluorescence spectra were measured using quartz cuvettes with a 5 mm path length and a JASCO FP-8500 spectrometer. Circular dichroism (CD) spectra were measured with a JASCO J-1500 spectrophotometer.

### Microscopy

Transmission electron microscopy (TEM) was performed using a JEOL JEM-1400EM operating at 80 kV. Samples (10 µL) were drop-cast onto carbon-coated copper grids (400 mesh), blotted with filter paper to remove excess solution, and dried under reduced pressure without additional staining. Supramolecular polymers formed in TO were visualized using confocal microscopy under the specified conditions. A Leica TCS SP8 STED 3X system equipped with an inverted DMI6000 CS microscope and a tunable pulsed white light laser (470–670 nm, 78 MHz repetition rate) was employed. For high-magnification imaging, an HC PL APO CS2 100×/1.40 oil-immersion objective was used.

### Sample preparation

Compound **1** or **2** was first dissolved in 1-propanol at a concentration of 3.3 × 10^–4^ M, followed by the addition of a selected solvent (toluene, TO, EO, or DBE) until the final solvent composition reached 97 vol%, yielding a final concentration of 1.0 × 10^–5^ M. These solutions were used to monitor the spontaneous assembly process from monomers to aggregates. In 97 vol% of DBE, visible precipitates formed after 4.5 h of standing. These precipitates were drop-cast onto CaF_2_ substrates and dried under reduced pressure for FT-IR measurements of **1** in the solid state. For FT-IR measurements, chloroform was used as a good solvent in place of 1-propanol. For denaturation experiments and kinetic stability assessment of the aggregated state (**1**_**AggS**_ or **2**_**AggS**_), freshly prepared solutions by combining the monomer solution in 1-propanol with 97 vol% of the respective solvent (TO, EO, or DBE) were transferred to a water bath and treated by ultrasonication at 31 kHz for a defined duration: 50 min for TO, 40 min for EO, and 20 min for DBE or 1:1 EO/DBE. The resulting **1**_**AggS**_ or **2**_**AggS**_ solutions were also employed in seed-initiated supramolecular polymerization experiments.

### Denaturation curve fitting

Solvent-dependent denaturation curves were analyzed by global fitting with a cooperative supramolecular polymerization equilibrium model based on the nucleation–elongation mechanism, following the approach of Korevaar et al.^54^. The experimental absorbance data were imported into the MATLAB fitting routine as raw data. Normalization was included during the fitting procedure by selecting the option “Normalize each curve individually”. The fitted curves shown in Fig. 2e, Figs. S6d, and S7d were used to derive the thermodynamic parameters summarized in Supplementary Table S1.

## Supplementary information

Supplementary Information

Transparent Peer Review file

## Source data

Source Data

## Data Availability

All data generated in this study including additional UV−vis, photoluminescence, CD, and FT-IR spectra, TEM and CLSM images, thermodynamic analyses are provided in the Supplementary Information (Supplementary Figs. 1–42 and Supplementary Table 1) and Source Data file. Additional data are available from the corresponding author upon request. A previous version of this manuscript has been deposited on a pre- print server^65^. Source data are provided with this paper.
