# Supplementary material for: Programmable Construction of Supramolecular Polymers Achieved in Neutral Lipid Environments
Source: Nat Commun. 2026 Jul 6;17:5732. doi: 10.1038/s41467-026-74683-9 (PMC13338038; doi:10.1038/s41467-026-74683-9)
Supplement: Supplementary file 1 — Supplementary Information [file 41467_2026_74683_MOESM1_ESM.pdf]

Supplementary Information

Programmable Construction of Supramolecular Polymers Achieved in Neutral Lipid Environments

Miku Naruse,[1] Natsumi Fukaya,[1] Yoshiki Imai,[1] Soichiro Ogi,*[,2] Masayasu Taki,[3,4] and Shigehiro Yamaguchi*[,1,2,3]

[1] Department of Chemistry, Graduate School of Science, Nagoya University, Furo, Chikusa, Nagoya 464-8602, Japan.

[2] Integrated Research Consortium on Chemical Science (IRCCS), Nagoya University, Furo, Chikusa, Nagoya 464-8602, Japan.

[3] Institute of Transformative Bio-Molecules (WPI-ITbM), Nagoya University, Furo, Chikusa, Nagoya 464-8601, Japan.

[4] Institute for Glyco-core Research (iGCORE), Gifu University, 1-1 Yanagido, Gifu 501-1193, Japan.

*Correspondence to: ogi.soichiro@chem.nagoya-u.ac.jp (S.O.), yamaguchi@chem.nagoya-u.ac.jp (S.Y.)

Table of Contents

Synthesis and Characterization S2

Supplementary Figures S6

NMR spectroscopy S22

References S30

Synthesis and Characterization

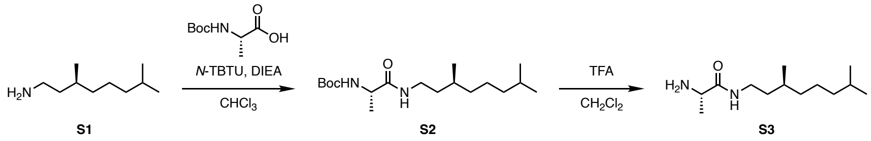

Supplementary Fig. 1. Synthesis of **S3**.

Compound S2. (3*S*)-3,7-Dimethyloctanamine (**S1**) was synthesized according to previously reported procedures.[1] To a solution of **S1** (1.38 g, 8.80 mmol), *N*-(*tert*-butoxycarbonyl)-L-alanine (1.51 g, 7.96 mmol), and *O*-(benzotriazol-1-yl)-*N*,*N*,*N'*,*N'*-tetramethyluronium tetrafluoroborate (*N*-TBTU, 7.17 g, 22.3 mmol) in $CHCl_3$ (150 mL) was added *N*,*N*-diethylethylamine (DIEA, 2.60 mL, 30.0 mmol), and the reaction mixture was stirred at room temperature for 42.5 h. The resulting mixture was filtrated through a pad of Celite®, and the filtrate was acidified with a 1 M aqueous solution of HCl. The aqueous layer was extracted with $CHCl_3$, and the combined organic layer was washed with brine, and dried over Na_2SO_4. After filtration, the solvent was removed under reduced pressure. The crude product was purified by silica gel column chromatography using a $CHCl_3$/ethanol solvent mixture as an eluent, followed by preparative GPC ($CHCl_3$) to afford **S2** as a yellow oily matter (2.06 g, 6.27 mmol, 79%). ^{1}H NMR (400 MHz, acetone-d_6): δ 7.19 (br, 1H), 6.05 (br, 1H), 4.08 (br, 1H), 3.30–3.18 (m, 2H), 1.60–1.46 (m, 2H), 1.40 (s, 9H), 1.36–1.06 (m, 11H), 0.91–0.84 (m, 9H); ^{13}C NMR (100 MHz, acetone-d_6): δ 173.1, 156.0, 79.1, 50.9, 40.0, 37.9, 37.7, 37.5, 31.0, 28.6, 28.5, 25.4, 23.0, 22.9, 19.8, 19.1; HRMS (APCI): *m*/*z* calcd. for $C_{18}H_{36}N_2O_3$: 329.2799 ($[M+H]^+$); found: 329.2811.

Compound S3. To a solution of **S2** (2.06 g, 6.27 mmol) in CH_2Cl_2 (50 mL) was added trifluoroacetic acid (TFA, 10 mL), and the mixture was stirred at room temperature for 12.5 h. After quenching with an aqueous solution of $NaHCO_3$, the mixture was extracted with $CHCl_3$. The combined organic layer was washed with brine, and dried over Na_2SO_4. After filtration, the solvent was removed under reduced pressure to afford **S3** as a colorless oil, which was used in the next step without further purification.

^{1}H NMR (400 MHz, $CDCl_3$): δ 3.30 (br, 1H), 3.17–2.97 (m, 2H), 1.55–0.88 (m, 13H), 0.85–0.63 (m, 9H); ^{13}C NMR (100 MHz, $CDCl_3$): δ 175.4, 77.4, 50.3, 38.8, 36.7, 36.3, 30.3, 27.5, 24.2, 22.3, 22.2, 21.4, 19.1; HRMS (APCI): *m*/*z* calcd. for $C_{13}H_{28}N_2O$: 229.2274 ($[M+H]^+$); found: 229.2281.

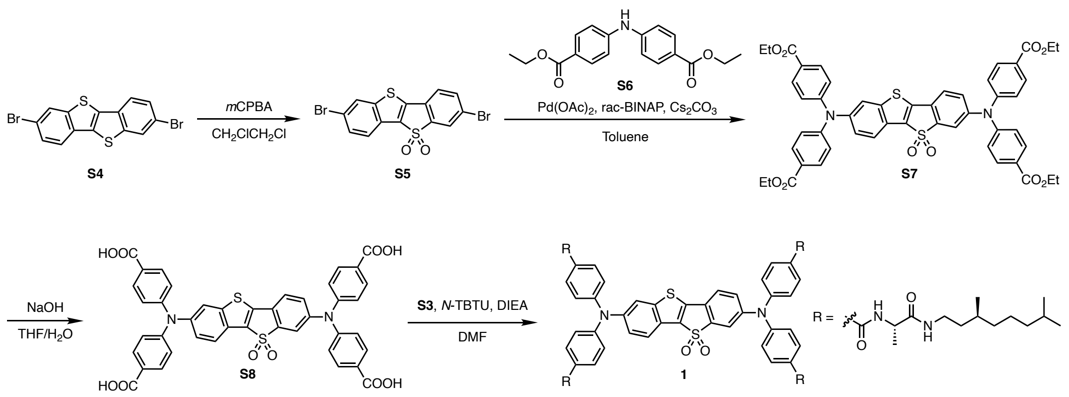

Supplementary Fig. 2. Synthesis of **1**.

Compound S5. 2,7-Dibromo[1]benzothieno[3,2-*b*][1]benzothiophene (**S4**) was synthesized according to previously reported procedures.[2–4] A solution of **S4** (981 mg, 2.46 mmol) in 1,2-dichloroethane (500 mL) was cooled to 0 °C, and *m*-chloroperoxybenzoic acid (*m*CPBA) (4.78 g, 27.7 mmol) was added dropwise. The reaction mixture was then warmed to room temperature and stirred for 5 d. After quenching with an aqueous solution of Na_2SO_3, the mixture was extracted with $CHCl_3$. The combined organic layer was washed with brine, and dried over Na_2SO_4. After filtration, the solvent was removed under reduced pressure. The resulting residue was washed with $CHCl_3$ to afford **S5** as a yellow solid (297 mg, 0.691 mmol, 28%). ^{1}H NMR (400 MHz, $CDCl_3$): δ 8.06 (d, J = 2.0 Hz, 1H), 7.91 (d, J = 1.6 Hz, 1H), 7.89 (d, J = 8.8 Hz, 1H), 7.75 (dd, J = 8.2, 1.8 Hz, 1H), 7.66 (dd, J = 8.6, 1.8 Hz, 1H), 7.39 (d, J = 8.4 Hz, 1H); HRMS (APCI): *m*/*z* calcd. for $C_{14}H_6Br_2O_2S_2$: 428.8249 ([M+H]$^+$); found: 428.8264.

Compound S7. Diethyl 4,4′-azanediyldibenzoate (**S6**) was synthesized according to previously reported procedures.[5] A mixture of **S6** (444 mg, 1.42 mmol), **S5** (207 mg, 0.481 mmol), $Pd(OAc)_2$ (21.7 mg, 0.0967 mmol), (±)-2,2′-bis(diphenylphosphino)-1,1′-binaphthyl (*rac*-BINAP) (41.4 mg, 0.0665 mmol), and Cs_2CO_3 (1.09 g, 3.35 mmol) was dissolved in anhydrous toluene (30 mL) and stirred at 120 °C for 10 days. After filtration through a pad of Celite®, the solvent was removed under reduced pressure. The crude product was purified by silica gel column chromatography using $CHCl_3$ as an eluent (R_f = 0.16), followed by preparative GPC ($CHCl_3$ as eluent), to afford **S7** as a yellow solid (135 mg, 0.151 mmol, 31%).
Mp: 139.5–140.1 °C; ^{1}H NMR (400 MHz, CD_2Cl_2): δ 8.00 (d, J = 9.2 Hz, 4H), 7.95 (d, J = 8.4 Hz, 4H), 7.88 (d, J = 8.8 Hz, 1H), 7.63 (d, J = 2.0 Hz, 1H), 7.48 (d, J = 1.6 Hz, 1H), 7.41 (d, J = 8.4 Hz, 1H), 7.35–7.29 (m, 2H), 7.20 (d, J = 8.8 Hz, 4H), 7.15 (d, J = 8.8 Hz, 4H), 4.39–4.29 (m, 8H), 1.41–1.33 (m, 12H); ^{13}C NMR (100 MHz, $CDCl_3$): δ 166.1, 165.9, 150.6, 149.8, 148.7, 145.0, 144.2, 143.0, 133.1, 131.6, 131.3, 127.9, 127.4, 126.8, 125.6, 125.4, 124.2, 123.3, 123.0, 122.9, 120.2, 117.4, 61.2, 61.1, 14.5; HRMS (APCI): *m*/*z* calcd. for $C_{50}H_{42}N_2O_{10}S_2$: 895.2354 ([M+H]$^+$); found: 895.2386.

Compound S8. To a solution of **S7** (214 mg, 0.239 mmol) in tetrahydrofuran (THF, 19 mL) was added a solution of NaOH (203 mg, 5.08 mmol) in water (19 mL). The reaction mixture was stirred at room temperature for 32.5 h. The resulting mixture was acidified with a 1 M aqueous solution of HCl (30 mL) and extracted with a 1:1 mixture of $CHCl_3$ and acetone. The combined organic layer was dried over Na_2SO_4. After filtration, the solvent was removed under reduced pressure to

afford **S8** as a yellow solid (191 mg, >99%), which was used in the next step without further purification.
^{1}H-NMR (400 MHz, DMSO-d_6) δ 8.09 (d, J = 2.0 Hz, 1H), 7.97–7.85 (m, 10H), 7.58 (d, J = 2.4 Hz, 1H), 7.40 (dd, J = 8.6, 1.8 Hz, 1H), 7.34 (dd, J = 8.8, 1.6 Hz, 1H), 7.22 (d, J = 8.8 Hz, 4H), 7.16 (d, J = 8.4 Hz, 4H). HRMS (ESI): m/z calcd. for $C_{42}H_{26}N_2O_{10}S_2$: 805.0921 ([M+Na]$^+$); found: 805.0920.

Compound 1. To a solution of compound **S8** (78.1 mg, 99.7 µmol), **S3** (141 mg, 0.618 mmol), N-TBTU (197 mg, 0.614 mmol) in N,N-dimethylformamide (DMF, 5.3 mL) was added DIEA (180 mg, 1.39 mmol), and the reaction mixture was stirred at room temperature for 6 days. The solvent was then removed under reduced pressure. The crude product was purified by silica gel column chromatography using a 20:1 mixture of $CHCl_3$ and MeOH as an eluent, followed by preparative GPC (DMF as eluent), to afford **1** as a yellow solid (119 mg, 73.5 µmol, 74%).
Mp: 151.5–152.0 °C; ^{1}H NMR (400 MHz, DMSO-d_6): δ 8.42 (d, J = 7.6 Hz, 2H), 8.35 (d, J = 7.2 Hz, 2H), 7.98–7.77 (m, 15H), 7.38 (d, J = 1.6 Hz, 1H), 7.28 (d, J = 8.0 Hz, 2H), 7.21 (d, J = 8.8 Hz, 4H), 7.13 (d, J = 8.4 Hz, 4H), 4.48–4.37 (m, 4H), 3.13–3.03 (m, 8H), 1.54–1.36 (m, 12H), 1.35–1.28 (m, 12H), 1.27–1.02 (m, 28H), 0.86–0.79 (m, 36H); ^{13}C NMR (100 MHz, DMSO-d_6): δ172.12, 172.06, 165.2, 149.0, 148.8, 148.1, 144.8, 144.1, 143.2, 143.1, 131.6, 130.2, 129.5, 129.3, 129.0, 127.1, 126.0, 124.8, 124.2, 123.0, 122.0, 121.2, 120.4, 115.5, 49.0, 49.0, 36.6, 36.2, 29.8, 27.4, 24.1, 22.6, 22.5, 19.4, 18.2, 18.1; HRMS (MALDI): m/z calcd. for $C_{94}H_{130}N_{10}O_{10}S_2$: 1622.9407 ([$M$]$^+$); found: 1622.9411.

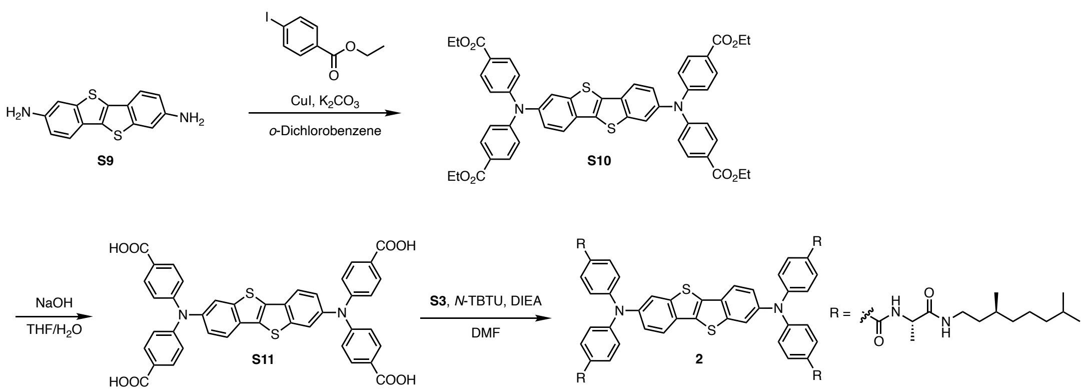

Supplementary Fig. 3. Synthesis of **2**.

Compound S10. 2,7-Diamino[1]benzothieno[3,2-b][1]benzothiophene (**S9**) was synthesized according to previously reported procedures.[6,7] Ethyl 4-iodobenzoate (30.5 g, 110 mmol), **S9** (1.51 g, 5.58 mmol), CuI (1.11 g, 5.81 mmol) and K_2CO_3 (7.70 g, 55.7 mmol) were dissolved in anhydrous o-dichlorobenzene (15 mL). The reaction mixture was stirred in at 195 °C for 9 days. After the reaction, methanol was added to remove o-dichlorobenzene, and the residue was dissolved in chloroform. The solvent was then removed under reduced pressure. The crude product was purified by silica gel column chromatography using a 4:5 mixture of dichloromethane and hexane (R_f = 0.30) as the eluent, followed by preparative GPC ($CHCl_3$), to afford **S10** as a white solid (381 mg, 442 µmol, 8%).

Mp: 143.0 –143.5 °C; ^{1}H NMR (400 MHz, $CDCl_3$): δ 7.98–7.93 (m, 8H), 7.79 (d, J = 8.4 Hz, 2H), 7.65 (d, J = 1.6 Hz, 2H), 7.24 (partially overlaps with a proton peak of solvent), 7.18–7.12 (m, 8H), 4.37 (q, J = 7.2 Hz, 8H), 1.39 (t, J = 7.0 Hz, 12H); ^{13}C NMR (100 MHz, $CDCl_3$): δ 166.2, 151.0, 143.8, 143.7, 133.4, 131.2, 130.4, 125.0, 124.1, 124.0, 122.9, 122.8, 122.6, 121.5, 121.4, 61.0, 14.5; HRMS (ESI): *m*/*z* calcd. for $C_{50}H_{42}N_2O_8S_2$: 885.2275 ([*M*+Na]$^+$); found: 885.2271.

Compound S11. A solution of NaOH (188 mg, 4.71 mmol) in water (15 mL) was added to a solution of **S10** (162 mg, 0.188 mmol) in THF (15 mL), and the reaction mixture was stirred at 38 °C for 3.5 days. The resulting mixture was then acidified with a 1 M aqueous solution of HCl (50 mL). The resulting precipitate was collected by filtration and dried under reduced pressure to afford **S11** as a white solid (126 mg, 90%), which was used in the next step without further purification.
^{1}H NMR (400 MHz, DMSO-d_6) δ 8.06 (d, J = 8.0 Hz, 2H), 8.00 (d, J = 1.6 Hz, 2H), 7.89 (d, J = 8.8 Hz, 8H), 7.30 (dd, J = 8.6, 2.2 Hz, 2H), 7.15 (d, J = 8.8 Hz, 8H); HRMS (ESI): *m*/*z* calcd. for $C_{42}H_{26}N_2O_8S_2$: 773.1023 ([*M*+Na]$^+$); found: 773.1016.

Compound 2. To a solution of **S11** (59.3 mg, 79.0 μmol), **S3** (117 mg, 0.511 mmol), and *N*-TBTU (168 mg, 0.523 mmol) in DMF (4.2 mL) was added DIEA (160 mg, 1.23 mmol), and the reaction mixture was stirred at room temperature for 4 days. The solvent was then removed under reduced pressure. The crude product was purified by silica gel column chromatography using a 15:1 mixture of CH_2Cl_2 and MeOH (R_f = 0.37) as an eluent, followed by preparative GPC (DMF as eluent), to afford **2** as a white solid (125 mg, 78.5 μmol, 99%).
Mp: 150.0 –150.9 °C; ^{1}H NMR (400 MHz, DMSO-d_6): δ 8.33 (d, J = 7.6 Hz, 4H), 8.00 (d, J = 8.8 Hz, 2H), 7.91–7.85 (m, 10H), 7.81 (t, J = 5.4 Hz, 4H), 7.23 (dd, J = 8.4, 2.0 Hz, 2H), 7.12 (d, J = 8.8 Hz, 8H), 4.48–4.37 (m, 4H), 3.13–3.02 (m, 8H), 1.55–1.35 (m, 12H), 1.30 (d, J = 7.2 Hz, 12H), 1.27–1.00 (m, 28H), 0.87–0.77 (m, 36H); ^{13}C NMR (100 MHz, DMSO-d_6): δ 172.2, 165.3, 149.2, 143.8, 143.2, 132.6, 129.3, 128.6, 123.6, 122.7, 122.5, 120.9, 49.0, 36.7, 36.6, 36.2, 29.8, 27.4, 24.1, 22.6, 22.5, 19.5, 18.2; HRMS (MALDI): *m*/*z* calcd. for $C_{94}H_{130}N_{10}O_8S_2$: 1590.9509 ([*M*]$^+$); found: 1590.9491.

Supplementary Figures

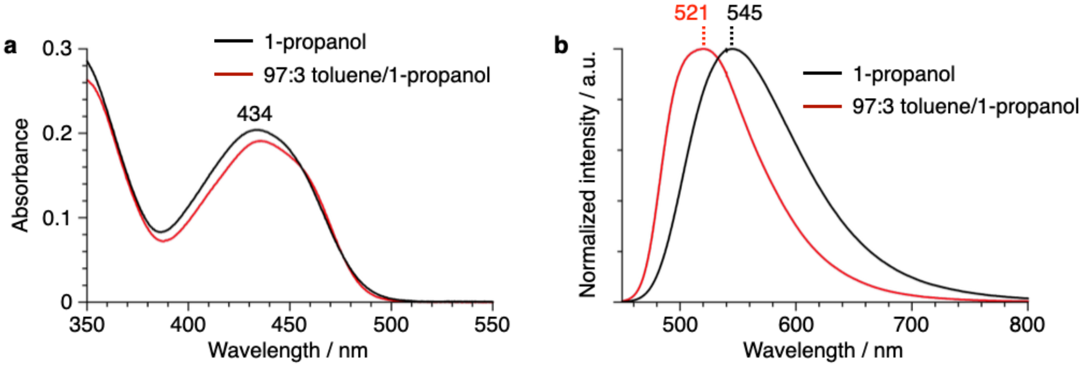

Supplementary Fig. 4. a UV–vis absorption and **b** fluorescence spectra of **1** in 1-propanol (black lines) and in 97:3 toluene/1-propanol (red lines) at a total concentration (c_T) of 1.0 × 10^{-5} M and temperature (T) of 293 K.

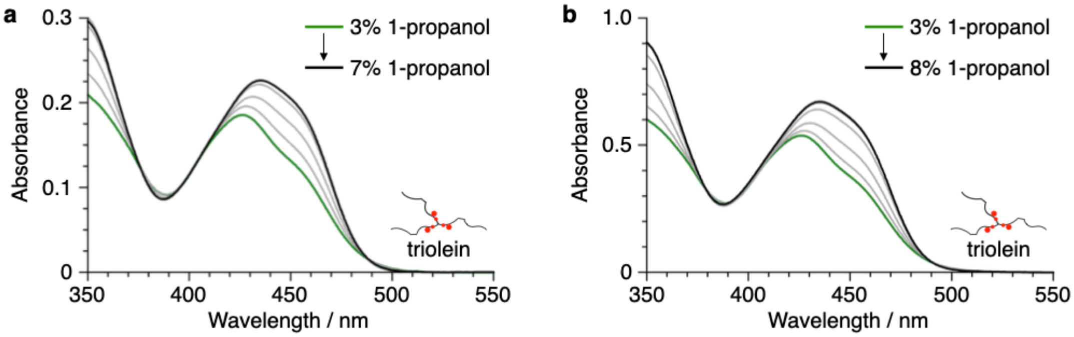

Supplementary Fig. 5. a, **b** Solvent-dependent UV–vis absorption spectra of **1** using triolein (TO) as the self-assembly-inducing solvent and 1-propanol as the denaturing agent at T = 293 K and (**a**) c_T = 2.5 × 10^{-5} M; (**b**) c_T = 4.0 × 10^{-5} M.

Supplementary Table 1. Thermodynamic parameters obtained by fitting the denaturation curves in Figures 2e, S6d, and S7d to the solvent-dependent cooperative model developed by *Meijer* and co-workers using a global fitting approach.[8]

	ΔG^0 / kJ mol^{-1}	ΔG^0 (SD) / kJ mol^{-1}	m / kJ mol^{-1}	m (SD) / kJ mol^{-1}	σ / 10^{-2}	σ (SD) / 10^{-2}
TO	–36.3	0.90	202	0.18	1.2	0.55
EO	–37.2	1.1	230	0.23	1.7	0.84
DBE	–37.6	0.34	123	4.4	1.6	0.32

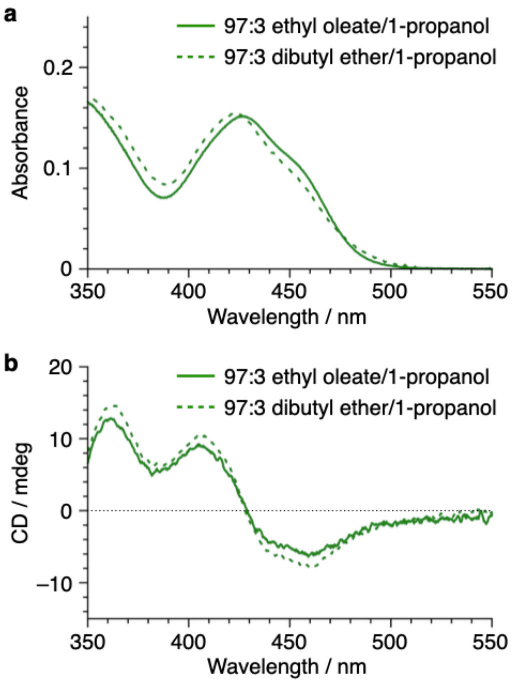

Supplementary Fig. 6. a UV–vis absorption and **b** CD spectra of $\mathbf{1}_{\mathbf{Agg}}$ in 97:3 ethyl oleate (EO)/1-propanol (solid lines) and 97:3 di-*n*-butyl ether (DBE)/1-propanol (dashed lines) at $c_T = 1.0 \times 10^{-5}$ M and $T = 293$ K.

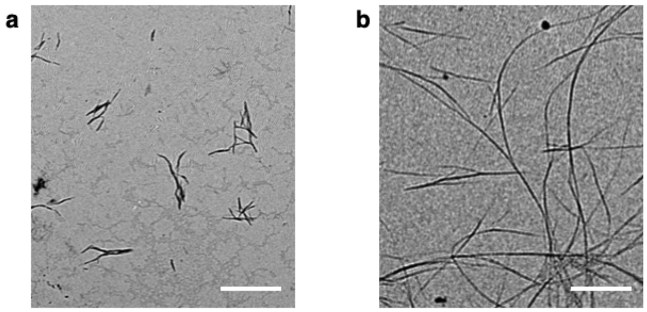

Supplementary Fig. 7. a, **b** TEM images of $\mathbf{1}_{\mathbf{Agg}}$ formed in 97:3 DBE/1-propanol upon sample preparation (**a**) with sonication and (**b**) without sonication at $c_T = 1.0 \times 10^{-5}$ M and $T = 293$ K; scale bars: 2 μm.

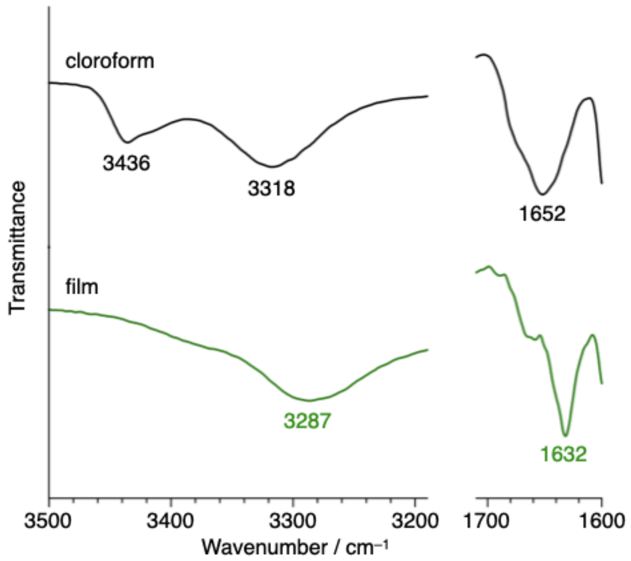

Supplementary Fig. 8. FT-IR spectra of a solution of **1** in chloroform ($c_T = 6.0 \times 10^{-4}$ M, black line) and a film prepared by drop-casting a solution of $\mathbf{1}_{\mathbf{Agg}}$ in 97:3 DBE/1-propanol ($c_T = 1.0 \times 10^{-5}$ M, green line).

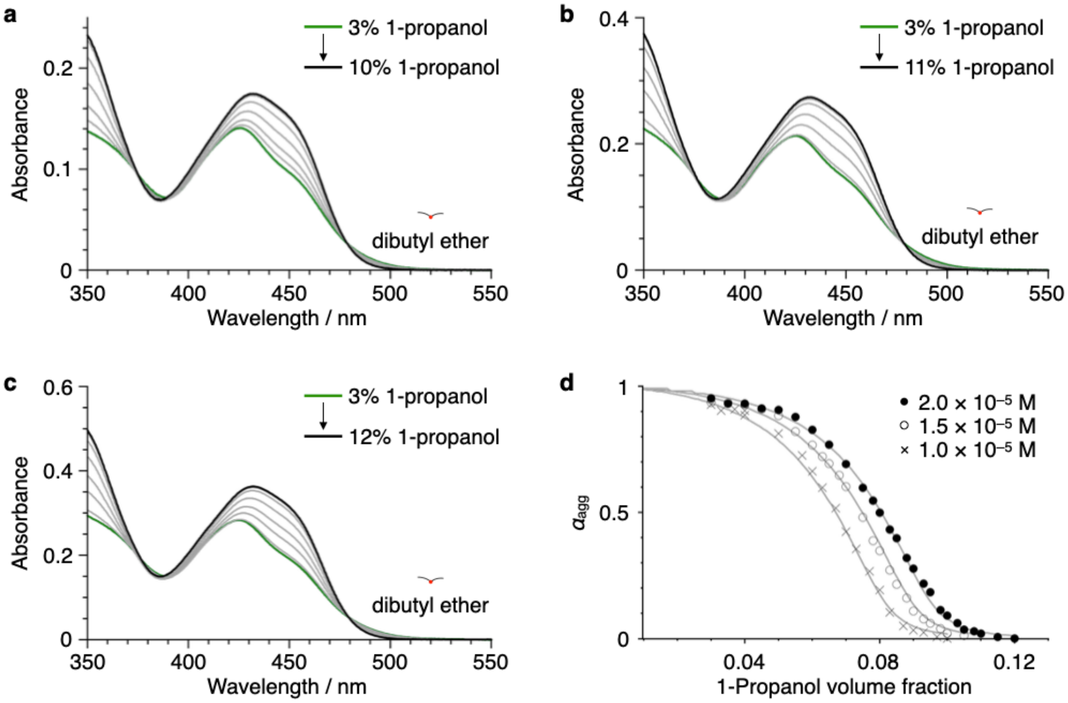

Supplementary Fig. 9. **a**–**c** Solvent-dependent UV–vis absorption spectra of **1** using DBE as the self-assembly-inducing solvent and 1-propanol as the denaturing agent at T = 293 K; **a** $c_T = 5.0 \times 10^{-6}$ M; **b** $c_T = 1.5 \times 10^{-5}$ M; **c** $c_T = 2.0 \times 10^{-5}$ M. **d** Aggregation parameter (α_{agg}) derived from the absorbance at λ_{abs} = 433 nm, plotted against the volume fraction of 1-propanol, fitted using the solvent-dependent cooperative model with a global fitting approach.[8]

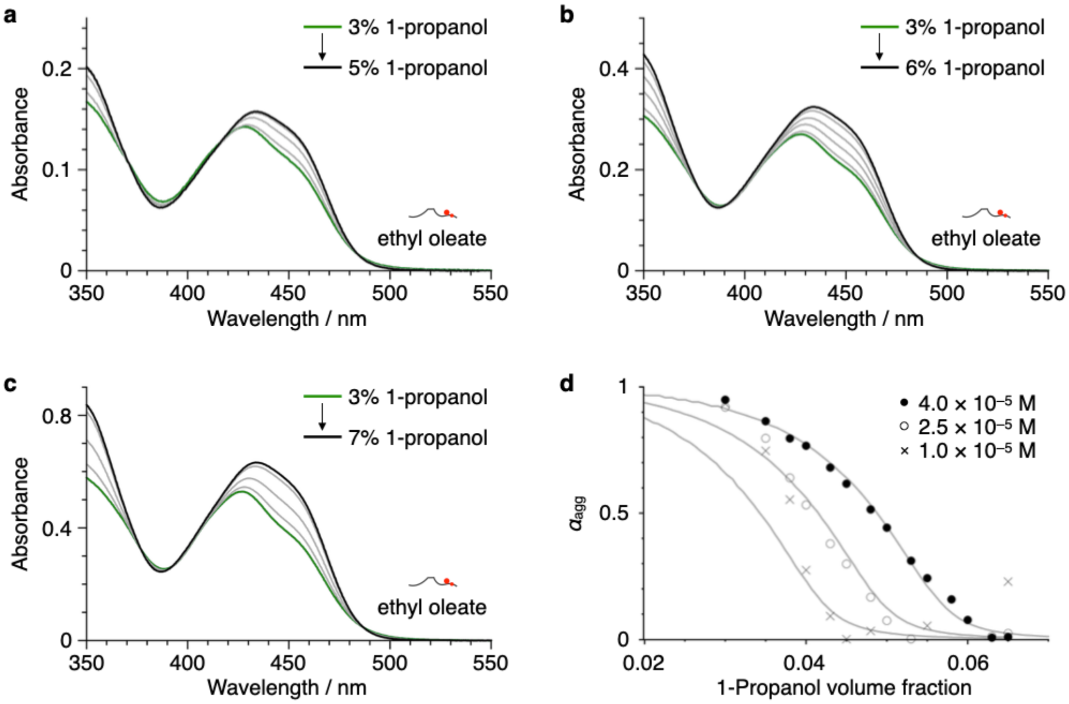

Supplementary Fig. 10. a–c Solvent-dependent UV–vis absorption spectra of **1** using EO as the self-assembly-inducing solvent and 1-propanol as the denaturing agent at $T = 293$ K; **a** $c_T = 1.0 \times 10^{-5}$ M; **b** $c_T = 2.5 \times 10^{-5}$ M; **c** $c_T = 4.0 \times 10^{-5}$ M. **d** Aggregation parameter (α_{agg}) derived from the absorbance at $\lambda_{abs} = 433$ nm, plotted against the volume fraction of 1-propanol, fitted using the solvent-dependent cooperative model with a global fitting approach.[8]

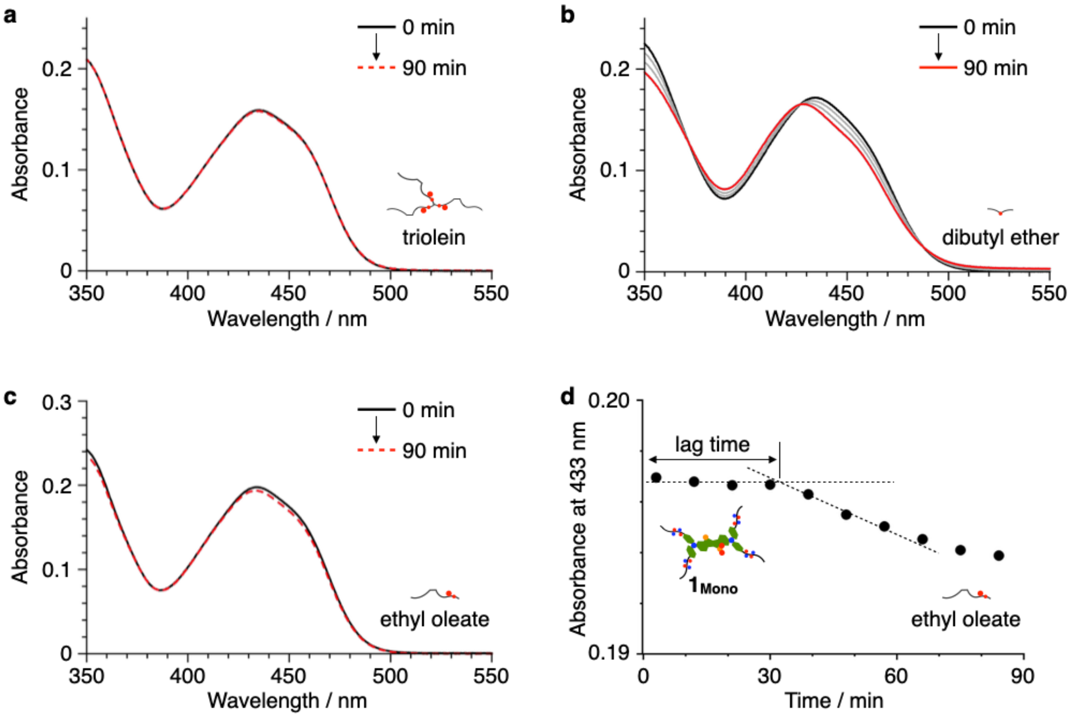

Supplementary Fig. 11. Time-dependent UV–vis absorption spectra of $\mathbf{1}_{\mathbf{Mono}}$ in **a** 97:3 TO/1-propanol, **b** 97:3 DBE/1-propanol, and **c** 97:3 EO/1-propanol at $c_T = 1.0 \times 10^{-5}$ M and $T = 293$ K. **d** Time-dependent changes in absorbance at 433 nm for $\mathbf{1}_{\mathbf{Mono}}$ in 97:3 EO/1-propanol at $c_T = 1.0 \times 10^{-5}$ M and $T = 293$ K.

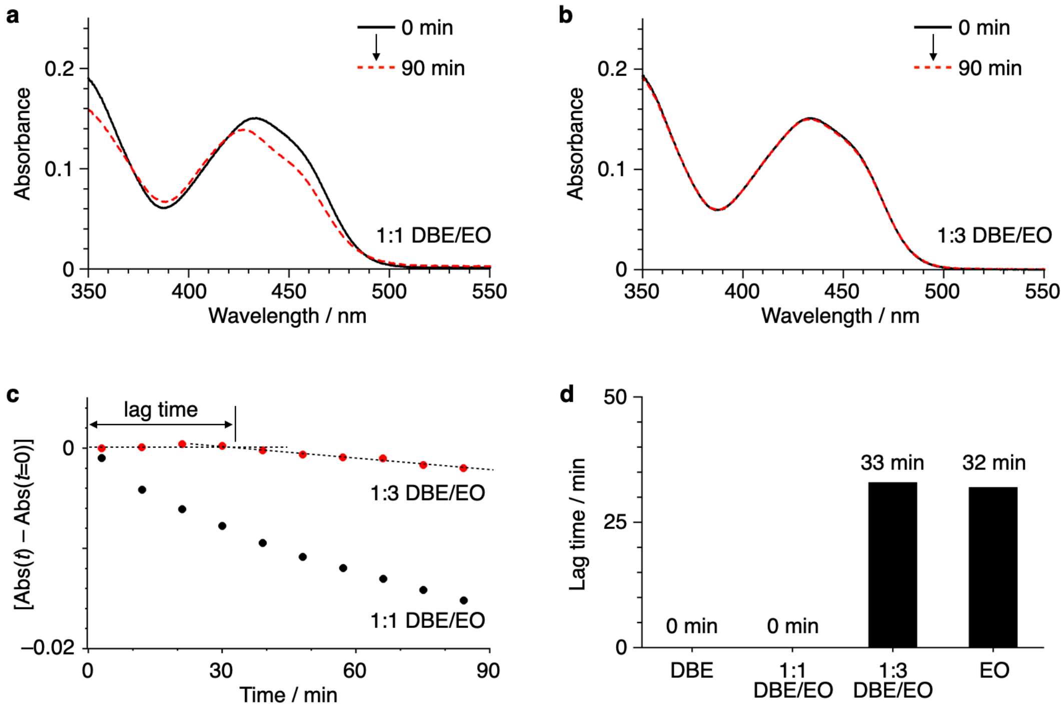

Supplementary Fig. 12. a,b Time-dependent UV–vis absorption spectra of $\mathbf{1}_{\mathbf{Mono}}$ in 1:1 DBE/EO (**a**) and 1:3 DBE/EO (**b**). **c** Time-dependent changes in absorbance at 433 nm for $\mathbf{1}_{\mathbf{Mono}}$ in 1:1 DBE/EO (black) and 1:3 DBE/EO (red), each containing 3 vol% 1-propanol, at $c_{\mathrm{T}} = 1.0 \times 10^{-5}$ M and $T = 293$ K. **d** Comparison of lag times before nucleation.

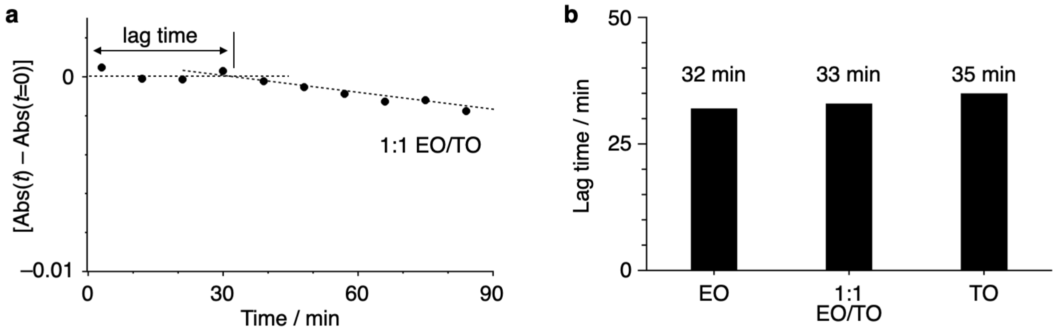

Supplementary Fig. 13. a Time-dependent changes in absorbance at 433 nm for $\mathbf{1}_{\mathbf{Mono}}$ in 1:1 EO/TO containing 3 vol% of 1-propanol, at $c_{\mathrm{T}} = 1.0 \times 10^{-5}$ M and $T = 293$ K. **b** Comparison of lag times before nucleation.

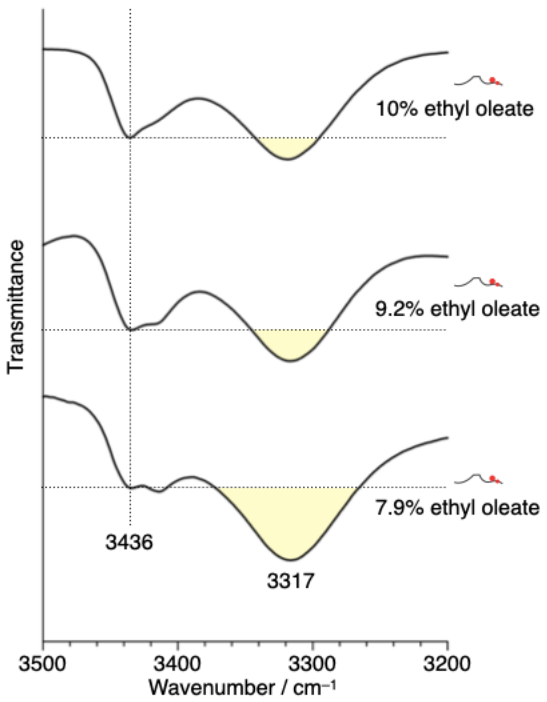

Supplementary Fig. 14. FT-IR spectra of $\mathbf{1}_{\mathbf{Mono}}$ in EO/chloroform mixtures with varying chloroform contents at $c_{\mathrm{T}} = 5.0 \times 10^{-4}$ M.

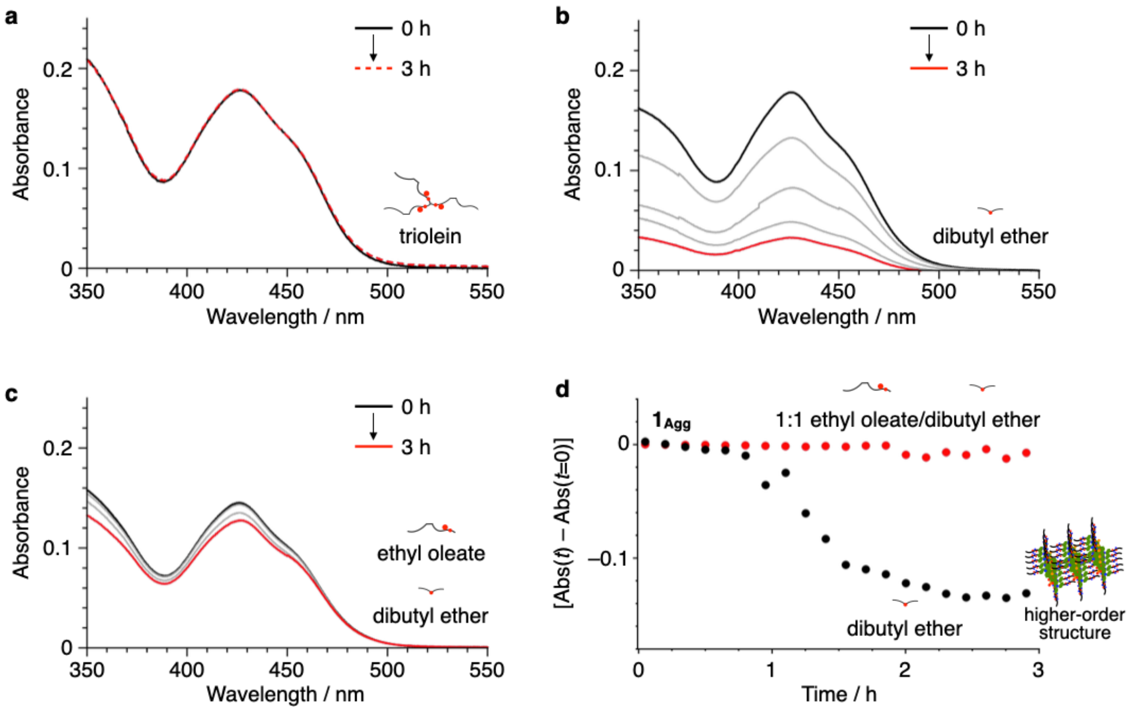

Supplementary Fig. 15. Time-dependent UV–vis absorption spectra of $\mathbf{1}_{\mathbf{Agg}}$ in **a** 97:3 TO/1-propanol, **b** 97:3 DBE/1-propanol, and **c** 48.5:48.5:3 DBE/EO/1-propanol at $c_T = 1.0 \times 10^{-5}$ M and T = 293 K. **d** Time-dependent changes in absorbance at 433 nm for $\mathbf{1}_{\mathbf{AggS}}$ in 48.5:48.5:3 DBE/EO/1-propanol ($c_T = 1.0 \times 10^{-5}$ M; red filled circles) and in 97:3 DBE/1-propanol ($c_T = 1.0 \times 10^{-5}$ M; black filled circles).

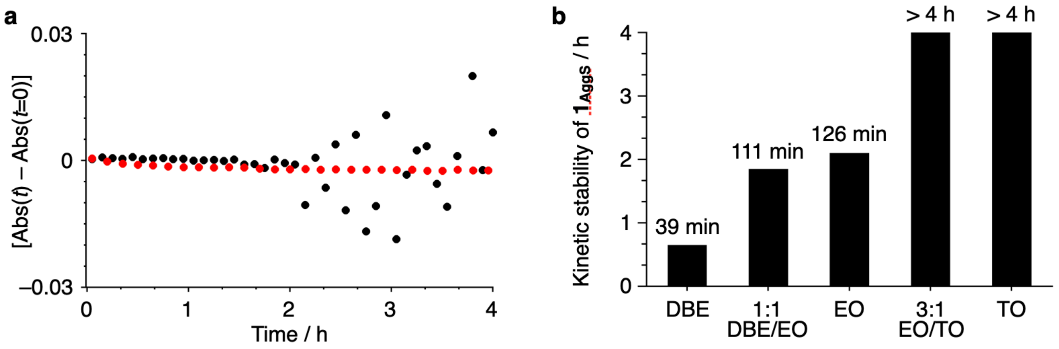

Supplementary Fig. 16. Time-dependent changes in absorbance at 433 nm for $\mathbf{1}_{\mathbf{AggS}}$ in EO (black) and in 3:1 EO/TO (red), each containing 3 vol% of 1-propanol, at $c_T = 1.0 \times 10^{-5}$ M and T = 293 K. **b** Comparison of the kinetic stability of $\mathbf{1}_{\mathbf{AggS}}$.

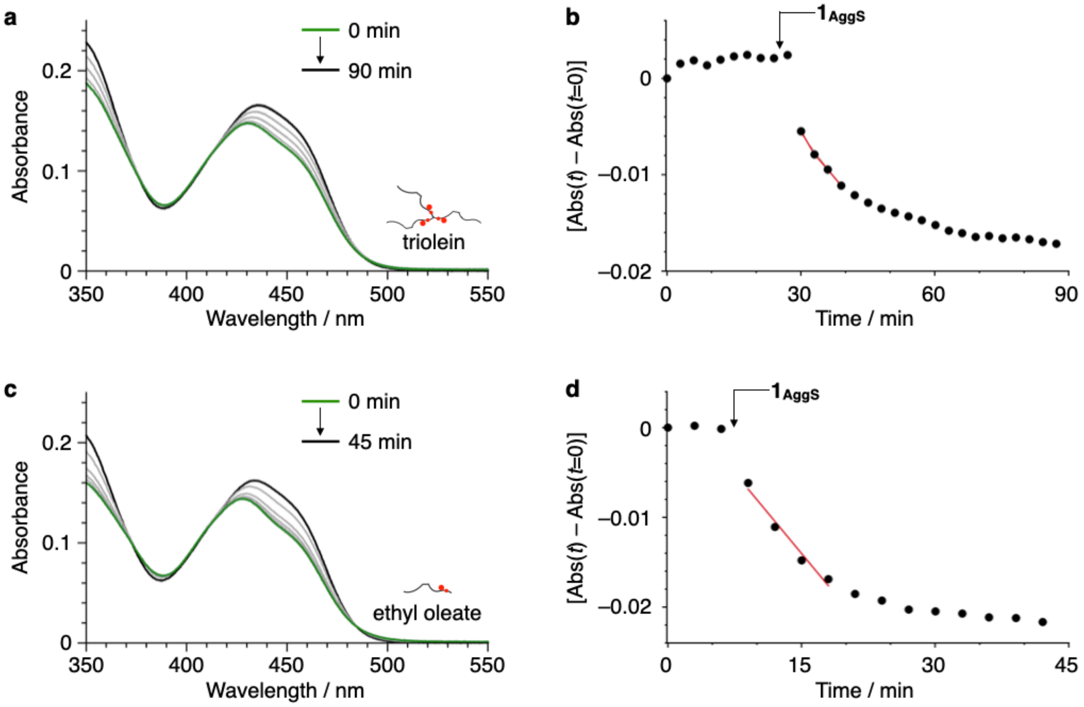

Supplementary Fig. 17. **a**, **c** Time-dependent UV–vis absorption spectra of **1**, and **b**, **d** change in absorbance at λ_{abs} = 433 nm before and after supramolecular polymerization initiated by addition of a solution of $\mathbf{1_{AggS}}$ (c_T = 1.0 × 10^{-5} M; 0.12 mL) to a solution of $\mathbf{1_{Mono}}$ (c_T = 1.0 × 10^{-5} M; 1.2 mL) in (**a**, **b**) 97:3 TO/1-propanol and (**c**, **d**) 97:3 EO/1-propanol at T = 293 K.

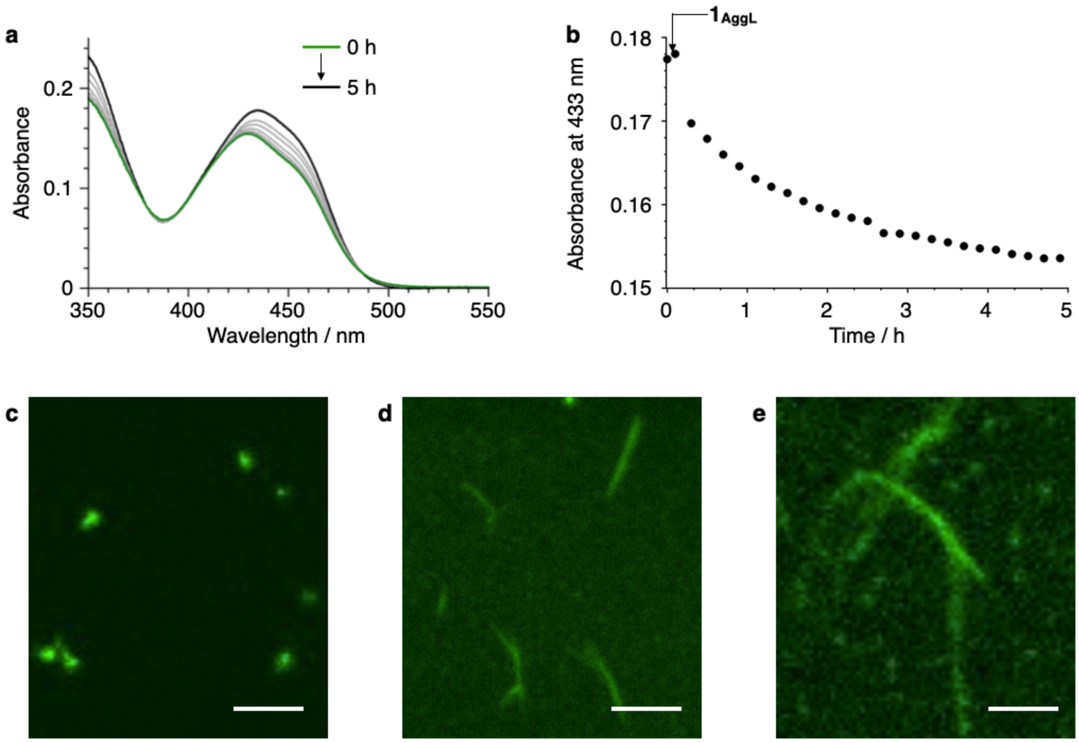

Supplementary Fig. 18. a Time-dependent UV–vis absorption spectra of **1**, and **b** change in absorbance at λ_{abs} = 433 nm before and after supramolecular polymerization initiated by addition of a solution of $\mathbf{1_{AggL}}$ (c_T = 1.0 × 10^{-5} M; 0.04 mL) to a solution of $\mathbf{1_{Mono}}$ (c_T = 1.0 × 10^{-5} M; 1.2 mL) in 97:3 TO/1-propanol at T = 293 K. **c**–**e** CLSM images of (**c**) $\mathbf{1_{AggS}}$, (**d**) $\mathbf{1_{AggL}}$, and (**e**) the resultant nanostructures; λ_{ex} = 405 nm; λ_{em} = 490–590 nm; scale bars: 3 µm.

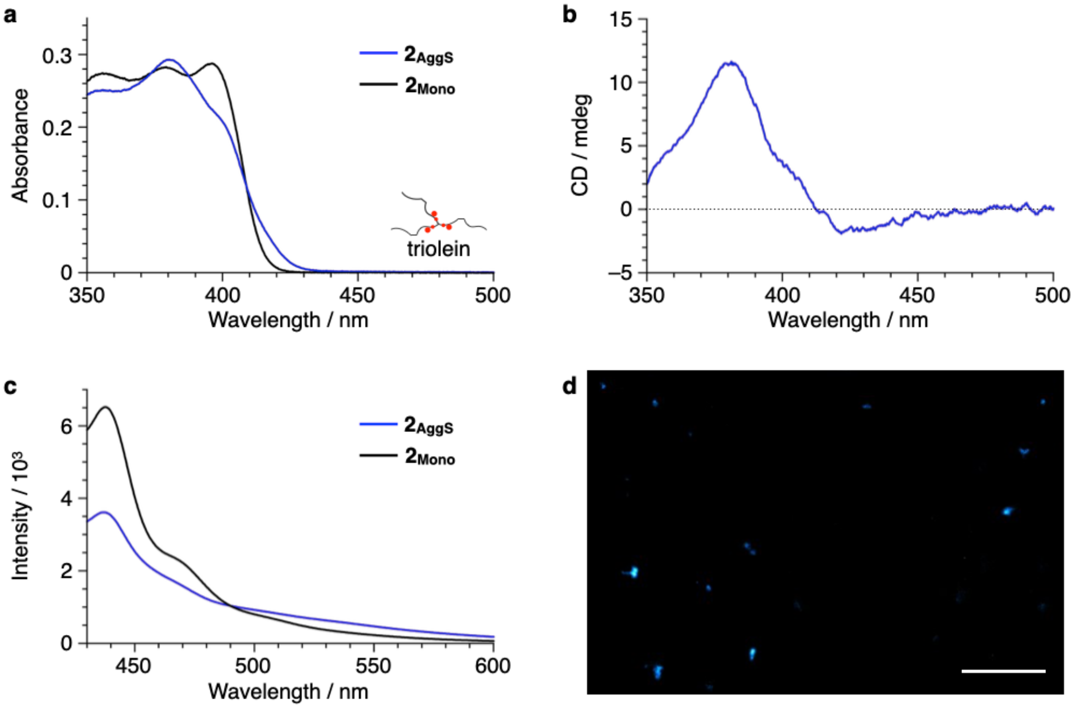

Supplementary Fig. 19. **a** UV–vis absorption, **b** CD, and **c** fluorescence spectra of **2** in 97:3 TO/1-propanol at $c_{\mathrm{T}} = 1.0 \times 10^{-5}$ M and $T = 293$ K before (black lines) and after sonication (blue lines). **d** CLSM image of $\mathbf{2}_{\mathbf{AggS}}$ obtained by sonication; $\lambda_{\mathrm{ex}} = 405$ nm; $\lambda_{\mathrm{em}} = 443$–$485$ nm; scale bar: 10 μm.

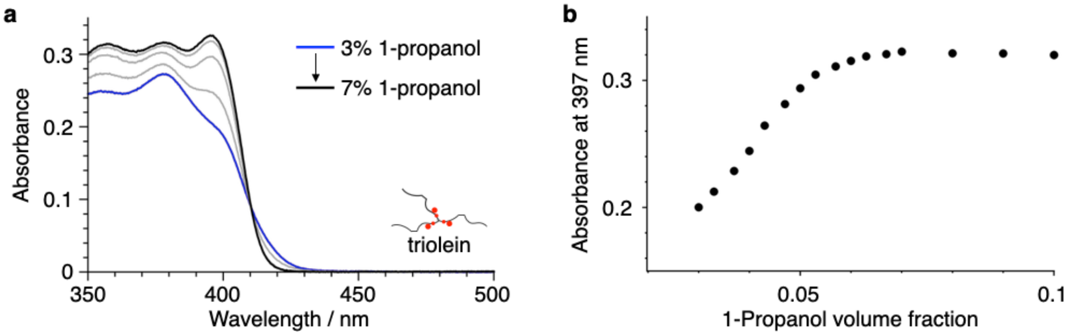

Supplementary Fig. 20. **a** Solvent-dependent UV–vis absorption spectra of **2**, and **b** the absorbance changes at $\lambda_{\mathrm{abs}} = 397$ nm in TO/1-propanol mixtures with varying 1-propanol contents from 3% to 8% at $c_{\mathrm{T}} = 1.0 \times 10^{-5}$ M and $T = 293$ K.

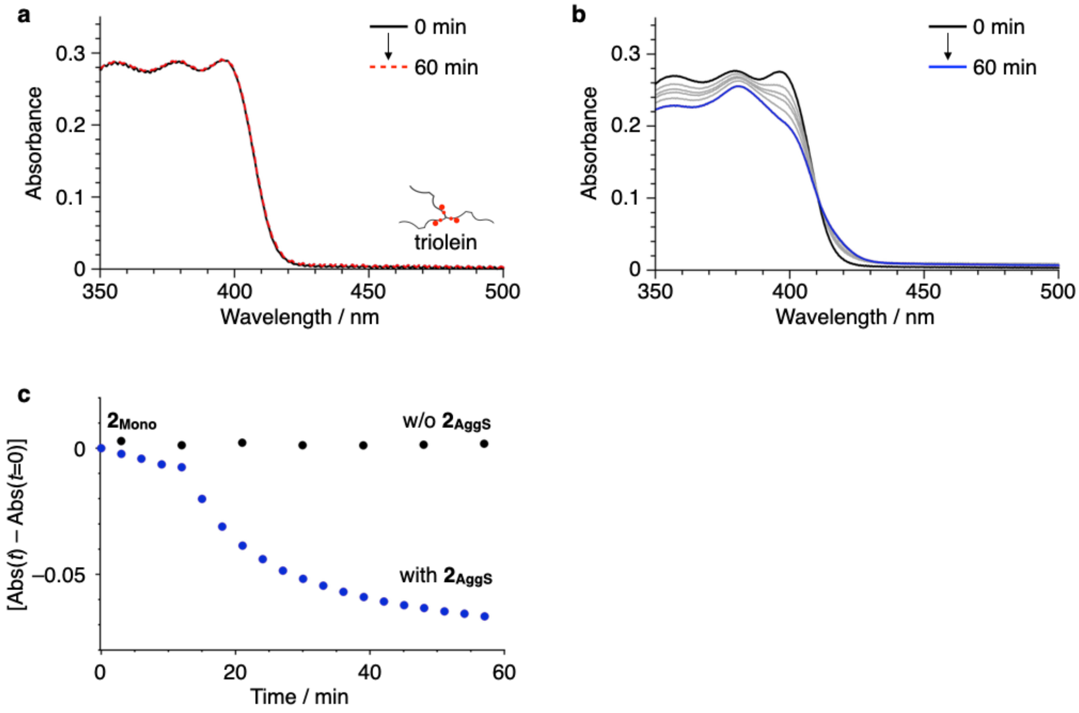

Supplementary Fig. 21. **a**, **b** Time-dependent UV–vis absorption spectra of (**a**) $\mathbf{2}_{\mathbf{Mono}}$ and (**b**) $\mathbf{2}_{\mathbf{Mono}}$ before and after addition of $\mathbf{2}_{\mathbf{AggS}}$ ($c_T = 1.0 \times 10^{-5}$ M; 0.12 mL) to a solution of $\mathbf{2}_{\mathbf{Mono}}$ ($c_T = 1.0 \times 10^{-5}$ M; 1.2 mL), and **c** absorbance change at $\lambda_{abs} = 397$ nm over time with $\mathbf{2}_{\mathbf{AggS}}$ (blue filled circles) or without (black filled circles) in 97:3 TO/1-propanol at $T = 293$ K.

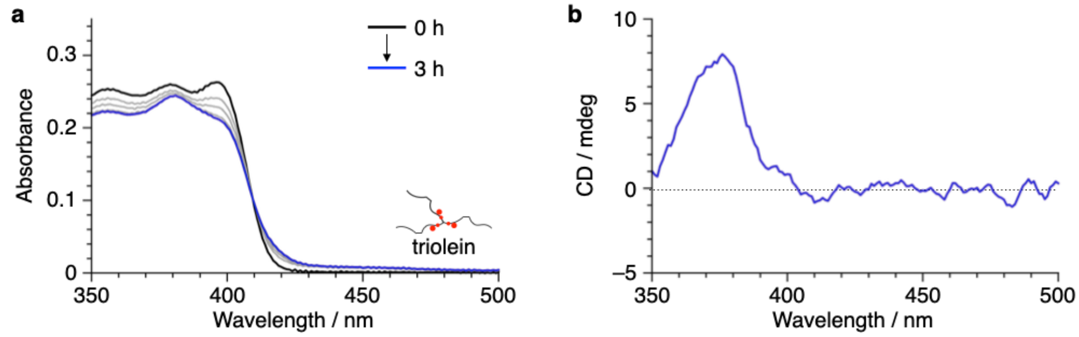

Supplementary Fig. 22. **a** Time-dependent UV–vis absorption spectra of **2** before and after supramolecular polymerization initiated by addition of $\mathbf{1}_{\mathbf{AggL}}$ ($c_T = 1.0 \times 10^{-5}$ M; 0.04 mL) to a solution of $\mathbf{2}_{\mathbf{Mono}}$ ($c_T = 1.0 \times 10^{-5}$ M; 1.2 mL) in 97:3 TO/1-propanol at $T = 293$ K, and **b** CD spectrum of the resultant solution.

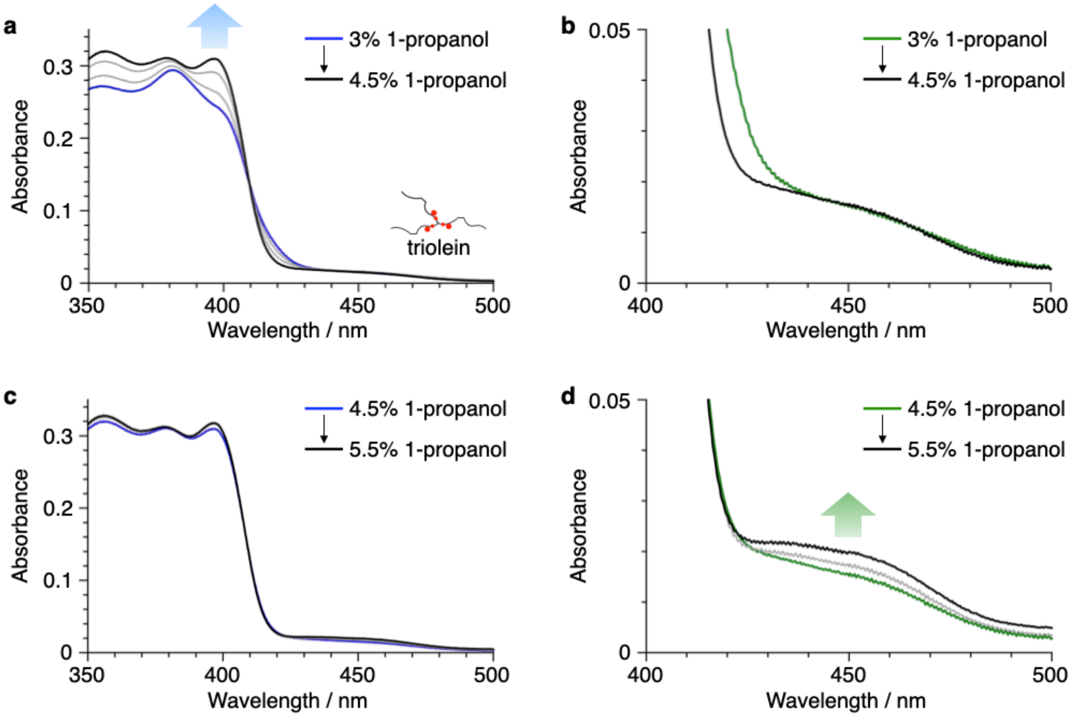

Supplementary Fig. 23. Solvent-dependent UV–vis absorption spectra of (**a**, **c**) **2** (350–430 nm) and (**b**, **d**) **1** (420–480 nm) in TO/1-propanol mixtures; (**a**, **b**) for 3.0–4.5% 1-propanol, (**c**, **d**) for 4.5–5.5% 1-propanol, at $c_T = 1.0 \times 10^{-5}$ M and $T = 293$ K.

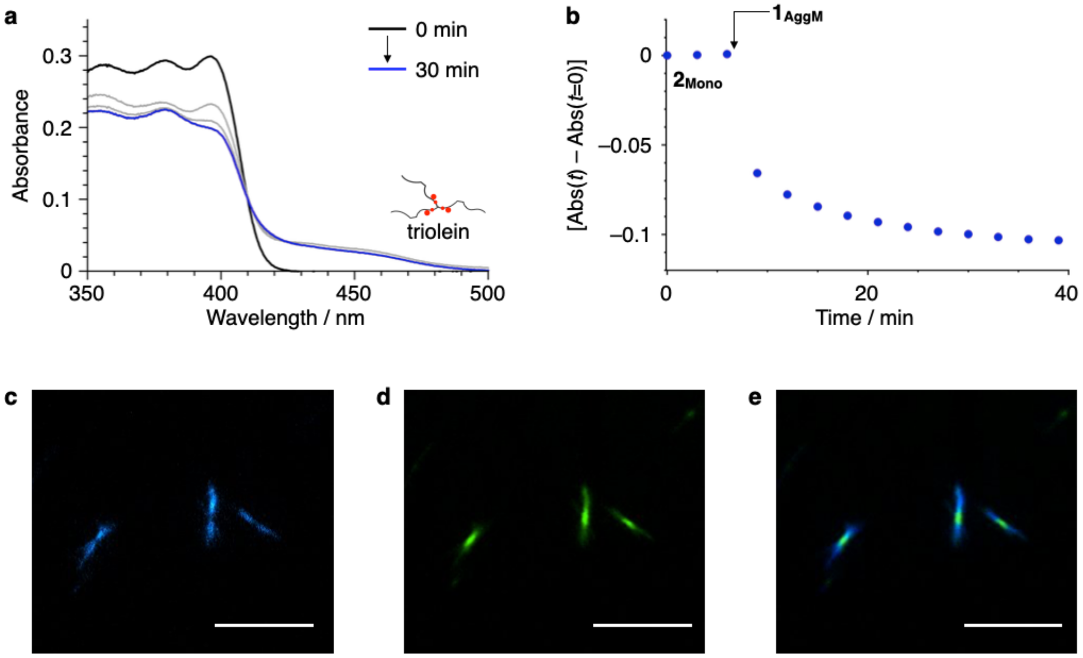

Supplementary Fig. 24. a Time-dependent UV–vis absorption spectra of $\mathbf{2}_{\mathbf{Mono}}$, and **b** change in absorbance at λ_{abs} = 397 nm before and after supramolecular polymerization initiated by addition of $\mathbf{1}_{\mathbf{AggM}}$ ($c_T = 1.0 \times 10^{-5}$ M; 0.40 mL) to $\mathbf{2}_{\mathbf{Mono}}$ ($c_T = 1.0 \times 10^{-5}$ M; 1.2 mL) in 97:3 TO/1-propanol at T = 293 K. $\mathbf{1}_{\mathbf{AggM}}$ was preformed by adding a solution of $\mathbf{1}_{\mathbf{AggS}}$ to a fresh solution of $\mathbf{1}_{\mathbf{Mono}}$ in 97:3 TO/1-propanol at a $\mathbf{1}_{\mathbf{Mono}}$/$\mathbf{1}_{\mathbf{AggS}}$ volume ratio of 3:1. **c**–**e** CLSM images of the resulting triblock copolymers; λ_{ex} = 405 nm; **c** blue channel: λ_{em} = 420-460 nm; **d** green channel: λ_{em} = 490–590 nm; scale bars: 5 µm.

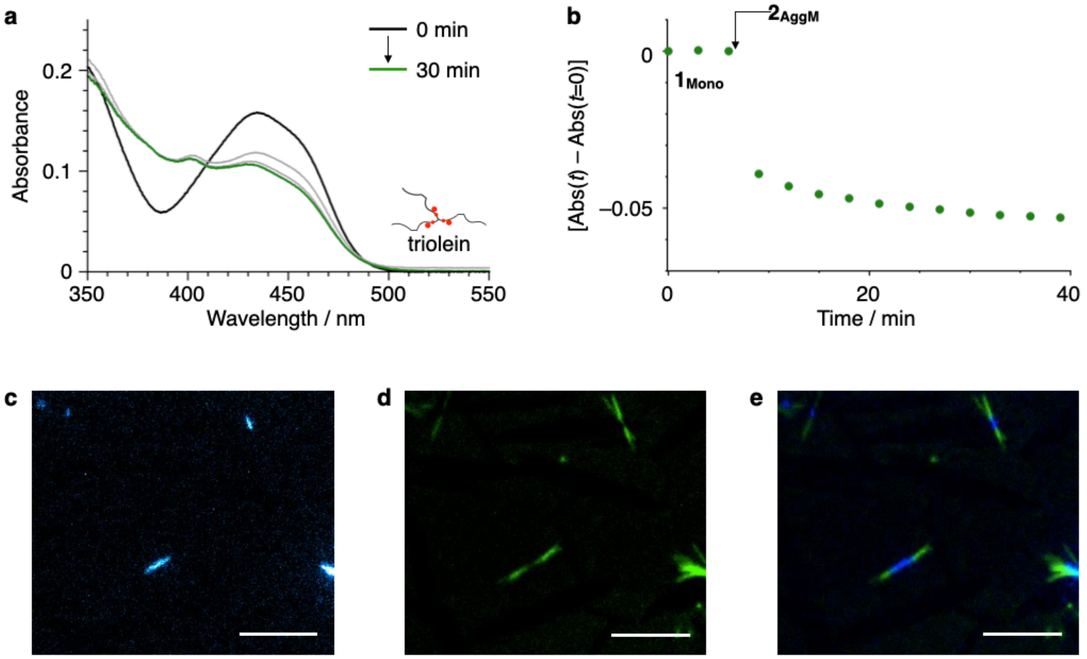

Supplementary Fig. 25. **a** Time-dependent UV–vis absorption spectra of $\mathbf{1}_{\mathbf{Mono}}$, and **b** change in absorbance at λ_{abs} = 433 nm before and after supramolecular polymerization initiated by addition of $\mathbf{2}_{\mathbf{AggM}}$ ($c_T = 1.0 \times 10^{-5}$ M; 0.40 mL) to $\mathbf{1}_{\mathbf{Mono}}$ ($c_T = 1.0 \times 10^{-5}$ M; 1.2 mL) in 97:3 TO/1-propanol at T = 293 K. $\mathbf{2}_{\mathbf{AggM}}$ was preformed by adding a solution of $\mathbf{2}_{\mathbf{AggS}}$ to a fresh solution of $\mathbf{2}_{\mathbf{Mono}}$ in 97:3 TO/1-propanol at a $\mathbf{2}_{\mathbf{Mono}}$/$\mathbf{2}_{\mathbf{AggS}}$ volume ratio of 3:1. **c**–**e** CLSM images of the resultant triblock copolymers; λ_{ex} = 405 nm; **c** blue channel: λ_{em} = 420-460 nm; **d** green channel: λ_{em} = 490–590 nm; scale bars: 5 µm.

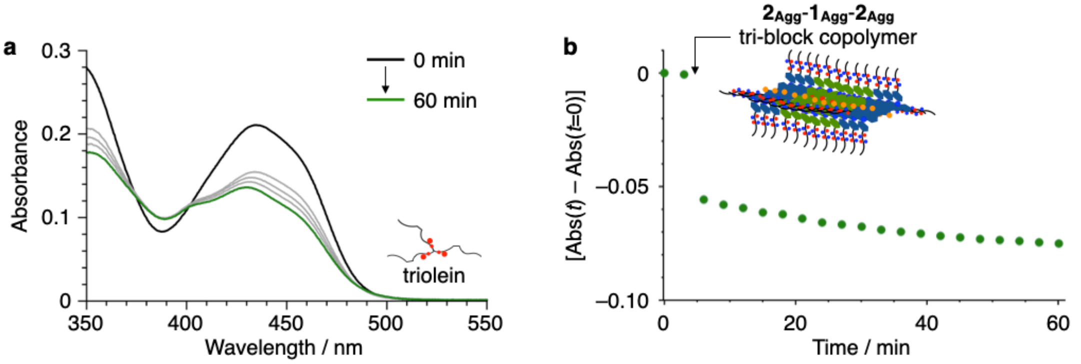

Supplementary Fig. 26. **a** Time-dependent UV–vis absorption spectra of $\mathbf{1}_{\mathbf{Mono}}$, and **b** change in absorbance at 433 nm before and after supramolecular polymerization initiated by the addition of a solution of the triblock copolymer $\mathbf{2}_{\mathbf{Agg}}$-$\mathbf{1}_{\mathbf{Agg}}$-$\mathbf{2}_{\mathbf{Agg}}$ (0.40 mL) to a solution of $\mathbf{1}_{\mathbf{Mono}}$ ($c_T = 1.0 \times 10^{-5}$ M; 1.2 mL) in 97:3 TO/1-propanol at T = 293 K.

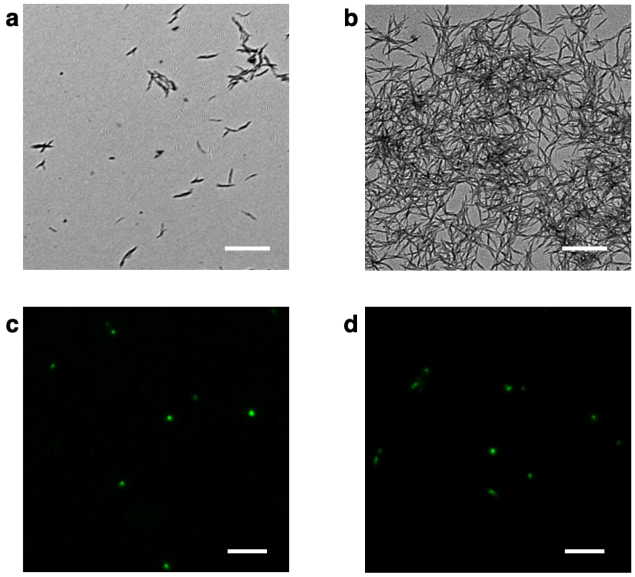

Supplementary Fig. 27. a,b TEM images of (**a**) $\mathbf{1}_{\mathbf{AggS}}$ formed in DBE at $c_T = 1.0 \times 10^{-5}$ M just after sonication and (**b**) bundled aggregates obtained after 120 min; scale bar: 2 µm. **c,d** CLSM images of $\mathbf{1}_{\mathbf{AggS}}$ in TO at $c_T = 1.0 \times 10^{-5}$ M obtained just after sonication (**c**) and after 4 h (**d**); λ_{ex} = 405 nm; λ_{em} = 490–590 nm; scale bar: 5 µm.

NMR spectroscopy

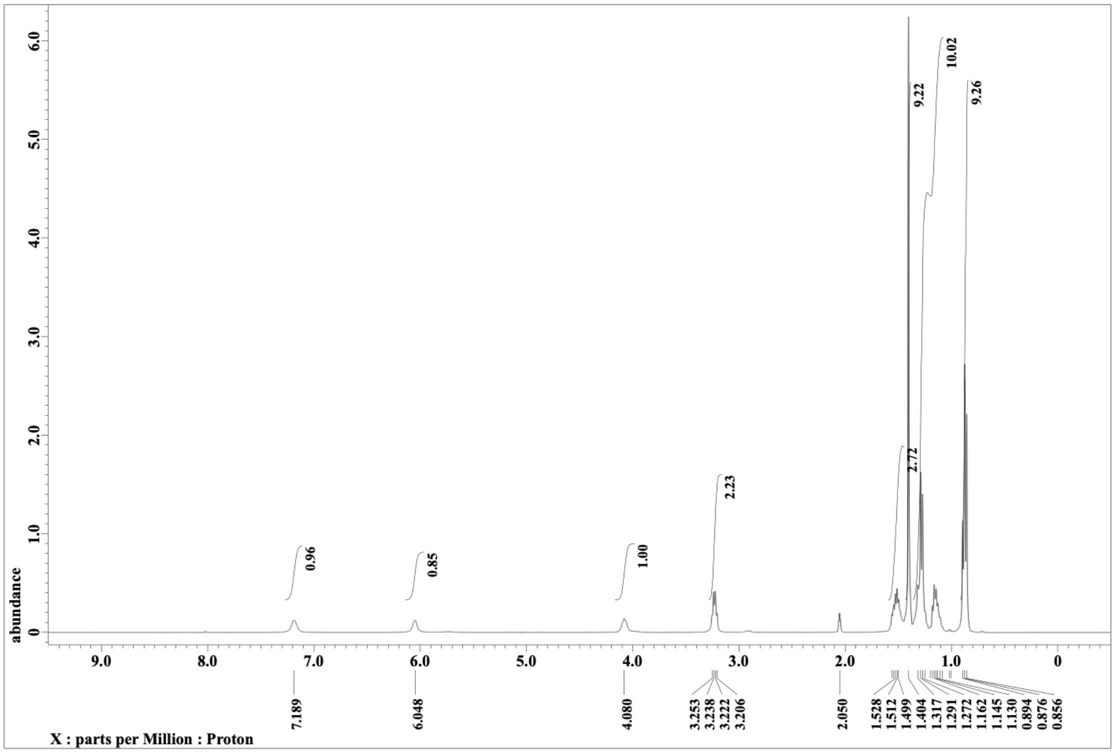

Supplementary Fig. 28. ^{1}H NMR spectrum of compound **S2** (400 MHz, 298 K, acetone-d_6).

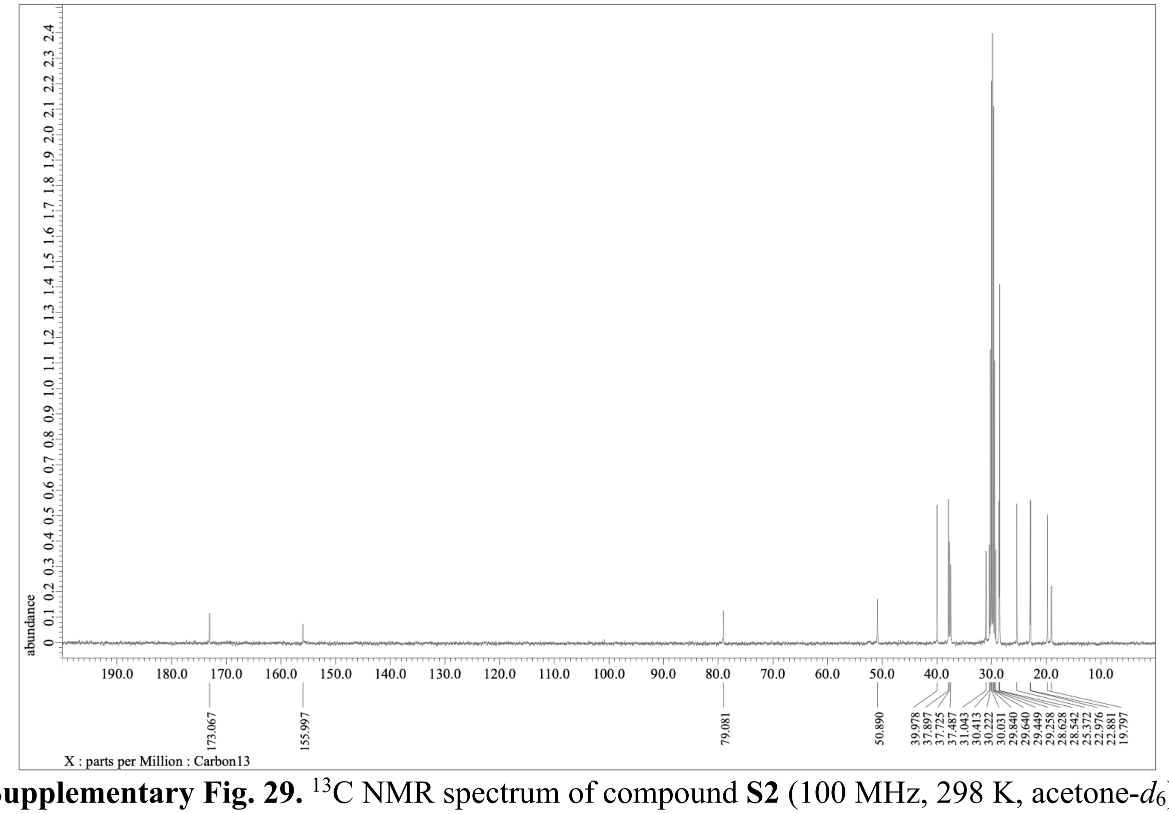

Supplementary Fig. 29. ^{13}C NMR spectrum of compound **S2** (100 MHz, 298 K, acetone-d_6).

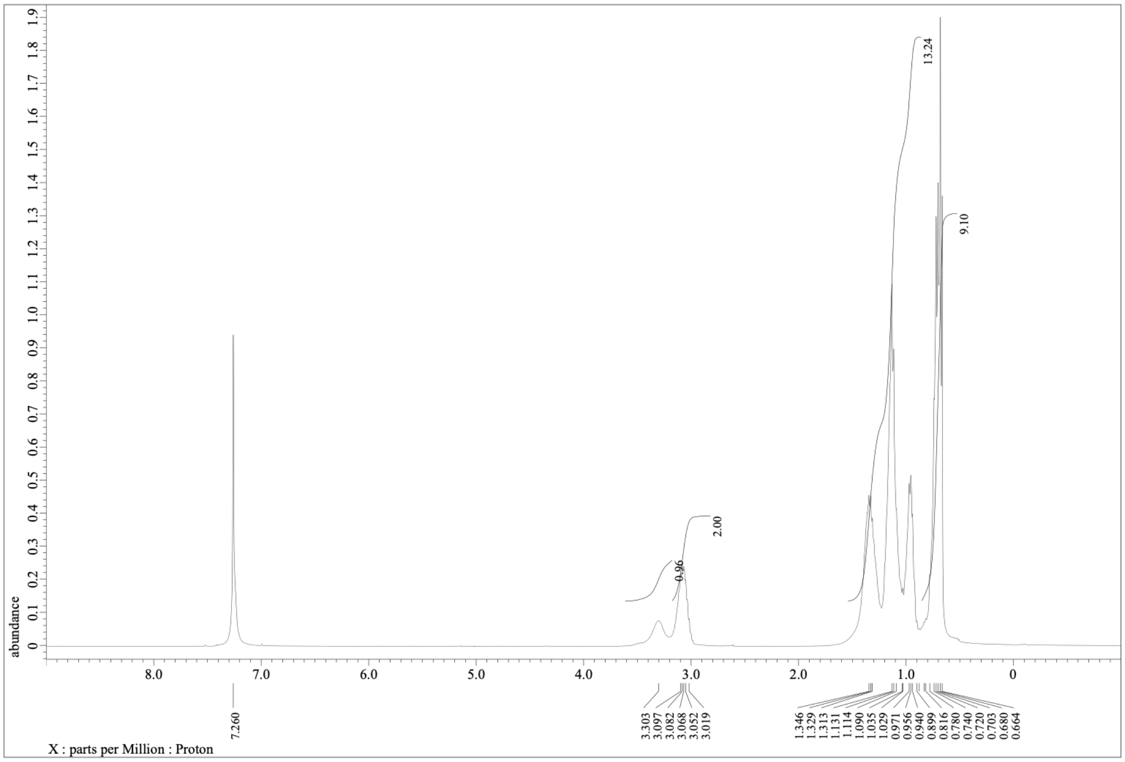

Supplementary Fig. 30. ^{1}H NMR spectrum of compound **S3** (400 MHz, 298 K, $CDCl_3$).

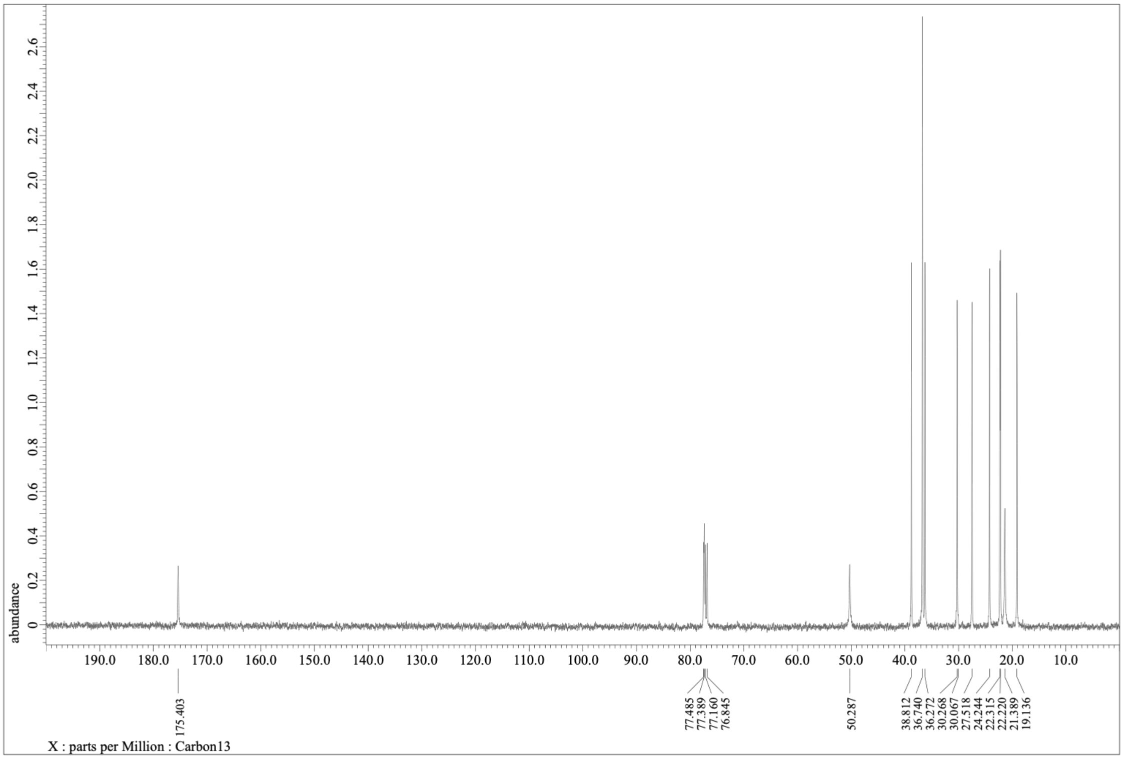

Supplementary Fig. 31. ^{13}C NMR spectrum of compound **S3** (100 MHz, 298 K, $CDCl_3$).

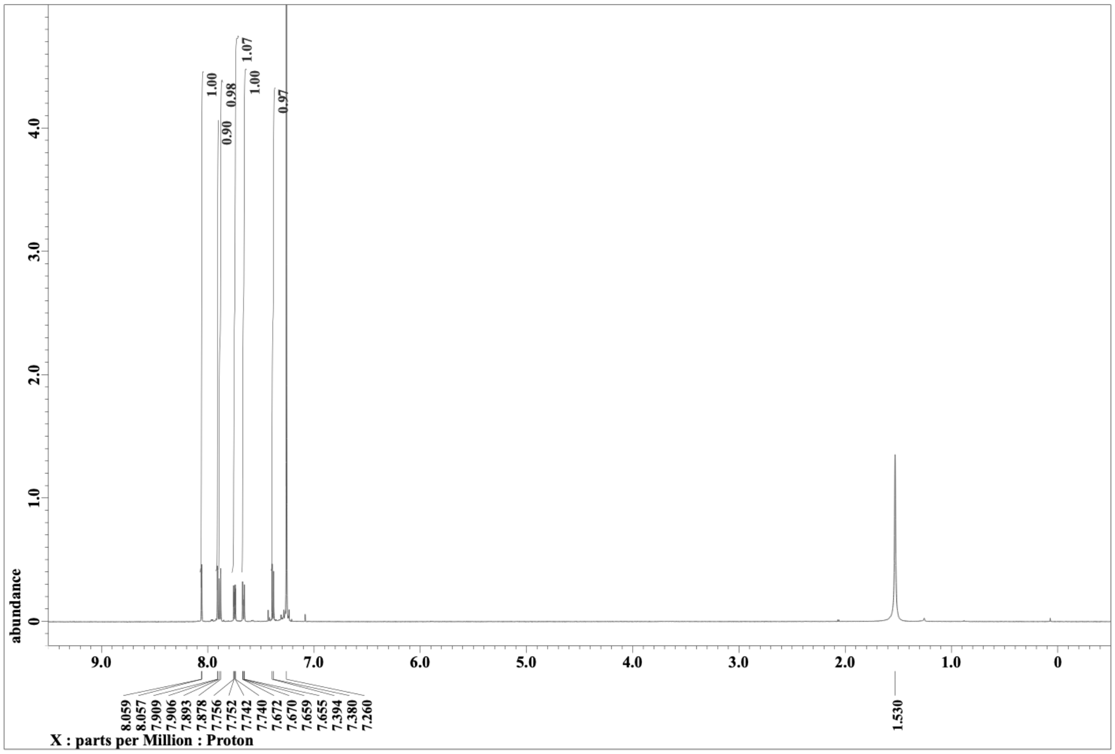

Supplementary Fig. 32. ^{1}H NMR spectrum of compound **S5** (600 MHz, 298 K, $CDCl_3$).

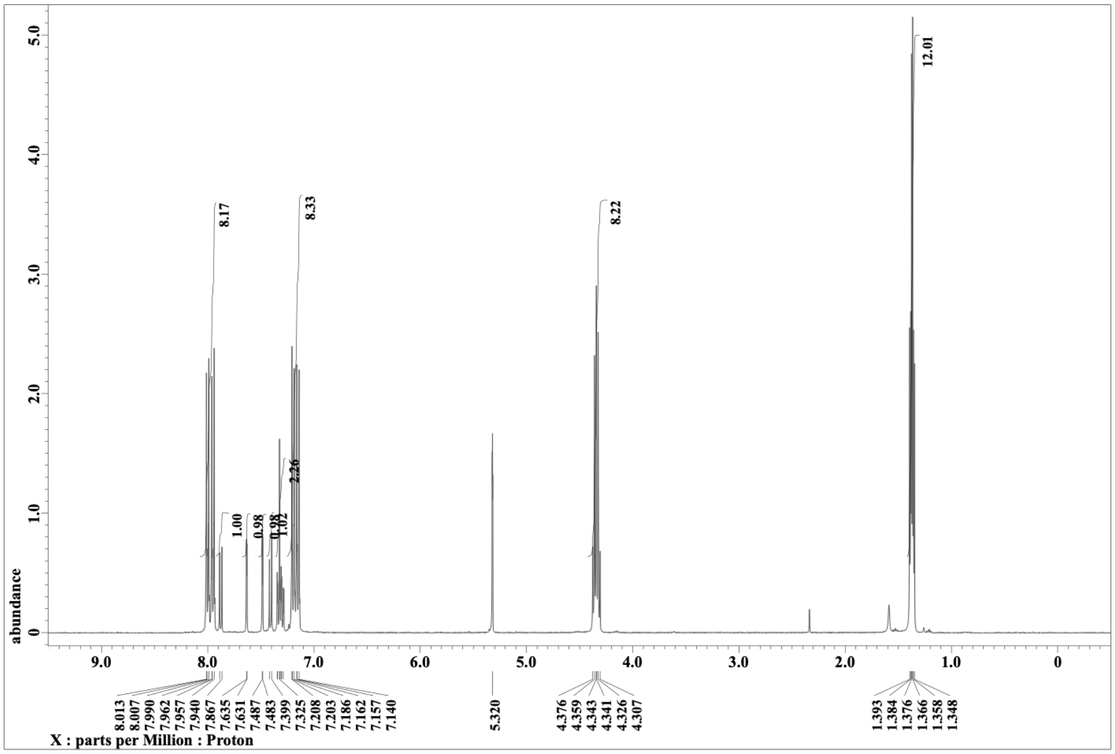

Supplementary Fig. 33. ^{1}H NMR spectrum of compound **S7** (400 MHz, 298 K, CD_2Cl_2).

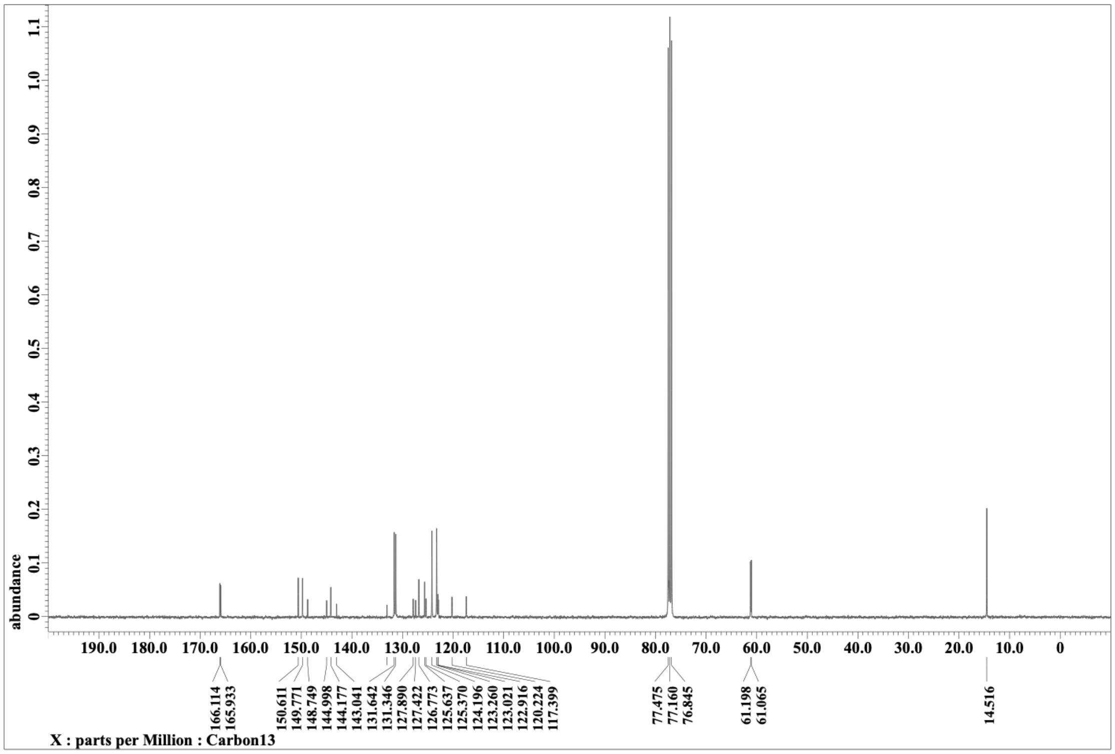

Supplementary Fig. 34. ^{13}C NMR spectrum of compound **S7** (100 MHz, 298 K, $CDCl_3$).

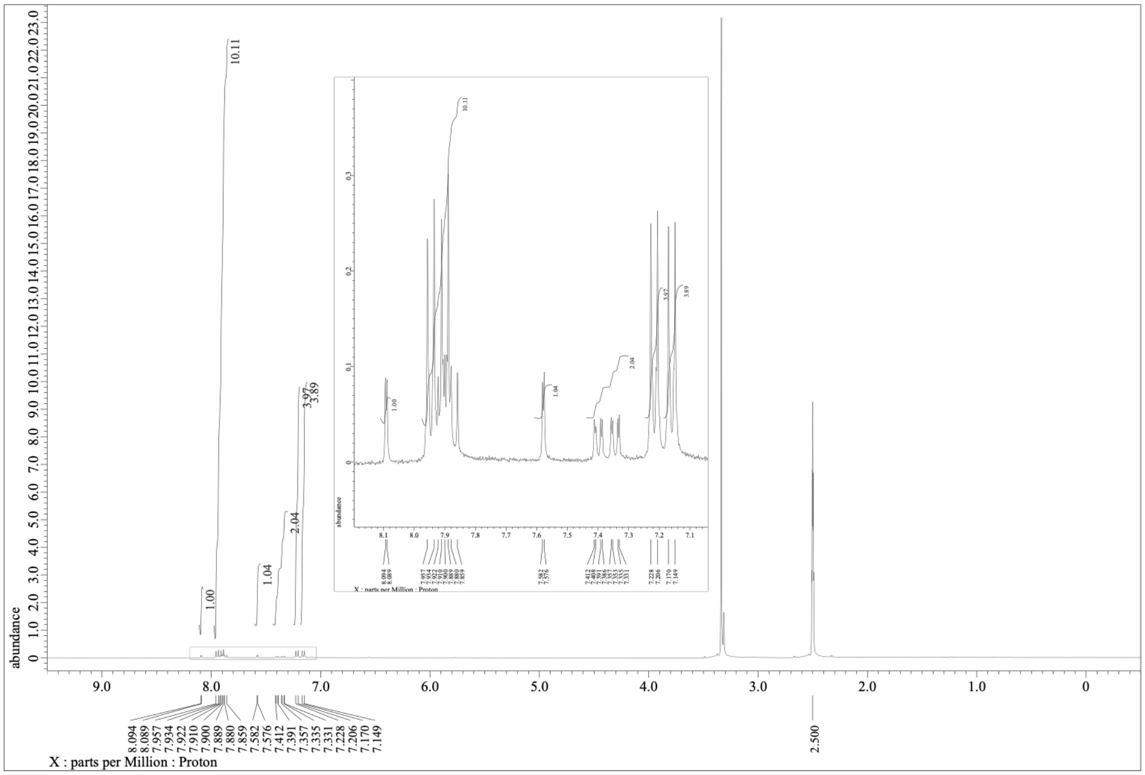

Supplementary Fig. 35. ^{1}H NMR spectrum of compound **S8** (400 MHz, 298 K, DMSO-d_6).

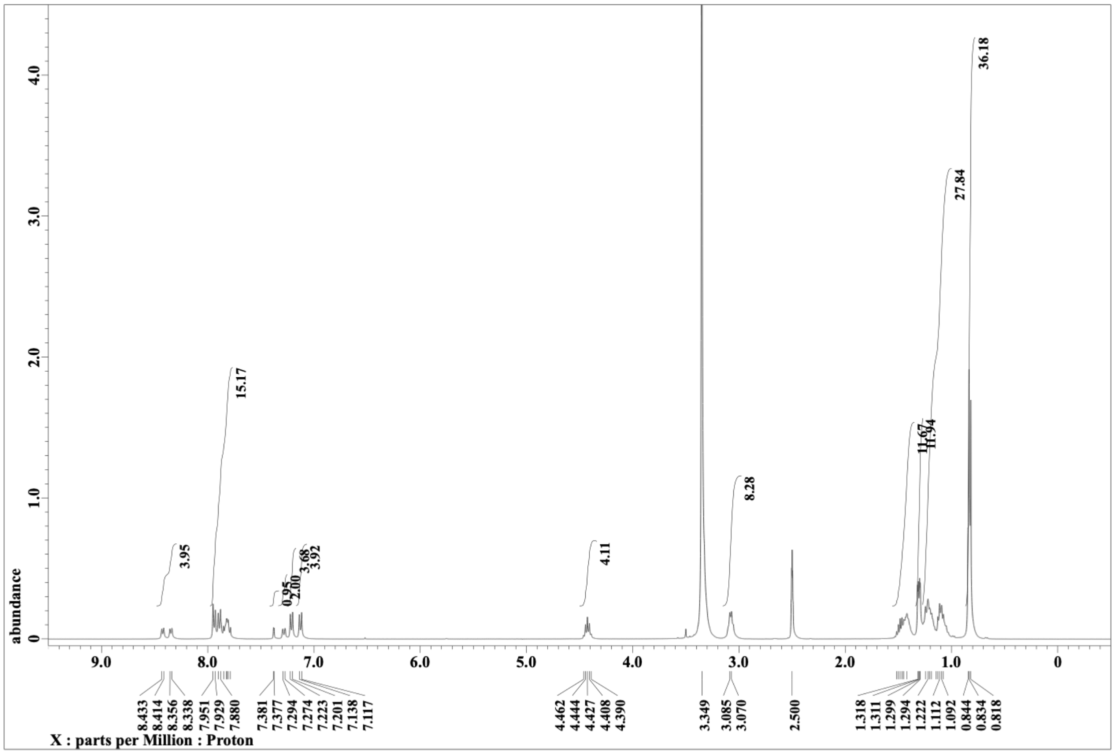

Supplementary Fig. 36. ^{1}H NMR spectrum of compound **1** (400 MHz, 298 K, DMSO-d_6).

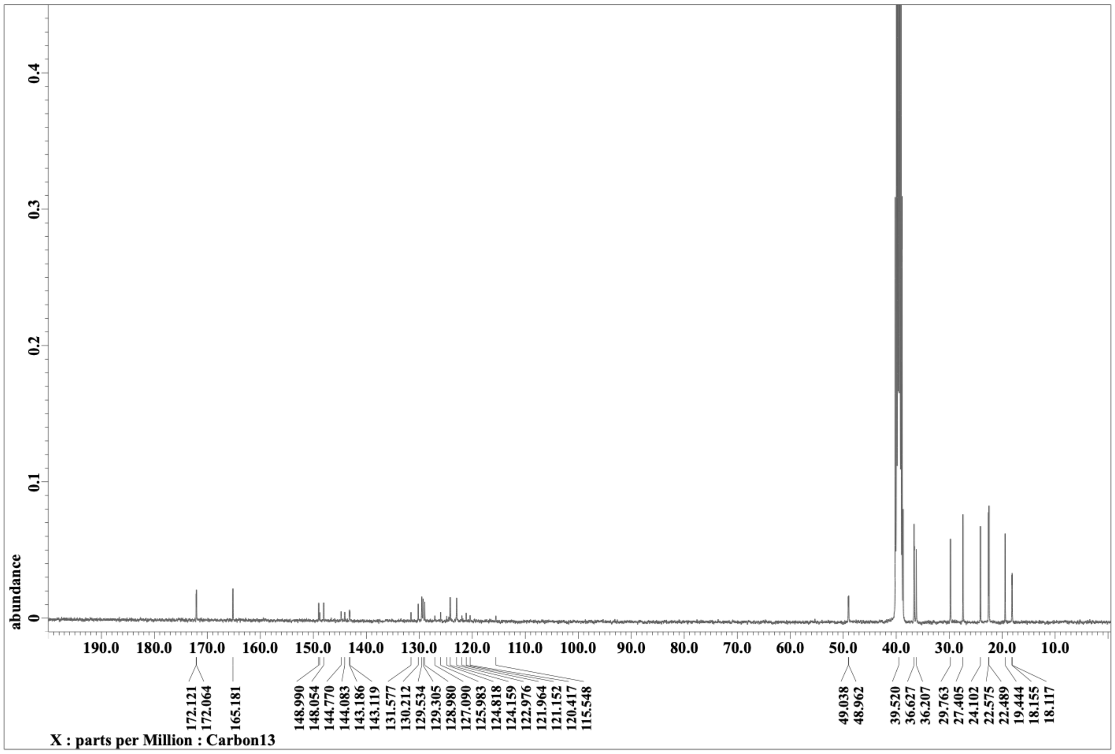

Supplementary Fig. 37. ^{13}C NMR spectrum of compound **1** (100 MHz, 298 K, DMSO-d_6).

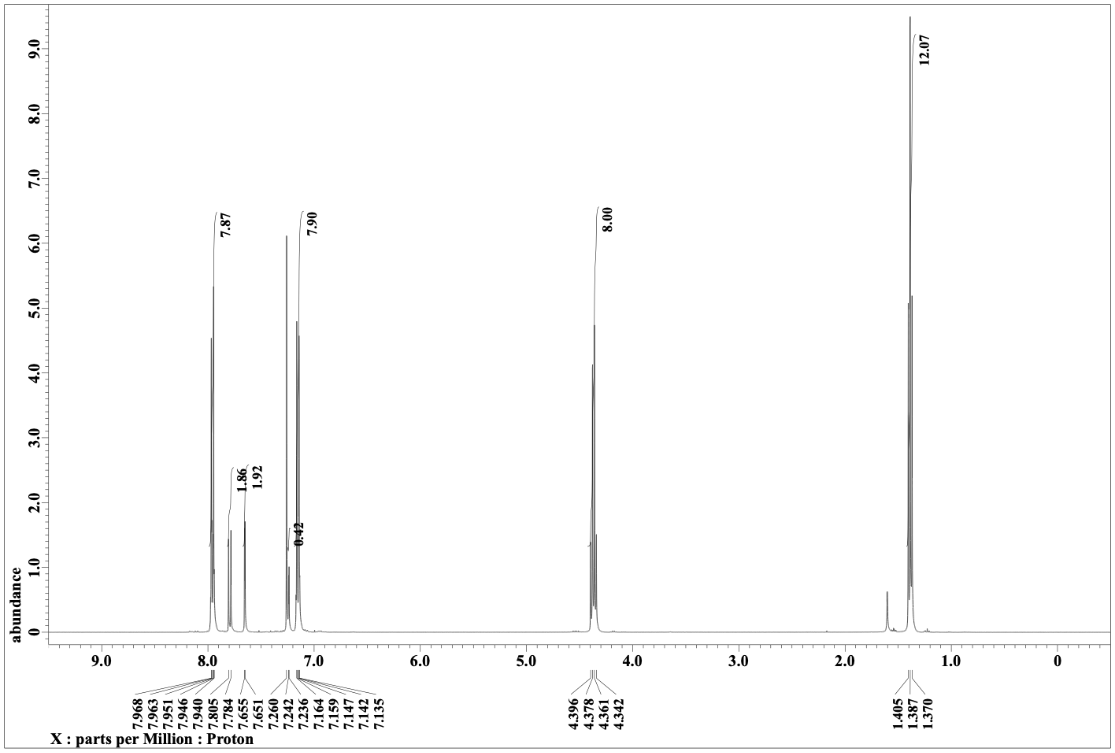

Supplementary Fig. 38. ^{1}H NMR spectrum of compound **S10** (400 MHz, 298 K, $CDCl_3$).

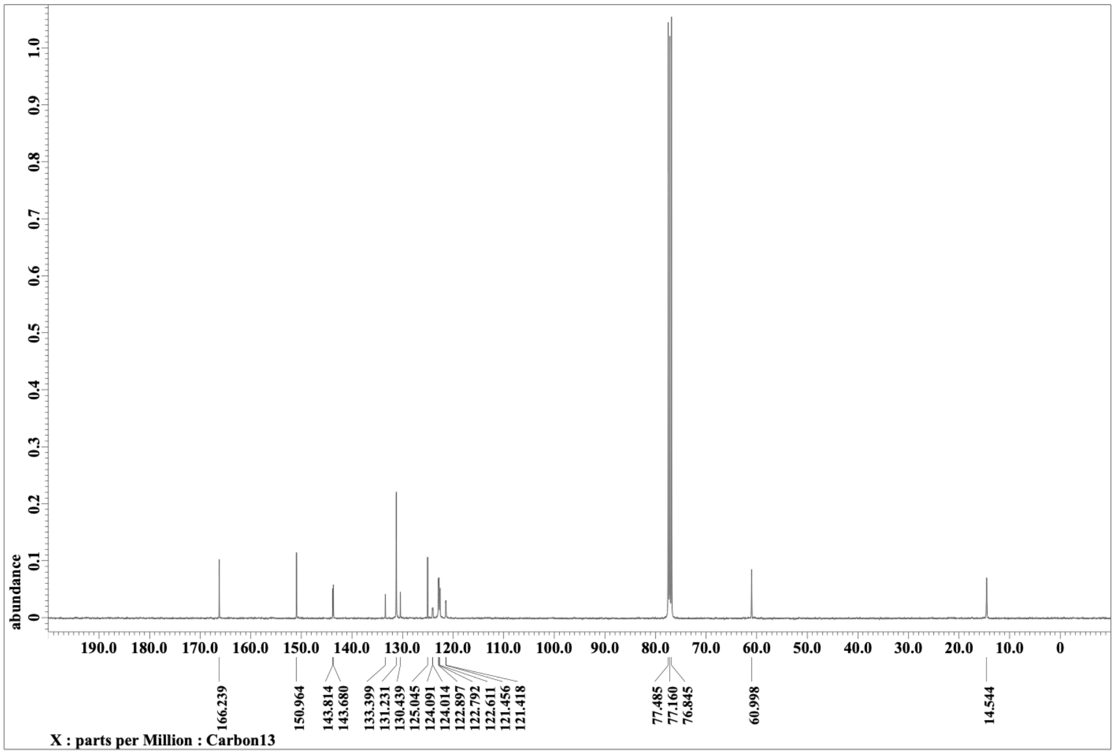

Supplementary Fig. 39. ^{13}C NMR spectrum of compound **S10** (100 MHz, 298 K, $CDCl_3$).

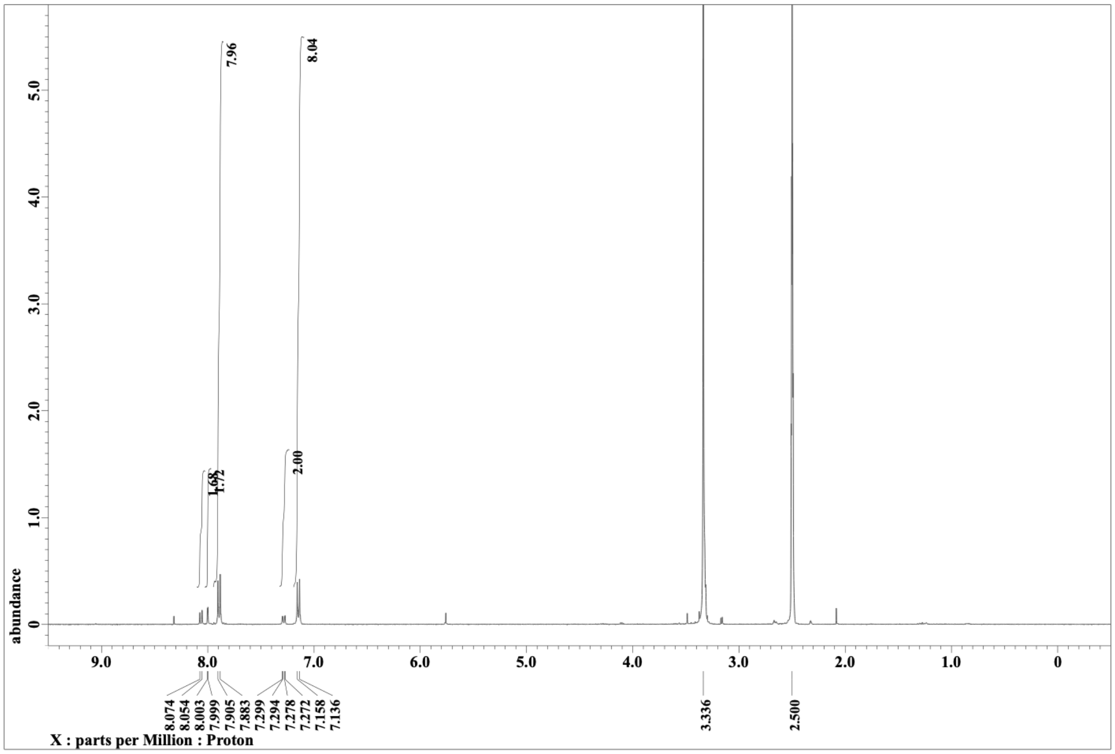

Supplementary Fig. 40. ^{1}H NMR spectrum of compound **S11** (400 MHz, 298 K, DMSO-d_6).

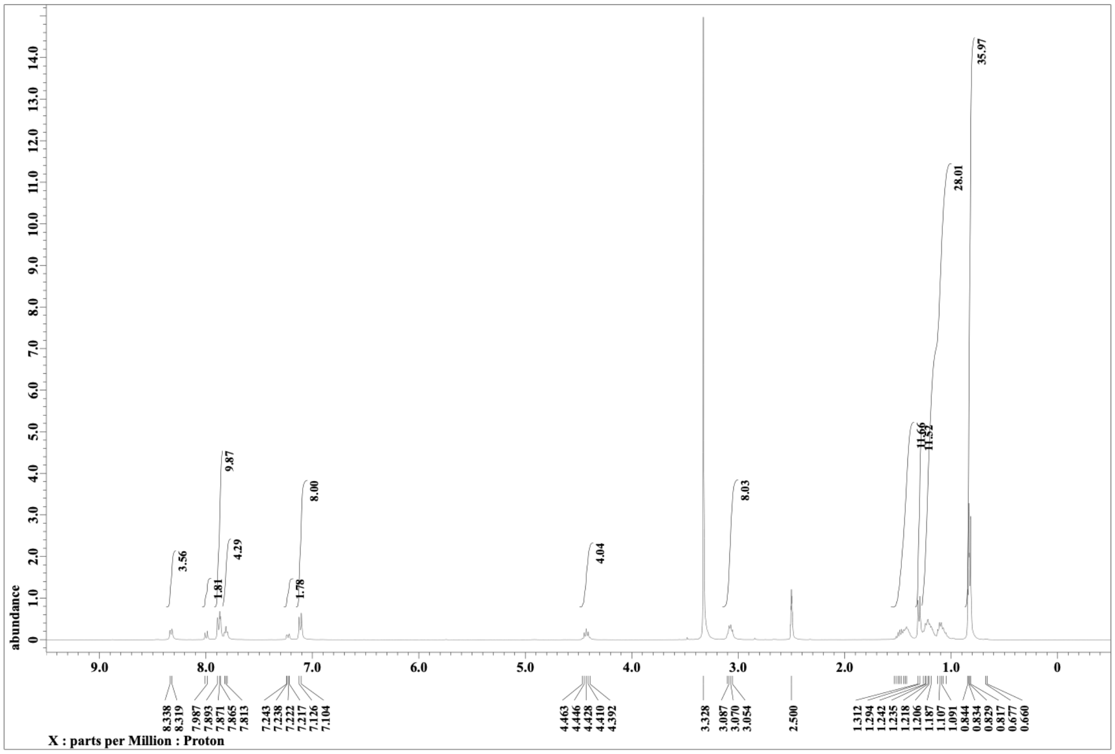

Supplementary Fig. 41. ^{1}H NMR spectrum of compound **2** (400 MHz, 298 K, DMSO-d_6).

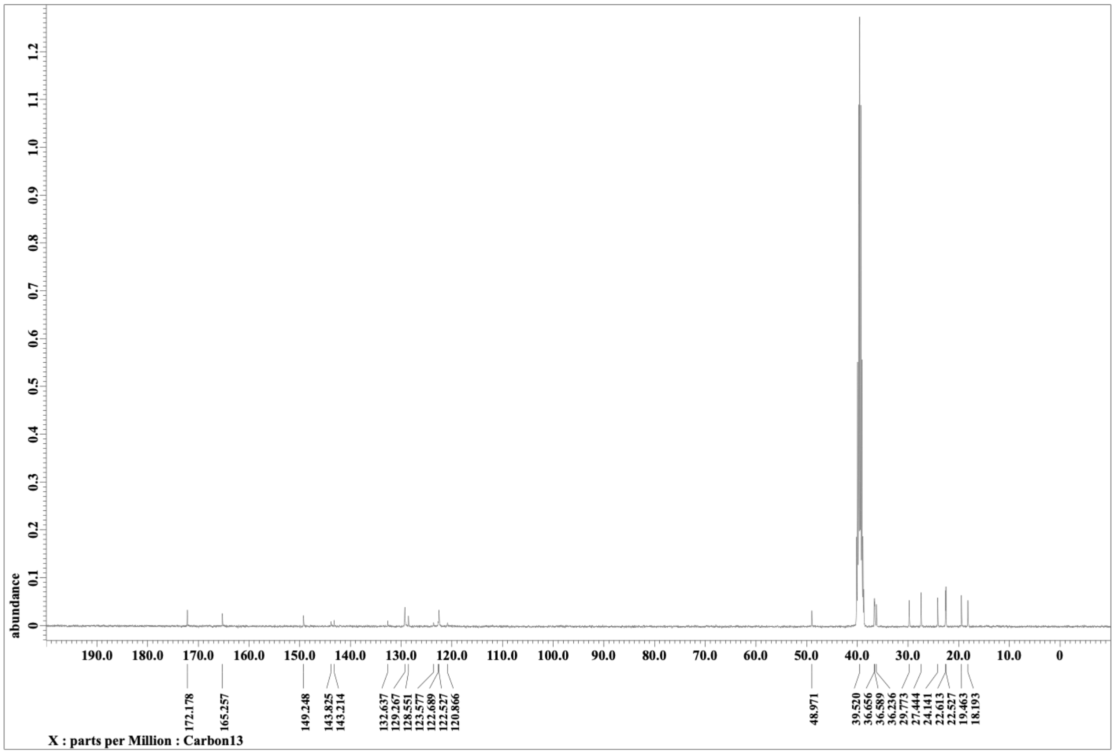

Supplementary Fig. 42. ^{13}C NMR spectrum of compound **2** (100 MHz, 298 K, DMSO-d_6).

References

[1] Terashima, T. et al. Single-chain folding of polymers for catalytic systems in water. *J. Am. Chem. Soc.* **133**, 4742–4745 (2011).

[2] Saito, M. et al. One-step synthesis of [1]benzothieno[3,2-*b*][1]benzothiophene from *o*-chlorobenzaldehyde. *Tetrahedron Lett.* **52**, 285–288 (2011).

[3] Sanzone, A. et al. Efficient synthesis of organic semiconductors by Suzuki–Miyaura coupling in an aromatic micellar medium. *Green Chem.* **21**, 4400–4405 (2019).

[4] Taki, M., Kajiwara, K., Yamaguchi, E., Sato, Y. & Yamaguchi, S. Fused thiophene-*S*,*S*-dioxide-based super-photostable fluorescent marker for lipid droplets. *ACS Materials Lett.* **3**, 42–49 (2021).

[5] de Carvalho. J. G. M., Geißer, K., Weishäupl, S. J., Fischer, R. A. & Pöthig, A. Alkaline earth metal–organic frameworks based on tetratopic anthraquinone-based linkers: synthesis, characterization, and photochemical applications. *Inorg. Chem.* **61**, 15831–15840 (2022).

[6] Takimiya, K. et al. 2,7-Diphenyl[1]benzothieno[3,2-*b*]benzothiophene, a new organic semiconductor for air-stable organic field-effect transistors with mobilities up to 2.0 $cm^2\ V^{-1}\ s^{-1}$. *J. Am. Chem. Soc.* **128**, 12604–12605 (2006).

[7] Zherdeva, S. Y., Barudi, A., Zheltov, A. & Stepanov, B. Synthesis and reactions of 2,7-disubstituted benzothieno[3,2-*b*]benzothiophenes. *Zh. Org. Khim.* **16**, 430–438 (1980).

[8] Korevaar, P. A., Schaefer, C., de Greef, T. F. A. & Meijer, E. W. Controlling chemical self-assembly by solvent-dependent dynamics. *J. Am. Chem. Soc.* **134**, 13482–13491 (2012).
