# Supplementary material for: Programmable Construction of Supramolecular Polymers Achieved in Neutral Lipid Environments
Source: Nat Commun. 2026 Jul 6;17:5732. doi: 10.1038/s41467-026-74683-9 (PMC13338038; doi:10.1038/s41467-026-74683-9)
Supplement: Supplementary file 2 — Transparent Peer Review file [file 41467_2026_74683_MOESM2_ESM.pdf]

nature portfolio

Peer Review File

Programmable Construction of Supramolecular Polymers Achieved in Neutral Lipid Environments

Corresponding Author: Dr Soichiro Ogi

This file contains all reviewer reports in order by version, followed by all author rebuttals in order by version.

Version 0:

Reviewer comments:

Reviewer #1

(Remarks to the Author)
This manuscript deals with solvent effect on supramolecular polymerization. Amide functionalized pi-conjugated monomer undergoes supramolecular polymerization via nucleation-elongation mechanism, an extensively studied research area in the past 7-8 years. Now they use triolein (TO) as a solvent, which has comparable dielectric constant with di-n-butyl ether (DBE). In both solvents, comparable supramolecular polymerization features were noted except that in the m-values in TO was higher than that in DBE, indicating that TO offers an enhanced dynamic range for modulating the polymer-monomer equilibria. First of all this is no surprise, considering vast structural difference between these two solvents. Solvent structures are already known to greatly influence the supramolecular polymerization in many different ways. Therefore minor difference observed between TO and DBE is unlikely to be relevant to the broad readership. Secondly, it has been emphasized that TO environment mimics Lipid droplets (LDs), and the results shown here may be relevant to understanding supramolecular polymerization in LDs. Not sure yet (unless this is done), whether this study provides any distinct clue(that was not known before by studying supramolecular polymerization in hydrocarbons or other low polarity solvents) that will be specifically be relevant for studying supramolecular polymerization in LD ! Moreover, what is the potential impact (either in advancing knowledge or applications) of studying supramolecular polymerization in LDs is not clear to this reviewer. Thirdly, seeded supramolecular polymerization, multi block copolymers etc. are known in many different other systems, this paper does not bring any conceptually new aspects.

Overall the manuscript lacks scientific merit, novelty and impact for publication in Nature Communication. Rather trivial and highly system- specific (lacking general applicability) issues have been projected in an articulate manner. The manuscript is not recommended for publication in Nature Communication.

Reviewer #2

(Remarks to the Author)
In the manuscript titled ‘Programmable Construction of Supramolecular Polymers Achieved in Neutral Lipid Environments’, Naruse, Yamaguchi and co-workers report on two types of supramolecular monomers that polymerize in apolar media to form various types of supramolecular polymers. Specifically, the authors find that the kinetics of the nucleated supramolecular polymerizations can be controlled through the choice of solvent: polymerizations in triolein occur significantly slower than in ethyl oleate. The authors show they can exploit this difference in kinetics to make various supramolecular copolymer architectures by sequential addition of monomers.

This paper’s main finding, that the solvents induce distinctly different dynamics in the seeded supramolecular polymerizations, and can be used to control the copolymer morphology, is an interesting finding. In itself, the various aspects, such as living supramolecular polymerizations and kinetic control over these polymerizations, are not novel. See for instance the work of Sugiyasu, Meijer, Würthner, and others. In that sense, the current manuscript provides further proof and corroboration of prior findings, which would better suit a more specialized chemistry journal.
However, the system Naruse and Yamaguchi report does offer an opportunity to investigate the solvent dependency of living supramolecular polymerization further. More detailed investigations into this topic in a revised manuscript would warrant publication in Nature Communications. Hence, I recommend to publish the manuscript elsewhere in the current form.

I have the following remarks and suggestions if the authors wish to revise their manuscript.
1. I fail to see the relevance of the introduction to the rest of the paper. The introduction introduces lipid droplets, compartmentalized systems, and dynamics of apolar solvents. In their manuscript, the authors discuss various supramolecular polymerizations, and note that ‘delayed nucleation … cannot be fully explained by viscosity’, strongly suggesting that monomer properties and solvation, rather than lipid dynamics are important driving factors in the polymerization.
2. The authors refer to several examples of supramolecular polymerizations in living cells. I would argue these examples (from 2007) are not particularly recent.
3. I appreciate the differences in viscosities between the various solvents, but this research question could be better introduced. If viscosity is indeed a main property of the solvent that is investigated, it would be interesting to see how the polymerization occurs in some other solvents with similar polarity, but different viscosity.
4. Lines 160-170, the authors show that the different solvents induce different kinetics in the polymerization. More detailed investigations into how these kinetics depend on the solvent ratios will be of value. Does the lag phase increase linearly, or are there non-linear effects? Can this dependency be described by some kinetic model? Is it possible to describe a linear free energy relationship in terms of not only the thermodynamic driving forces, but also the activation barriers?
5. The authors report quite different viscosities compared to literature reports (see, e.g., 10.1016/j.molliq.2024.124525). Can the authors comment on this?
6. The authors seem to have normalized the spectroscopic data at each concentration to [0, 1] before fitting the mass balance model to the data. This implies that the system is fully polymerized at the lowest propanol percentages. While this appears reasonable for some titrations (Fig S7d), other titrations have not plateaued yet (Fig 2e), suggesting there is still a significant amount of free monomers left at low n-propanol percentages. The authors should take this into account in their modelling.
7. Lines 223-230: The authors argue that 1 in the copolymers is more stable than in the homopolymers, due to stabilization by the energetically more stable 2. Do the authors have further evidence for this? And how do the authors asses the relative stability of 1-2 contacts with respect to 1-1 contacts and 2-2 contacts?
8. Line 285-286, do the authors make the claim for bundling based on the differences observed in CLSM (Fig 2c) and TEM images (Fig S4)? A sentence on this would be informative.
9. I commend the authors on otherwise diligent experimenting and fitting of their data to obtain quantitative descriptions, both thermodynamically and kinetlcally, of the system.
9. Formatting: I would recommend to label the right panels in figure 3b and c separately. These portray different experiments than the middle and left panels.

Reviewer #3

(Remarks to the Author)
In this manuscript, Naruse, Ogi, and Yamaguchi report an intriguing controlled supramolecular polymerization using triolein – a natural triacylglycerol lipid. The authors employed two types of fluorophore monomers and conducted a detailed kinetic study using both spectroscopic and microscopic techniques, focusing on how the triolein medium affects the kinetic pathway of cooperative supramolecular polymerization. They found that triolein suppresses spontaneous nucleation and further prevents the bundling of the resulting supramolecular polymers, with di-n-butyl ether and ethyl oleate used as key reference solvents. This finding was successfully applied to the creation of multiblock copolymers by combining it with a seed polymerization method. In the final part of the manuscript, the authors provide theoretical insight into the observed phenomena by analyzing how the medium stabilizes both monomeric and aggregated states, and they introduce m-values for a quantitative discussion. The scientific rationale and experimental design are rigorous and of high quality. However, some results remain unclear due to insufficient explanation. Therefore, I recommend that the manuscript be considered for publication in Nature Communications after minor revision, provided that the following specific points are appropriately addressed.

1. First, the authors state that triolein (TO) is characterized by its hydrogen bond–acceptor ability. However, throughout the manuscript, it remains unclear what specific types of interactions contribute to the stabilization of the monomeric and aggregated states. If possible, the authors should clarify these points, and revise certain sentences to make the underlying interactions more explicit. For example, in lines 156-158, “chemical compatibility between solvent and monomer, rather than viscosity, plays a dominant role in determining the kinetics of self-assembly” and line 167, “likely due to the presence of cis-9-octadecenyl chains, which may inhibit the formation of higher-order structures”. In the last part, in line 289, “exposing the diamide groups to the solvent and enabling more efficient depolymerization“ and in lines 304-305, “three ester groups and cis-9-octadecenyl chains to suppress spontaneous nucleation of the solutes and prevent bundling of supramolecular polymers”.

2. Related to point #1, the description in the text does not fully correspond to the cartoon shown in Fig. 1b, which may cause confusion for readers. Specifically, what does the “1Agg” state represent? In the main text, from line 89, the authors primarily discuss the “1AggS” state. Are these two terms referring to the same molecular state? If so, clarification is necessary.
In a similar vein, the schematic illustration in the middle of Fig. 3a does not seem to match the data shown in the corresponding middle panel of Fig. 3b (red circles), which suggests that no aggregation occurs in triolein. The inconsistency between these illustrations and the data should be addressed to improve clarity and coherence.

3. Similarly, it is unclear what the authors specifically mean by the term “higher-order structure.” The manuscript lacks a detailed explanation or definition of this concept. Clarification on what structural features are being referred to would greatly improve the reader’s understanding.

4. The term lipid droplet (LD) typically evokes structured assemblies such as micelles or bilayers. Do triolein (TO) and ethyl oleate (EO) exhibit any capacity to form such weakly structured solvent environments? If so, could a cage effect also contribute to the observed delay in nucleation or the spontaneous formation of seeded aggregates?

5. In this study, 1-propanol is used as a good solvent for the monomers, and all experiments are conducted in mixed solvent systems containing TO or EO. Is there any information regarding the homogeneity of these mixed solvents, particularly with respect to potential local phase separation? If such separation occurs, it would be reasonable to assume that the solvation environments of the monomer and aggregate could differ significantly. The authors are encouraged to provide their perspective on this point.

Version 1:

Reviewer comments:

Reviewer #2

(Remarks to the Author)
Naruse, Yamaguchi and co-workers provide an updated and revised version of their manuscript. The authors have expanded their existing experimental dataset primarily with further spectroscopic investigations to underscore their findings on the influence of fatty acid-based solvents on supramolecular polymerizations. In my earlier report, I wrote that in my opinion, the manuscript provides further proof and corroboration for earlier reports, while also providing an opportunity to investigate solvent effects in more detail and obtain new mechanistic understanding.
The manuscript provides a strong collection of experimental findings, but despite their further clarifications on their experimental data, the current manuscript does not shed conceptually new light on the role of solvents in supramolecular polymerizations. In that sense, I agree with reviewer 1, and think that this manuscript is interesting to a selected audience, rather than Nature Communication’s broad public. Hence, I remain with my recommendation to publish the manuscript elsewhere.

Below are some further comments for the authors that I hope will be helpful in revising their manuscript:
- New insights that extend beyond what can readily be obtained with the conventional suite of spectroscopies and microscopies would give interesting new insights into novel aspects or generalities of the solvent-mediated supramolecular polymerizations.
- The IR results point to hydrogen bonding of the solvent with the monomers, which is very interesting. Additional work in this direction could shed light on interesting properties of the system. How are these hydrogen bonds organized? Are they strong, weak, dynamic, rigid? Do MD simulations provide clues? Or time-resolved spectroscopies? Are these principles general, and if so, how can one use them to engineer specific properties of the system in a targeted way?
- As for the fitting, the authors could give some further details. The SI of the reference they cite for fitting does actually report normalization of the data. Did the authors do this too, or not? These details remain unclear.

Reviewer #3

(Remarks to the Author)
The authors have fully responded to all the comments raised by this reviewer, as well as those from the other reviewers, in a detailed and satisfactory manner. In addition, they have carried out a series of new experiments, including nucleation studies in mixed solvents (with varying compositions), temperature-dependent UV measurements, and fluorescence microscopy, all of which have contributed to improving and clarifying the content of the paper. I sincerely appreciate the authors’ careful revisions. In my opinion, all the points raised by the reviewers have been clearly addressed, and the revised manuscript is now suitable for publication in Nature Communications.
I would like to congratulate the authors on their conceptually insightful and well-executed work.

Open Access This Peer Review File is licensed under a Creative Commons Attribution 4.0 International License, which permits use, sharing, adaptation, distribution and reproduction in any medium or format, as long as you give appropriate credit to the original author(s) and the source, provide a link to the Creative Commons license, and indicate if changes were made.
In cases where reviewers are anonymous, credit should be given to 'Anonymous Referee' and the source.
The images or other third party material in this Peer Review File are included in the article's Creative Commons license, unless indicated otherwise in a credit line to the material. If material is not included in the article's Creative Commons license and your intended use is not permitted by statutory regulation or exceeds the permitted use, you will need to obtain permission directly from the copyright holder.
To view a copy of this license, visit https://creativecommons.org/licenses/by/4.0/

[What has been revised in this manuscript]
Our reply to the Reviewer's comments and what has been revised are listed as follows.

Response to the comments from Reviewer 1:

Reviewer #1: This manuscript deals with solvent effect on supramolecular polymerization. Amide functionalized pi-conjugated monomer undergoes supramolecular polymerization via nucleation-elongation mechanism, an extensively studied research area in the past 7-8 years. Now they use triolein (TO) as a solvent, which has comparable dielectric constant with di-n-butyl ether (DBE). In both solvents, comparable supramolecular polymerization features were noted except that in the m-values in TO was higher than that in DBE, indicating that TO offers an enhanced dynamic range for modulating the polymer-monomer equilibria.

First of all this is no surprise, considering vast structural difference between these two solvents. Solvent structures are already known to greatly influence the supramolecular polymerization in many different ways. Therefore minor difference observed between TO and DBE is unlikely to be relevant to the broad readership.

Re: We thank the reviewer for this thoughtful and frank assessment. We fully agree that supramolecular polymerization is a well-established field and that solvent structure has long been recognized as an important factor governing assembly behavior. Our aim in this study, however, was not to suggest that any difference between the solvents examined here should in itself be regarded as unexpected. Rather, we wished to show that a neutral-lipid medium, triolein (TO), influences not only nucleation but also the subsequent structural evolution of the assemblies in a manner that cannot be explained simply in terms of bulk polarity or viscosity.

In the revised manuscript, we have reinforced this point by including additional experiments using ethyl oleate (EO) and mixed-solvent systems (Supplementary Figs. S9, S10, and S13 in the revised Supporting Information; see the response to comments 3 and 4 of Reviewer 2). These results show that the lag time and kinetic stability do not change in a straightforward manner with bulk solvent parameters, but instead exhibit a nonlinear dependence on solvent composition. In combination with the FT-IR results indicating carbonyl–diamide interactions (Supplementary Fig. S11 in the revised Supporting Information), these observations suggest that delayed nucleation and suppression of inter-fiber bundling arise from both specific solvent–monomer interactions and the hydrocarbon-rich environment characteristic of neutral lipids.

For this reason, we believe that the significance of the present study does not lie in a simple comparison between TO and DBE as individual solvents. Rather, it lies in showing that neutral lipids can play a distinct role in directing supramolecular polymerization through specific solvation effects and through their influence on fiber growth and bundling after nucleation. To make this point clearer, we have revised the Introduction (pages 2–3), the Results subsection entitled "Effects of triolein on assembly kinetics" (pages 8–9), and the Discussion (pages 14–15) in the revised manuscript.

Secondly, it has been emphasized that TO environment mimics Lipid droplets (LDs), and the results shown here may be relevant to understanding supramolecular polymerization in LDs. Not sure yet (unless this is done), whether this study provides any distinct clue(that was not known before by studying supramolecular polymerization in hydrocarbons or other low polarity solvents) that will be specifically be relevant for studying supramolecular polymerization in

LD ! Moreover, what is the potential impact (either in advancing knowledge or applications) of studying supramolecular polymerization in LDs is not clear to this reviewer.

Re: We appreciate the reviewer's concern regarding the relevance of this study to lipid droplets (LDs). In the revised manuscript, we have clarified that our focus is on bulk neutral-lipid media as model environments relevant to LDs, rather than on LDs as complete cellular organelles. We believe that this approach is valuable for two reasons. First, the present study shows that a neutral-lipid-rich medium such as TO does not simply behave as another low-polarity solvent, but can influence supramolecular polymerization through a combination of specific carbonyl-based solvation and a hydrocarbon-rich environment, leading to delayed nucleation and suppression of inter-fiber bundling. This provides insight that is not readily obtained from studies using conventional hydrocarbon or ether solvents alone and offers a basis for designing molecular systems intended to operate in LD-related environments. Second, it offers a physicochemical perspective for future studies aimed at reading out or modulating LD-associated processes with self-assembling π-conjugated systems, an area that remains much less explored than imaging-based approaches.

To avoid overstating the biological implications, we have revised the Introduction and Discussion so that the manuscript now presents TO as a representative neutral-lipid medium and treats the connection to LDs more cautiously, as part of the motivation and a possible future direction of this work, rather than as a direct reproduction of native LD behavior.

On page 2 of the revised manuscript, in the first paragraph of the Introduction: *This prompted us to explore whether neutral-lipid media, which serve as simplified model environments relevant to LDs, might offer an opportunity not only to study supramolecular assembly under LD-related conditions but also to examine how such media regulate assembly pathways.*

On page 16 of the revised manuscript, in the last sentence of the Discussion: *Given that TO is a representative triacylglycerol present in LDs, these results also provide a basis for future studies aimed at designing self-assembling molecular systems that operate in LD-relevant environments.*

Thirdly, seeded supramolecular polymerization, multi block copolymers etc. are known in many different other systems, this paper does not bring any conceptually new aspects.

Re: We agree with the reviewer that seeded supramolecular polymerization and multiblock copolymer formation are already well-established concepts in this field. We therefore do not claim that these phenomena are, in themselves, conceptually new. Rather, the contribution of the present study lies in showing that a neutral-lipid medium can create conditions under which spontaneous nucleation is suppressed while seeded growth remains effective, thereby allowing supramolecular copolymers to be constructed in a pathway-selective manner under conditions where uncontrolled aggregation would otherwise compete.

From this perspective, the multiblock structures presented here are not meant to serve as an independent claim of novelty, but rather as a functional demonstration of the solvent effect revealed in this study. We have revised the manuscript accordingly to make this point clearer and to avoid overstating the novelty of seeded polymerization itself.

On page 14 of the revised manuscript, the end of 1st paragraph: *These results underscore the utility of TO as a medium whose kinetic influence on supramolecular polymerization enables sequential seeded growth in a controlled manner, leading to pathway-selective construction of multiblock supramolecular polymer architectures.*

On page 15 of the revised manuscript, in the last paragraph of the Discussion: *We propose that TO suppresses spontaneous nucleation by stabilizing the monomeric state through hydrogen bonding between its ester carbonyl groups and the diamide moieties of **1**, while its cis-9-octadecenyl-chain-rich hydrocarbon environment stabilizes the dispersed supramolecular polymer state by suppressing van der Waals-driven inter-fiber bundling. This enables precise kinetic control over supramolecular polymer growth, as confirmed by kinetic studies showing delayed nucleation, stabilization of the dispersed aggregated state, and exceptional responsiveness to seeded growth (red filled circles in Fig. 3h). Similar behavior was observed in EO, further supporting the view that neutral lipids can influence both thermodynamic and kinetic aspects of supramolecular polymerization in ways not captured by conventional low-polarity solvents alone. Importantly, we applied these characteristics to achieve stepwise seeded growth of supramolecular multiblock nanostructures in TO. These findings identify low-polarity neutral lipids as useful media for directing pathway-selective supramolecular polymerization.*

Overall the manuscript lacks scientific merit, novelty and impact for publication in Nature Communication. Rather trivial and highly system specific (lacking general applicability) issues have been projected in an articulate manner. The manuscript is not recommended for publication in Nature Communication.

Re: We appreciate the reviewer's concerns regarding novelty and broader impact. In revising the manuscript, we have sought to clarify that the central contribution of this work is not the observation of living supramolecular polymerization in a different solvent, but the identification of a solvent effect specific to neutral-lipid media, one that influences nucleation, suppresses inter-fiber bundling, and creates conditions that enable pathway-selective supramolecular polymerization. We have also strengthened the basis for this conclusion by adding solvent-composition studies, comparison experiments with EO, microscopy data, and a more explicit discussion of specific solvent–monomer interactions.

We hope that these revisions help to convey the broader significance of the present study more clearly: namely, that neutral lipids can serve as chemically distinctive media for controlling supramolecular assembly and may provide a useful basis for designing supramolecular systems intended to function in LD-relevant low-polarity environments.

Reviewer #2: In the manuscript titled 'Programmable Construction of Supramolecular Polymers Achieved in Neutral Lipid Environments', Naruse, Yamaguchi and co-workers report on two types of supramolecular monomers that polymerize in apolar media to form various types of supramolecular polymers. Specifically, the authors find that the kinetics of the nucleated supramolecular polymerizations can be controlled through the choice of solvent: polymerizations in triolein occur significantly slower than in ethyl oleate. The authors show they can exploit this difference in kinetics to make various supramolecular copolymer architectures by sequential addition of monomers.

This paper's main finding, that the solvents induce distinctly different dynamics in the seeded supramolecular polymerizations, and can be used to control the copolymer morphology, is an interesting finding. In itself, the various aspects, such as living supramolecular polymerizations and kinetic control over these polymerizations, are not novel. See for instance the work of Sugiyasu, Meijer, Würthner, and others. In that sense, the current manuscript provides further proof and corroboration of prior findings, which would better suit a more specialized chemistry journal. However, the system Naruse and Yamaguchi report does offer an opportunity to investigate the solvent dependency of living supramolecular polymerization further. More detailed investigations into this topic in a revised manuscript would warrant publication in Nature Communications. Hence, I recommend to publish the manuscript elsewhere in the current form.
I have the following remarks and suggestions if the authors wish to revise their manuscript.

Re: We sincerely thank the reviewer for the careful assessment of our manuscript and for the opportunity to revise it. In response to the reviewer's constructive remarks and suggestions, we have substantially revised the manuscript as detailed below:

1. I fail to see the relevance of the introduction to the rest of the paper. The introduction introduces lipid droplets, compartmentalized systems, and dynamics of apolar solvents. In their manuscript, the authors discuss various supramolecular polymerizations, and note that 'delayed nucleation … cannot be fully explained by viscosity', strongly suggesting that monomer properties and solvation, rather than lipid dynamics are important driving factors in the polymerization.

Re: The previous Introduction could be read as placing undue emphasis on "lipid-droplet (LD) dynamics" relative to the present focus on supramolecular polymerization in triolein (TO). In the revised manuscript, we therefore revised the first paragraph to more clearly articulate the significance of using TO as a neutral-lipid medium. In addition, to avoid any conflation with solvent "dynamics", we replaced the term "LD dynamics" with the more specific expression "LD behavior and interaction with other organelles". We also strengthened the connection between the Introduction and the results by highlighting monomer properties and solvation, and by refocusing the mechanistic motivation on solvent compatibility and specific intermolecular interactions as key factors governing nucleation and the formation of higher-order aggregates. This revision is consistent with our conclusion that the delayed nucleation observed in TO cannot be explained by viscosity alone.

On page 2 of the revised manuscript, 1st paragraph: *Lipid droplets (LDs) are cellular organelles composed mainly of neutral lipids, such as triacylglycerols and cholesterol esters, and play essential roles in cellular functions through regulated lipid storage and interactions with other organelles.*[1–3] *Dysregulation of lipid storage and inter-organelle communication has been linked to metabolic and proliferative diseases, including obesity, diabetes, and cancer.*[4–8] *Consequently, increasing attention has been directed toward understanding LD behavior in cells, propelled by the development of LD-specific fluorescent probes*[9–19] *and advanced fluorescence imaging techniques.*[20–22] *Despite this progress in visualization, molecular strategies that can operate in LD-relevant environments and actively manipulate LD-associated processes remain limited.*

On page 2 of the revised manuscript, the end of 1st paragraph: *This prompted us to explore whether neutral-lipid media, which serve as simplified model environments relevant to LDs, might offer an opportunity not only to study supramolecular assembly under LD-related conditions but also to examine how such media regulate assembly pathways.*

On page 2 of the revised manuscript, 2nd paragraph: *To harness the functional potential of low-polarity neutral lipids in supramolecular polymerization, we focused on their role in controlling nucleation, fiber growth, and inter-fiber bundling through solvent-solute interactions. Previous studies have demonstrated that solvent compatibility critically guides supramolecular building blocks into well-defined helical or hierarchical architectures under thermodynamic control.*[35–45] *Solvent composition can likewise exert a pronounced effect on kinetically controlled assembly pathways.*[46,47] *However, these insights have largely been established in conventional organic solvents, and it remains unclear whether neutral-lipid media can exert assembly-regulating effects beyond those expected from bulk polarity alone. Because the early stages of aggregation are often governed by subtle solvation differences and specific intermolecular interactions, bulk properties such as polarity or viscosity may not fully capture solvent behavior. On this basis, we identified triolein (TO), a representative triacylglycerol, as an LD-relevant model medium for testing this idea.*

2. The authors refer to several examples of supramolecular polymerizations in living cells. I would argue these examples (from 2007) are not particularly recent.

Re: We acknowledge that the previous text overstated the recency of this area. We have therefore replaced the statement suggesting a "recent" emergence so that it more accurately reflects the development of this field over the past two decades. In addition, the former review references are now cited as Refs. 33a,b, and three recent representative reports from 2024 and 2025 have been added as Refs. 34a–c to highlight that this field continues to advance.

On page 2 of the revised manuscript, 1st paragraph: *In this context, supramolecular polymerization within living cells has developed over the past two decades as a promising strategy for controlling cellular functions.*[23–34]

Reference: *[33] (a) Chagri, S., Ng, D. Y. W. & Weil, T. Designing bioresponsive nanomaterials for intracellular self-assembly. Nat. Rev. Chem.* ***6****, 320–338 (2022); (b) Kim, J. et al. In situ self-assembly for cancer therapy and imaging. Nat. Rev. Mater.* ***8****, 710–725 (2023).*
[34] (a) Ren, Y. et al. Supramolecular assembly in live cells mapped by real-time phasor-fluorescence lifetime imaging. J. Am. Chem. Soc. ***146****, 11991–11999 (2024); (b) Ren, Y. et al. Intracellular assembly of supramolecular peptide nanostructures controlled by visible light. Nat.*

Synth. ***4***, *673–683 (2025); (c) Wang, H. et al. Synthesis of abiotic supramolecular polymers inside living cells via organocatalysis-mediated self-assembly. Angew. Chem. Int. Ed.* ***64***, *e202500998 (2025).*

3. I appreciate the differences in viscosities between the various solvents, but this research question could be better introduced. If viscosity is indeed a main property of the solvent that is investigated, it would be interesting to see how the polymerization occurs in some other solvents with similar polarity, but different viscosity.

Re: We appreciate the reviewer's suggestion that the research question should be introduced more clearly. Our intention was not to identify viscosity as the primary solvent parameter, but rather to examine whether the delayed nucleation observed in neutral lipid media can be explained by a simple bulk property alone.

In response to this comment, we performed additional experiments using both DBE/EO and EO/TO mixed solvents to further assess the origin of the nucleation delay. EO was selected as an intermediate solvent because it has a polarity similar to that of TO (ε = 3.17 at T = 28 °C for EO; ε = 3.20 at T = 25 °C for TO), while remaining substantially less viscous (7.01 $mm^2 \cdot s^{-1}$ for EO; 44.7 $mm^2 \cdot s^{-1}$ for TO). In DBE/EO mixtures, the lag time changes nonlinearly and increases sharply in EO-rich regimes (Fig. R1). Moreover, a lag time comparable to that in pure TO was still observed even when 50% EO was added to TO (Fig. R2). These results suggest that solvent-specific solvation and solvent–monomer compatibility, rather than viscosity alone, play key roles in determining the nucleation kinetics.

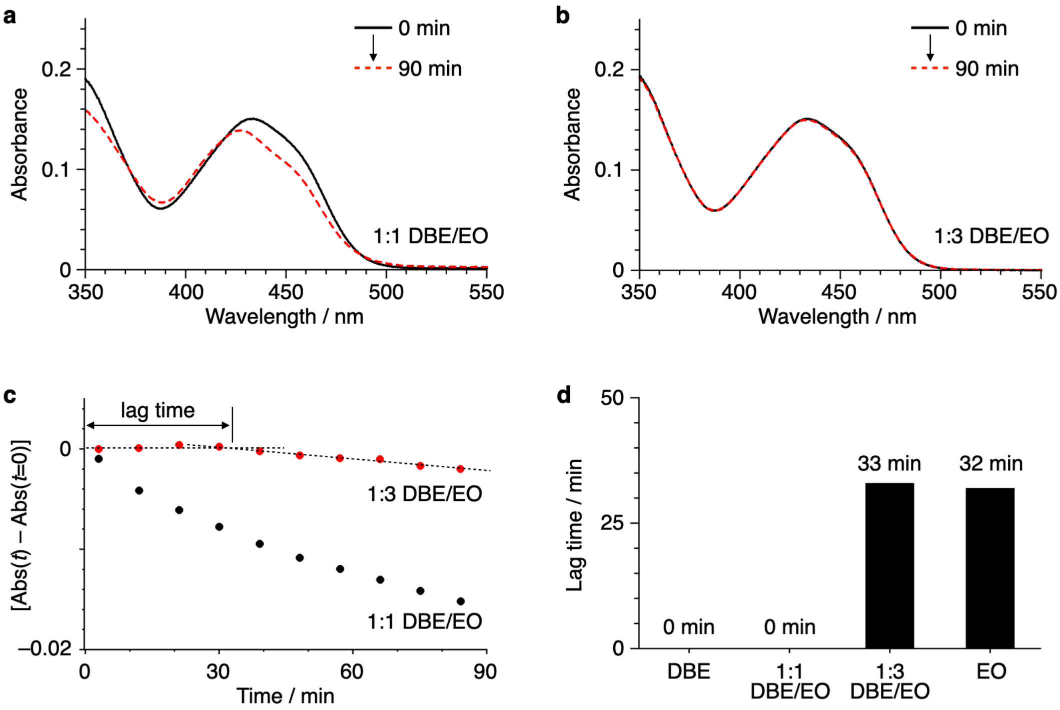

Fig. R1 a,b Time-dependent UV–vis absorption spectra of $\mathbf{1}_{\mathbf{Mono}}$ in 1:1 DBE/EO (**a**) and 1:3 DBE/EO (**b**). **c** Time-dependent changes in absorbance at 433 nm for $\mathbf{1}_{\mathbf{Mono}}$ in 1:1 DBE/EO (black) and 1:3 DBE/EO (red), each containing 3 vol% 1-propanol, at c_T = 1.0 × 10^{-5} M and T = 293 K. **d** Comparison of lag times before nucleation.

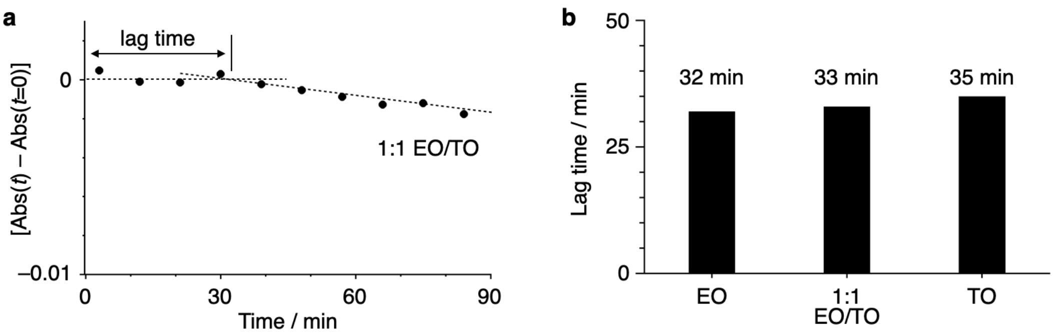

Fig. R2 a time-dependent changes in absorbance at 433 nm for **1$_{Mono}$** in 1:1 EO/TO containing 3 vol% of 1-propanol, at c_T = 1.0 × 10^{-5} M and T = 293 K. **b** Comparison of lag times before nucleation.

These results are included as Supplementary Figures S9 and S10 in the revised supporting information. We also revised the Introduction and the relevant Results/Discussion text to clarify from the outset that viscosity is treated as a reference bulk property, whereas the central mechanistic focus of this study is on solvation and specific solvent–monomer interactions.

On page 2 of the revised manuscript: *This combination of hydrogen-bond-accepting carbonyl groups and a hydrocarbon-rich environment is expected to influence nucleation and bundling behavior in a manner not accessible in conventional low-polarity solvents.*

On page 8 of the revised manuscript: *To examine whether this kinetic difference can be explained by a simple bulk solvent property, we investigated EO, a solvent bearing a long alkyl chain and a carbonyl group. In this solvent, we again observed a measurable lag phase (~30 min) before the onset of self-assembly, similar to that seen in TO (Fig. 3c; Supplementary Fig. S8c,d). This behavior is inconsistent with a simple viscosity-based explanation, because TO is considerably more viscous (44.7 mm^2·s^{-1}) than EO (7.01 mm^2·s^{-1}). To further clarify the origin of this delay, we performed additional experiments using mixed solvents. In DBE/EO mixtures, the lag time changed nonlinearly and increased sharply in EO-rich regimes (Supplementary Fig. S9). Moreover, in EO/TO mixtures, a lag time comparable to that in pure TO was retained even when TO was diluted with 50% EO (Supplementary Fig. S10). These nonlinear composition dependences are difficult to reconcile with viscosity alone and instead point to a major contribution from specific solvent-monomer interactions.*

4. Lines 160−170, the authors show that the different solvents induce different kinetics in the polymerization. More detailed investigations into how these kinetics depend on the solvent ratios will be of value. Does the lag phase increase linearly, or are there non−linear effects? Can this dependency be described by some kinetic model? Is it possible to describe a linear free energy relationship in terms of not only the thermodynamic driving forces, but also the activation barriers?

Re: We would first like to clarify that Lines 160–170 in the original manuscript discuss the solvent-dependent kinetic stability of the sonication-prepared aggregate **1AggS**, rather than the kinetics of supramolecular polymerization from the monomeric state. To address the reviewer's question regarding solvent-ratio dependence, we added composition-dependent measurements for DBE/EO mixtures. These experiments showed that the kinetic stability of **1AggS** does not change linearly with solvent

composition; rather, the lag time increases sharply upon initial addition of EO to DBE, whereas the change becomes much smaller between 1:1 DBE/EO and pure EO (Fig. R3). This result indicates a non-linear dependence of the kinetic behavior on solvent ratio. We also examined EO/TO mixtures and found that the TO effect remains significant even at low TO fractions (3:1 EO/TO shown in Figure R3), further supporting the importance of solvent-specific interactions.

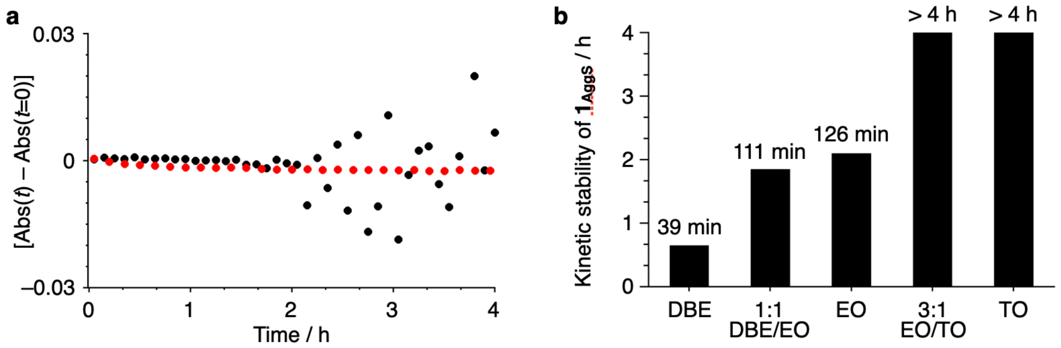

Fig. R3 a Time-dependent changes in absorbance at 433 nm for $\mathbf{1}_{\mathbf{AggS}}$ in EO (black) and in 3:1 EO/TO (red), each containing 3 vol% of 1-propanol, at $c_T = 1.0 \times 10^{-5}$ M and T = 293 K. **b** Comparison of the kinetic stability of $\mathbf{1}_{\mathbf{AggS}}$.

These additional results are now included in the revised Supporting Information as Figure S13, and the relevant discussion has been added to clarify the solvent-composition dependence.

On page 9 of the revised manuscript: *A similarly high kinetic stability was also observed in pure EO, indicating that this stabilizing effect persists in EO-rich media (Supplementary Fig. S13). In addition, in EO/TO mixtures, a lag time longer than 4 h was retained even when only 25 vol% TO was added to EO (Supplementary Fig. S13). These results suggest the presence of solvent-specific interactions that strongly influence the kinetic stability of* $\mathbf{1}_{\mathbf{AggS}}$*. This behavior is likely related to the cis-9-octadecenyl-chain-rich hydrocarbon environment in TO and EO, which suppresses inter-fiber association and thereby inhibits the formation of bundled structures.*

Regarding the reviewer's question about activation barriers, a meaningful evaluation would require temperature-dependent rate constants for the relevant bundling-related process. To examine this point, we performed temperature-dependent UV–vis measurements on **1AggS**. However, upon heating, the aggregate dispersion became rapidly unstable, showing a sudden increase in absorbance and unstable spectral shapes at higher temperatures (Fig. R4). Because this behavior does not provide a well-defined homogeneous kinetic process, reliable rate constants for bundling could not be obtained.

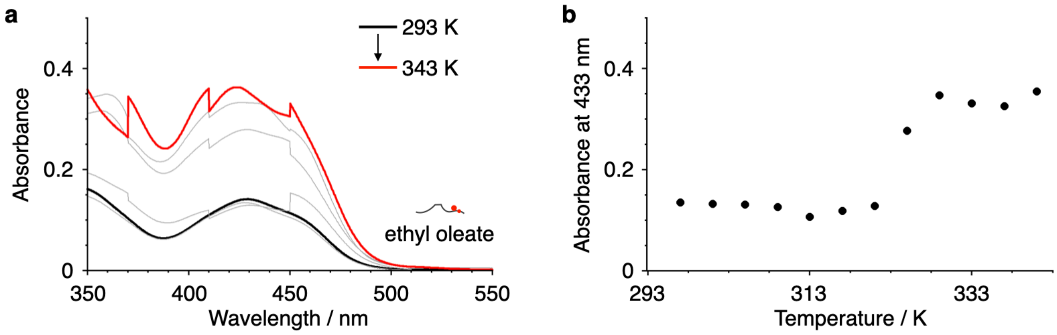

Fig. R4 a Temperature-dependent UV-vis absorption spectra of $\mathbf{1}_{\mathbf{Aggs}}$ in ethyl oleate containing 3 vol% of 1-propanol at c_T = 1.0 × 10^{-5} M upon heating from 293 K to 343 K at 1 K/min. **b** Temperature-dependent changes in the absorbance at 433 nm.

5. The authors report quite different viscosities compared to literature reports (see, e.g., 10.1016/j.molliq.2024.124525). Can the authors comment on this?

Re: On the apparent discrepancy with the literature, we clarified that our manuscript reports kinematic viscosity (ν, $mm^2 \cdot s^{-1}$) measured experimentally using an Ubbelohde-type viscometer (TN-102-05 and TN-102-07, Takao Manufacturing Co., Ltd.) at room temperature, whereas the shared literature reports dynamic viscosity (η, mPa·s) with density (ρ, g/cm^3) at 293 K. Using the relation $\nu = \eta/\rho$, the literature values for EO (η = 5.81 mPa·s, ρ = 0.8701 g/cm^3) correspond to ν = 6.68 $mm^2 \cdot s^{-1}$, which is close to our measured value (7.01 $mm^2 \cdot s^{-1}$). The remaining difference may reasonably arise from differences in temperature and measurement method. We have therefore revised the Methods to define the viscosity type and units clearly.

On page 16 of the revised manuscript, Characterization: *The kinematic viscosities of triolein and ethyl oleate were measured at ambient temperature using an Ubbelohde-type viscometer (TN-102-05 and TN-102-07, Takao Manufacturing Co., Ltd.) and are reported in units of* $mm^2\ s^{-1}$.

6. The authors seem to have normalized the spectroscopic data at each concentration to [0, 1] before fitting the mass balance model to the data. This implies that the system is fully polymerized at the lowest propanol percentages. While this appears reasonable for some titrations (Fig S7d), other titrations have not plateaued yet (Fig 2e), suggesting there is still a significant amount of free monomers left at low n-propanol percentages. The authors should take this into account in their modelling.

Re: The raw experimental data were directly used in the fitting procedure, without prior normalization to [0, 1]. Therefore, the model does not assume complete polymerization at the initial 3 vol% 1-propanol condition. As shown in Fig. 2e, the degree of aggregation (α_{agg}) remains below 1 even at this initial condition, indicating that the system is not yet fully shifted to the aggregated state. Thus, the remaining fraction of free monomer is already considered in the modeling. We acknowledge that the original caption of Fig. 2e may have suggested a prior normalization step. We have therefore revised the caption to clarify that the fitting was based directly on the raw absorbance data.

On page 7 of the revised manuscript, the caption Fig. 2: *Aggregation parameter (α_{agg}) calculated from the raw absorbance data at λ_{abs} = 433 nm based on the global-fit result of the solvent-dependent cooperative model, plotted against the volume fraction of 1-propanol.*

7. Lines 223–230: The authors argue that 1 in the copolymers is more stable than in the homopolymers, due to stabilization by the energetically more stable 2. Do the authors have further evidence for this? And how do the authors asses the relative stability of 1–2 contacts with respect to 1–1 contacts and 2–2 contacts?

Re: Our original description may have conveyed an overly strong implication regarding the origin of the apparent stabilization of the $\mathbf{1}_{\mathbf{Agg}}$ domain in the copolymer. Our intention was not to suggest particularly **1–2** contacts, as pointed out by the reviewer, but rather to describe the observed behavior as being consistent with a block-like supramolecular architecture. To make this point clearer, we have adjusted the text so that it now states only that, under the denaturation conditions employed, the $\mathbf{1}_{\mathbf{Agg}}$ domain in the copolymer appears more persistent than in the corresponding **1** homopolymer. We further note that this behavior is consistent with the behavior reported for block supramolecular copolymers formed in low-polarity solvents through intermolecular hydrogen-bonding interactions, in which disassembly proceeds preferentially from the polymer termini, resulting in greater apparent persistence of the central block (*J. Am. Chem. Soc.* **2018**, *140*, 10570–10577). In this way, the discussion is framed in terms of structural and kinetic effects, without implying direct energetic stabilization. Accordingly, the relevant passage has been revised to better reflect our intended meaning and to avoid overstatement.

On page 12 of the revised manuscript, 2nd paragraph: *Compared to the denaturation behavior of **1** homopolymers (Fig. 2e), the domain of the aggregated **1** in the copolymer appeared more persistent upon addition of 1-propanol. A related kinetic effect has been reported for block supramolecular polymers formed in low-polarity media through intermolecular hydrogen-bonding interactions, in which disassembly proceeds preferentially from the polymer termini, leading to enhanced apparent persistence of the central block.[53] In the present system, we therefore interpret this behavior in terms of structural and kinetic effects.*

Reference: *[53] Jung, S. H., Bochicchio, D., Pavan, G. M., Takeuchi, M. & Sugiyasu, K. A block supramolecular polymer and its kinetically enhanced stability. J. Am. Chem. Soc. 140, 10570–10577 (2018).*

8. Line 285–286, do the authors make the claim for bundling based on the differences observed in CLSM (Fig 2c) and TEM images (Fig S4)? A sentence on this would be informative.

Re: The statement in Lines 285–286 was originally based on the time-dependent changes in absorbance shown by the black filled circles in Fig. 3b (middle) and Supplementary Fig. S10b, from which we inferred that $\mathbf{1}_{\mathbf{AggS}}$ tends to undergo bundling more readily in DBE. By contrast, the CLSM image in Fig. 2c shows 1AggS in TO, and the TEM image in Supplementary Fig. S4a shows $\mathbf{1}_{\mathbf{AggS}}$ in DBE, but both were obtained immediately after sonication and therefore do not directly capture the subsequent structural evolution associated with bundling.

To address this point more directly, we additionally examined $\mathbf{1}_{\mathbf{AggS}}$ in both DBE and TO after standing for several hours. In DBE, a more bundled morphology was observed after 120 min (Fig. R5a,b), indicating progressive higher-order aggregation over time. In

contrast, in TO, similar fragmented fibrous aggregates were observed even after 4 h (Fig. R5c,d).

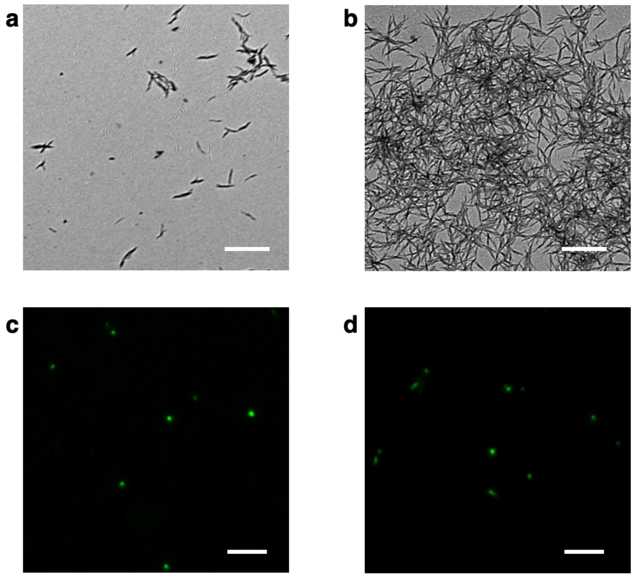

Fig. R5 a,b TEM images of (**a**) $\mathbf{1}_{\mathbf{Aggs}}$ formed in DBE at c_T = 1.0 × 10^{-5} M just after sonication and (**b**) bundled aggregates obtained after 120 min; scale bar: 2 μm. **c,d** CLSM images of $\mathbf{1}_{\mathbf{Aggs}}$ in TO at c_T = 1.0 × 10^{-5} M obtained just after sonication (**c**) and after 4 h (**d**); λ_{ex} = 405 nm; λ_{em} = 490–590 nm; scale bar: 5 μm.

These observations support the conclusion that the stability and structural evolution of $\mathbf{1}_{\mathbf{Aggs}}$ differ markedly depending on the solvent. These additional results are now included in the revised Supporting Information as Fig. S24, and we have added a clarifying sentence in the revised manuscript to reflect this point.

On page 15 of the revised manuscript, 1st paragraph: *In DBE, supramolecular polymers tend to evolve into more bundled aggregates upon standing, as suggested by the absorption changes (black filled circles in Fig. 3e; Supplementary Fig. S12b) and by TEM observations showing a more bundled morphology after standing of* $\mathbf{1}_{\mathbf{Aggs}}$ *for 120 min (Supplementary Fig. S24a,b). Such bundling likely shields the hydrogen-bonding diamide moieties and thereby dampens the response to 1-propanol. In contrast, TO facilitates the formation of well-dispersed supramolecular polymers, as supported by CLSM observations showing similarly fragmented fibrous aggregates even after 4 h (Fig. 2 and Supplementary Fig. S24c,d).*

9. I commend the authors on otherwise diligent experimenting and fitting of their data to obtain quantitative descriptions, both thermodynamically and kinetically, of the system.

Re: We sincerely thank the reviewer for this encouraging comment and for appreciating our efforts in the quantitative thermodynamic and kinetic analysis of the system.

10. Formatting: I would recommend to label the right panels in figure 3b and c separately. These portray different experiments than the middle and left panels.

Re: We have revised Fig. 3 by labeling all panels to clarify that each panel corresponds to a distinct experiment or data set and to improve the overall readability of the figure.

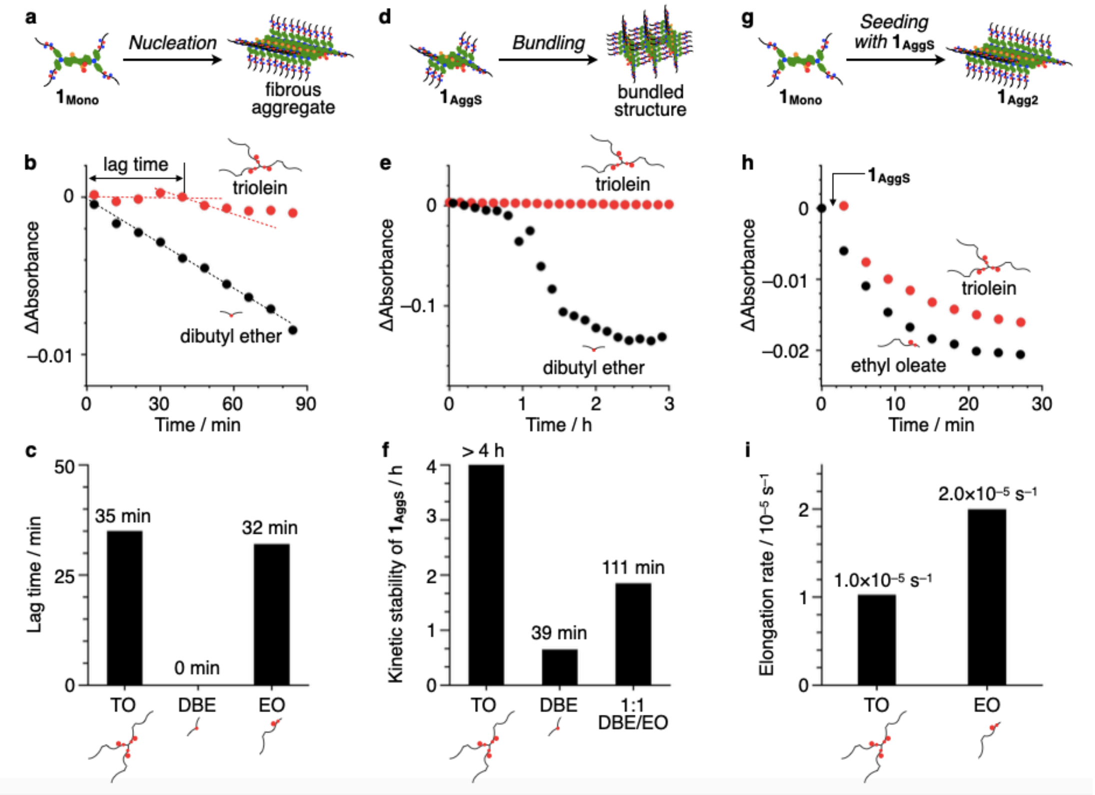

Fig. 3 Kinetic studies of the self-assembly behavior of 1 in triolein and related solvents. a,d,g General schematic illustrations of the processes examined in this figure: spontaneous nucleation of $\mathbf{1}_{\mathbf{Mono}}$ (**a**), bundling of $\mathbf{1}_{\mathbf{AggS}}$ (**d**), and seed-initiated assembly of **1** (**g**). **b,e,h** Time-dependent changes in absorbance at 433 nm for $\mathbf{1}_{\mathbf{Mono}}$ (**b**) and $\mathbf{1}_{\mathbf{AggS}}$ (**e**) in 97:3 TO/1-propanol ($c_T = 1.0 \times 10^{-5}$ M; red filled circles) and in 97:3 DBE/1-propanol ($c_T = 1.0 \times 10^{-5}$ M; black filled circles), and before and after addition of a solution of $\mathbf{1}_{\mathbf{AggS}}$ ($c_T = 1.0 \times 10^{-5}$ M; 0.12 mL) to a fresh solution of $\mathbf{1}_{\mathbf{Mono}}$ ($c_T = 1.0 \times 10^{-5}$ M; 1.2 mL) in 97:3 TO/1-propanol (red) and in EO/1-propanol (black) at $T = 293$ K (**h**). **c,f,i** Comparison of the lag time before nucleation (**c**), the kinetic stability of $\mathbf{1}_{\mathbf{AggS}}$ (**f**), and the elongation rate determined at $\lambda_{abs} = 433$ nm in each solvent containing 3 vol% of 1-propanol (**i**).

Reviewer #3: In this manuscript, Naruse, Ogi, and Yamaguchi report an intriguing controlled supramolecular polymerization using triolein – a natural triacylglycerol lipid. The authors employed two types of fluorophore monomers and conducted a detailed kinetic study using both spectroscopic and microscopic techniques, focusing on how the triolein medium affects the kinetic pathway of cooperative supramolecular polymerization. They found that triolein suppresses spontaneous nucleation and further prevents the bundling of the resulting supramolecular polymers, with di-n-butyl ether and ethyl oleate used as key reference solvents. This finding was successfully applied to the creation of multiblock copolymers by combining it with a seed polymerization method. In the final part of the manuscript, the authors provide theoretical insight into the observed phenomena by analyzing how the medium stabilizes both monomeric and aggregated states, and they introduce m-values for a quantitative discussion. The scientific rationale and experimental design are rigorous and of high quality. However, some results remain unclear due to insufficient explanation. Therefore, I recommend that the manuscript be considered for publication in Nature Communications after minor revision, provided that the following specific points are appropriately addressed.

Re: We greatly appreciate the kind words of support for our manuscript. To improve the quality of the manuscript we have addressed the constructive comments by the Reviewer in the following ways:

1. First, the authors state that triolein (TO) is characterized by its hydrogen bond-acceptor ability. However, throughout the manuscript, it remains unclear what specific types of interactions contribute to the stabilization of the monomeric and aggregated states. If possible, the authors should clarify these points, and revise certain sentences to make the underlying interactions more explicit. For example, in lines 156-158, "chemical compatibility between solvent and monomer, rather than viscosity, plays a dominant role in determining the kinetics of self-assembly" and line 167, "likely due to the presence of cis-9-octadecenyl chains, which may inhibit the formation of higher-order structures". In the last part, in line 289, "exposing the diamide groups to the solvent and enabling more efficient depolymerization" and in lines 304-305, "three ester groups and cis-9-octadecenyl chains to suppress spontaneous nucleation of the solutes and prevent bundling of supramolecular polymers".

Re: We agree with the reviewer that the specific interactions responsible for kinetic stabilization were not described clearly enough in the original manuscript, and we have revised the relevant text to make these points explicit.

For the monomeric state, our interpretation is that kinetic stabilization is promoted by hydrogen-bonding interactions between the carbonyl groups of the medium and the diamide moiety of monomeric **1**. This assignment is supported by the FT-IR result already shown in the original submission (Supplementary Fig. S9), which indicates that the carbonyl group of ethyl oleate (EO) interacts with the diamide unit of **1**. We now state this more clearly in the main text and clarify that the "chemical compatibility" term refers, in this context, to specific solvent–solute interactions involving carbonyl-containing media.

For the aggregated state, we agree that the wording was too vague. Because the supramolecular polymer is formed through intermolecular hydrogen bonding between diamide groups and π–π interactions between the aromatic cores, the aggregate surface is expected to be covered predominantly by branched alkyl chains. We therefore interpret the suppressed higher-order bundling in TO as arising from the hydrocarbon-rich

environment provided by the cis-9-octadecenyl chains, which likely reduces effective inter-fiber van der Waals-driven association (i.e., bundling) between supramolecular polymers. We have revised the relevant sentences to distinguish this effect from the carbonyl-mediated stabilization of the monomeric state.

Accordingly, we revised the cited sentences (former lines 156–158, 167, 289, and 304–305) to describe (i) carbonyl–diamide interactions as a factor in monomer stabilization and (ii) suppression of inter-fiber bundling in a cis-9-octadecenyl-chain-rich medium as a factor in aggregate kinetic persistence.

On page 8 of the revised manuscript: *These results suggest that specific solvent–monomer interactions, particularly hydrogen bonding between the carbonyl groups of the neutral lipids and the diamide moiety of **1**, play a more dominant role than viscosity in determining the kinetics of self-assembly.*

On page 9 of the revised manuscript: *This behavior is likely related to the cis-9-octadecenyl-chain-rich hydrocarbon environment in TO and EO, which suppresses inter-fiber association and thereby inhibits the formation of bundled structures.*

On page 15 of the revised manuscript: *This dispersed morphology, likely stabilized by van der Waals interactions with the three cis-9-octadecenyl chains, exposes the diamide groups to 1-propanol and enables more efficient depolymerization.*

On page 15 of the revised manuscript: *We propose that TO suppresses spontaneous nucleation by stabilizing the monomeric state through hydrogen bonding between its ester carbonyl groups and the diamide moieties of **1**, while its cis-9-octadecenyl-chain-rich hydrocarbon environment stabilizes the dispersed supramolecular polymer state by suppressing van der Waals-driven inter-fiber bundling.*

2. Related to point #1, the description in the text does not fully correspond to the cartoon shown in Fig. 1b, which may cause confusion for readers. Specifically, what does the “1Agg” state represent? In the main text, from line 89, the authors primarily discuss the “1AggS” state. Are these two terms referring to the same molecular state? If so, clarification is necessary. In a similar vein, the schematic illustration in the middle of Fig. 3a does not seem to match the data shown in the corresponding middle panel of Fig. 3b (red circles), which suggests that no aggregation occurs in triolein. The inconsistency between these illustrations and the data should be addressed to improve clarity and coherence.

Re: The terminology for the aggregated **1** and schematic illustrations in Fig. 3a were not sufficiently consistent in the original manuscript, and we appreciate the reviewer for pointing this out.

First, regarding the terminology for the homopolymers of **1**, we have revised the manuscript to use only two terms: $\mathbf{1}_{\mathbf{AggS}}$, which denotes the aggregates prepared by sonication, and $\mathbf{1}_{\mathbf{AggL}}$, which denotes the longer aggregates obtained through seeded polymerization of **1**. This revision was made to avoid ambiguity and to ensure that the terminology directly reflects the experimentally defined aggregate states. In addition, the notation $\mathbf{1}_{\mathbf{Agg}}$ is now used only in the expression $\mathbf{2}_{\mathbf{Agg}}$-$\mathbf{1}_{\mathbf{Agg}}$-$\mathbf{2}_{\mathbf{Agg}}$, where it refers specifically to the aggregated **1** domain within the block structure formed with **2**. We have added this clarification in the main text so that the terminology is used consistently throughout the manuscript and does not mislead readers.

On page 5 of the revised manuscript, 2nd paragraph: *This spectral change was accompanied by the emergence of positive circular dichroism (CD) signals at 362 nm and 406 nm and a negative signal at 466 nm (Fig. 2b), indicating aggregate formation with chiral excitonic coupling between chromophores. Notably, the resulting aggregates retained moderate fluorescence (green dashed line in Fig. 2a), which enabled visualization of green-emissive nanostructures with a size of less than 1 µm in TO using fluorescence imaging (Fig. 2c). Hereafter, this sonication-generated aggregate state is referred to as* $\mathbf{1_{AggS}}$.

On page 11 of the revised manuscript, 1st paragraph: *We further validated the feasibility of a sequential seeded growth process by introducing the elongated aggregates with a length of around 3 µm, hereafter denoted as* $\mathbf{1_{AggL}}$ *to distinguish them from the sonication-prepared* $\mathbf{1_{AggS}}$ *(Supplementary Fig. S15c,d), into a fresh solution of* $\mathbf{1_{Mono}}$ *in TO/1-propanol (97:3, v/v) at a 30:1 volume ratio of* $\mathbf{1_{Mono}}$ *to* $\mathbf{1_{AggL}}$.

On page 14 of the revised manuscript, 2nd paragraph: *The merged CLSM image confirmed that aggregated **2** domains grew from both ends of aggregated **1**, resulting in the formation of a triblock copolymer,* $\mathbf{2_{Agg}}$-$\mathbf{1_{Agg}}$-$\mathbf{2_{Agg}}$ *(Fig. 4e).*

Second, we thank the reviewer for pointing out the potential confusion regarding the schematic in the middle of Fig. 3a. We agree that its relationship to the corresponding data in the middle panel of Fig. 3b (red circles) was not sufficiently clear in the original manuscript. The schematic was not intended to represent the specific behavior observed in triolein; rather, it was designed as a general illustration of the bundling process examined in the TO, DBE, and 1:1 DBE/EO systems shown in the middle panel of Fig. 3b. To clarify this point, we have revised the relevant figure caption and the corresponding description in the main text so that the schematic is clearly presented as a conceptual representation of the monitored process, rather than as a direct depiction of the experimental outcome in any particular solvent. In addition, to further improve clarity and facilitate correspondence between the text, figure, and caption, we have labeled all panels in Fig. 3 as a–i (see the response to comment 10 of Reviewer 2).

On page 8 of the revised manuscript, 2nd paragraph: *We then turned to the kinetic stability of the sonication-generated aggregate* $\mathbf{1_{AggS}}$ *before its evolution into bundled structures, as evaluated by time-dependent UV–vis absorption spectroscopy and fluorescence imaging (Fig. 3d).*

On page 9 of the revised manuscript, caption of Fig. 3: ***Fig. 3 Kinetic studies of the self-assembly behavior of 1 in triolein and related solvents. a,d,g*** *General schematic illustrations of the processes examined in this figure: spontaneous nucleation of* $\mathbf{1_{Mono}}$ *(**a**), bundling of* $\mathbf{1_{AggS}}$ *(**d**), and seed-initiated assembly of **1** (**g**). **b,e,h** Time-dependent changes in absorbance at 433 nm for* $\mathbf{1_{Mono}}$ *(**b**) and* $\mathbf{1_{AggS}}$ *(**e**) in 97:3 TO/1-propanol (*$c_T = 1.0 \times 10^{-5}$ *M; red filled circles) and in 97:3 DBE/1-propanol (*$c_T = 1.0 \times 10^{-5}$ *M; black filled circles), and before and after addition of a solution of* $\mathbf{1_{AggS}}$ *(*$c_T = 1.0 \times 10^{-5}$ *M; 0.12 mL) to a fresh solution of* $\mathbf{1_{Mono}}$ *(*$c_T = 1.0 \times 10^{-5}$ *M; 1.2 mL) in 97:3 TO/1-propanol (red) and in EO/1-propanol (black) at* $T = 293$ *K (**h**). **c,f,i** Comparison of the lag time before nucleation (**c**), the kinetic stability of* $\mathbf{1_{AggS}}$ *(**f**), and the elongation rate determined at* $\lambda_{abs} = 433$ *nm in each solvent containing 3 vol% of 1-propanol (**i**).*

3. Similarly, it is unclear what the authors specifically mean by the term "higher-order structure." The manuscript lacks a detailed explanation or definition of this concept.

Clarification on what structural features are being referred to would greatly improve the reader's understanding.

Re: We thank the reviewer for pointing out this lack of clarity. Upon revisiting the manuscript, we found that the terms "higher-order aggregates", "higher-order morphology", and "higher-order structures" were used only in the Abstract and Introduction, whereas the Results section consistently referred to the corresponding states as "bundled structures", "bundled aggregates", or "bundled morphology." We agree that this inconsistency in terminology could obscure the structural meaning intended in the earlier sections.

In this study, the structural change of interest is the inter-fiber association of supramolecular polymers into bundled and eventually precipitating assemblies, rather than a distinct hierarchical level defined separately from bundling. To avoid ambiguity, we have therefore revised the Abstract and Introduction to replace the "higher-order …" expressions with terminology consistent with the Results section, namely "bundled structures", "inter-fiber bundling", or equivalent descriptions as appropriate.

On page 1 of the revised manuscript, Abstract: *However, triolein significantly suppresses the spontaneous formation of supramolecular polymers and bundled aggregates, thereby creating a lag phase during which seeded polymerization can guide monomers along a defined assembly pathway.*

On page 2 of the revised manuscript, 2nd paragraph: *To harness the functional potential of low-polarity neutral lipids in supramolecular polymerization, we focused on their role in controlling nucleation, fiber growth, and inter-fiber bundling through solvent-solute interactions.*

On page 3 of the revised manuscript, 2nd paragraph: *Further investigation using ethyl oleate (EO; ε = 3.17 at T = 28 °C), a reference solvent bearing a partial structure of TO, demonstrated that TO delays spontaneous nucleation and promote dispersion of the fibrous assemblies, thereby preventing inter-fiber bundling into larger aggregated structures.*

4. The term lipid droplet (LD) typically evokes structured assemblies such as micelles or bilayers. Do triolein (TO) and ethyl oleate (EO) exhibit any capacity to form such weakly structured solvent environments? If so, could a cage effect also contribute to the observed delay in nucleation or the spontaneous formation of seeded aggregates?

Re: Triolein (TO) is known to be dispersible in aqueous surfactant solutions as surfactant-stabilized TO droplets (Shimobayashi and Ohsaki, *PNAS* **2019**, *116*, 25440–25445). In addition, we recently reported self-assembly behavior inside such TO droplets (*Small* **2025**, e13411). In that study, we showed that a supramolecular building block, constructed by introducing two phenylalanine-based diamide units into a fluorescent molecule previously reported as an LD probe, undergoes spontaneous self-assembly within surfactant-stabilized TO droplets. Furthermore, that study also revealed that the lag time before the onset of self-assembly and the morphology of the resulting aggregates were similar in bulk triolein solution and inside the droplets. These findings suggest that, at least in that system, the confined environment of TO droplets does not drastically alter the self-assembly behavior, but instead largely reflects the self-assembly characteristics observed in bulk TO media.

5. In this study, 1–propanol is used as a good solvent for the monomers, and all experiments are conducted in mixed solvent systems containing TO or EO. Is there any information regarding the homogeneity of these mixed solvents, particularly with respect to potential local phase separation? If such separation occurs, it would be reasonable to assume that the solvation environments of the monomer and aggregate could differ significantly. The authors are encouraged to provide their perspective on this point.

Re: In mixed solvent systems containing 1-propanol and neutral lipids (TO or EO), local inhomogeneity could influence the effective solvation environments of monomeric and aggregated species. To examine this possibility, we performed additional fluorescence microscopy observations of the TO/1-propanol mixed system (97 vol% TO, with **1** initially dissolved in 1-propanol and then thoroughly mixed). Under these conditions, we did not observe any micrometer-scale droplet-like phase separation (Fig. R6). This result suggests that resolvable microscale phase separation is absent under our experimental conditions, at least in the TO-containing system. We therefore revised the manuscript to clarify that our discussion is based on the absence of observable microscale phase separation, while possible nanoscale inhomogeneity remains an important subject for future study.

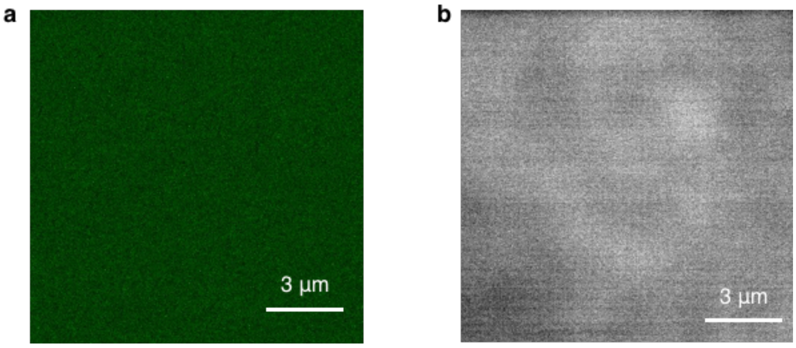

Fig. R6 a CLSM image and **b** bright field of $\mathbf{1}_{\mathbf{Mono}}$ in 97:3 TO/1-propanol; λ_{ex} = 405 nm; λ_{em} = 490– 590 nm.

On page 5 of the revised manuscript, 2nd paragraph: *Under these mixing conditions, fluorescence microscopy of the TO/1-propanol mixture did not reveal any micrometer-scale droplet-like phase separation.*

[What has been revised in this manuscript]
Our reply to the Reviewer' s comments and what has been revised are listed as follows.

Response to the comments from Reviewer 2:

Reviewer #2: Naruse, Yamaguchi and co-workers provide an updated and revised version of their manuscript. The authors have expanded their existing experimental dataset primarily with further spectroscopic investigations to underscore their findings on the influence of fatty acid-based solvents on supramolecular polymerizations. In my earlier report, I wrote that in my opinion, the manuscript provides further proof and corroboration for earlier reports, while also providing an opportunity to investigate solvent effects in more detail and obtain new mechanistic understanding.
The manuscript provides a strong collection of experimental findings, but despite their further clarifications on their experimental data, the current manuscript does not shed conceptually new light on the role of solvents in supramolecular polymerizations. In that sense, I agree with reviewer 1, and think that this manuscript is interesting to a selected audience, rather than Nature Communication' s broad public. Hence, I remain with my recommendation to publish the manuscript elsewhere.

Re: We sincerely thank the reviewer for the careful re-evaluation of our revised manuscript and for the thoughtful comments. We appreciate the reviewer's recognition that the expanded spectroscopic investigations further substantiate our conclusions regarding the influence of fatty acid-based solvents on supramolecular polymerization. At the same time, we acknowledge the reviewer's concern regarding the conceptual scope of the present study.

We would like to clarify that the principal new insight of this work lies in demonstrating that a neutral lipid medium can actively influence the kinetic pathway of supramolecular polymerization, rather than simply acting as a passive low-polarity solvent. Building on our previous studies on amino acid-based diamide systems, the present study reveals that triolein delays spontaneous nucleation, suppresses inter-fiber bundling, and thereby enables pathway-selective seeded growth. We believe that this mechanistically informative finding represents the main conceptual advance of the present work, and we have revised the manuscript accordingly.

Below are some further comments for the authors that I hope will be helpful in revising their manuscript:
- New insights that extend beyond what can readily be obtained with the conventional suite of spectroscopies and microscopies would give interesting new insights into novel aspects or generalities of the solvent-mediated supramolecular polymerizations.

Re: We thank the reviewer for this important comment. We agree that broader mechanistic generalization will ultimately require further investigation. We would like to clarify that the principal new insight of the present work lies in the conceptual and mechanistic understanding gained from the current experimental system. Building on our previous studies of amino acid-based diamide systems, initiated by our 2018 report on precision supramolecular polymerization (*Angew. Chem. Int. Ed.* **2018**, *57*, 2339–2343), we have developed a kinetic framework for controlling supramolecular polymerization pathways under metastable, non-equilibrium conditions (*J. Am. Chem. Soc.* **2021**, *143*,

2953–2961; *Angew. Chem. Int. Ed.* **2025**, *64*, e202416361; *Chem. Commun.* **2025**, *61*, 16981–16984). Based on this framework, the present study reveals that triolein is not merely a passive low-polarity medium, but rather a distinctive neutral lipid environment that actively influences the supramolecular polymerization pathway. We consider this to be the key new insight of the present work, and we have revised the manuscript to make this point clearer while avoiding overgeneralization beyond the present system.

On page 3 of the revised manuscript, in the third paragraph of the Introduction: *In our previous studies on amino acid-based diamide systems, we established a kinetic framework for controlling supramolecular polymerization pathways under metastable, non-equilibrium conditions.*[51] *Building on this background, we investigated whether a neutral lipid medium could actively reshape such kinetically controlled assembly pathways beyond what would be expected from bulk polarity alone.*

Reference: *[51] (a) Ogi, S., Matsumoto, K. & Yamaguchi, S. Seeded polymerization through the interplay of folding and aggregation of an amino-acid-based diamide. Angew. Chem. Int. Ed.* ***57****, 2339–2343 (2018); (b) Choi, H., Ogi, S., Ando, N. & Yamaguchi S. Dual Trapping of a Metastable Planarized Triarylborane π-System Based on Folding and Lewis Acid–Base Complexation for Seeded Polymerization. J. Am. Chem. Soc.,* ***143****, 2953–2961 (2021); (c) Matsumoto, K., Bäumer, N., Ogi, S. & Yamaguchi, S. Kinetic Control over Social and Narcissistic Self-Sorting from Multicomponent Mixtures in Seed-Initiated Supramolecular Polymerization by Fine-Tuning of Steric Effects. Angew. Chem. Int. Ed.,* ***64****, e202416361 (2025); (d) Yamada, S., Inai, N., Ogi, S., Yanai, T. & Yamaguchi, S. Seeded control of symmetry-directed diverse chiral π-stacked benzothiadiazole assemblies with leucine-based diamide units. Chem. Commun.,* ***61****, 16981–16984 (2025).*

The IR results point to hydrogen bonding of the solvent with the monomers, which is very interesting. Additional work in this direction could shed light on interesting properties of the system. How are these hydrogen bonds organized? Are they strong, weak, dynamic, rigid? Do MD simulations provide clues? Or time-resolved spectroscopies? Are these principles general, and if so, how can one use them to engineer specific properties of the system in a targeted way?

Re: We thank the reviewer for this insightful comment. We agree that the solvent–monomer hydrogen bonding suggested by the IR measurements is a particularly interesting aspect of the present system. Our current data support the view that these interactions play an important role in stabilizing the monomeric state and in modulating nucleation and bundling behavior. However, we agree that the detailed organization and dynamic nature of these hydrogen bonds cannot be established conclusively from the present dataset alone.

Further studies along this line will be valuable for clarifying these interactions in more detail. In particular, molecular simulations may provide useful insight into the preferred hydrogen-bonding motifs, their dynamics, and their contributions to pathway selection during supramolecular polymerization. As demonstrated in our previous studies, MD simulations can provide mechanistic insight into the dissociation and association behaviors of intermolecular hydrogen bonds at the termini and within the interior of supramolecular polymer chains (*J. Am. Chem. Soc.* **2022**, *144*, 22479–22492). Furthermore, recent report reveals how reversible hydrogen-bond dynamics contribute to the stress-relaxation processes governing the formation and dynamics of cyclic supramolecular polymers (*Angew. Chem. Int. Ed.* **2025**, *64*, e202512811). At the same

time, treating a neutral lipid medium such as triolein, which is substantially larger and structurally more complex than conventional organic solvents, remains computationally demanding. We therefore consider such approaches to be an important direction for future work on the present system. Such investigations will also be important for assessing the broader generality of these principles and for exploring how they may be used to engineer specific properties of related systems.

In response to this comment, we have revised the Discussion to make these points clearer and to better define the scope of the present mechanistic interpretation.

On page 15 of the revised manuscript, in the third paragraph of the Discussion: *Consistent with this proposal, our kinetic studies revealed delayed nucleation, stabilization of the dispersed aggregated state, and exceptional responsiveness to seeded growth (red filled circles in Fig. 3h), demonstrating that these solvent-specific effects enable precise kinetic control over supramolecular polymer growth. The observation of related kinetic features in EO further supports the view that neutral lipids can influence the kinetic pathway of supramolecular polymerization in ways not captured by conventional low-polarity solvents alone. Although the present FT-IR data support the participation of such solvent–monomer hydrogen bonding, the detailed organization and dynamic nature of these interactions remain to be clarified. Molecular dynamics simulations will be valuable for elucidating the preferred hydrogen-bonding motifs, their dynamics, and their roles in nucleation and bundling.*[57,58] *Importantly, we applied these solvent-dependent characteristics to achieve stepwise seeded growth of supramolecular multiblock nanostructures in TO.*

Reference: *[57] Fukaya, N. et al. Impact of hydrophobic/hydrophilic balance on aggregation pathways, morphologies, and excited-state dynamics of amphiphilic diketopyrrolopyrrole dyes in aqueous media. J. Am. Chem. Soc.,* ***144****, 22479–22492 (2022).*
[58] Nodera, Y. et al. The role of hydrogen bonding in the formation and dynamics of two distinct types of cyclic supramolecular polymers. Angew. Chem. Int. Ed. ***64****, e202512811 (2025).*

As for the fitting, the authors could give some further details. The SI of the reference they cite for fitting does actually report normalization of the data. Did the authors do this too, or not? These details remain unclear.

Re: We thank the reviewer for pointing this out. In the Supporting Information of the cited reference (*J. Am. Chem. Soc.* **2012**, *134*, 13482–13491), the denaturation data were analyzed using normalized aggregation curves with normalization factors introduced when necessary. In our analysis, the denaturation curves were imported as raw absorbance data and fitted directly by global fitting using a MATLAB routine. During this fitting procedure, we selected the option "Normalize each curve individually", such that an individual normalization constant was optimized for each curve. We have now clarified this point in the revised manuscript.

On page 18 of the revised manuscript: ***Denaturation curve fitting.*** *Solvent-dependent denaturation curves were analyzed by global fitting with a cooperative supramolecular polymerization equilibrium model based on the nucleation–elongation mechanism, following the approach of Korevaar et al.*[52] *The experimental absorbance data were imported into the MATLAB fitting routine as raw data. Normalization was included during the fitting procedure by selecting the option "Normalize each curve individually". The fitted curves shown in Figs. 2e, S6d, and S7d were used to derive the thermodynamic parameters summarized in Supplementary Table S1.*

Response to the comments from Reviewer 3:

Reviewer #3: The authors have fully responded to all the comments raised by this reviewer, as well as those from the other reviewers, in a detailed and satisfactory manner. In addition, they have carried out a series of new experiments, including nucleation studies in mixed solvents (with varying compositions), temperature-dependent UV measurements, and fluorescence microscopy, all of which have contributed to improving and clarifying the content of the paper. I sincerely appreciate the authors' careful revisions. In my opinion, all the points raised by the reviewers have been clearly addressed, and the revised manuscript is now suitable for publication in Nature Communications.
I would like to congratulate the authors on their conceptually insightful and well-executed work.

Re: We sincerely thank the reviewer for the very positive assessment of our revisions and additional experiments. We are encouraged by the reviewer's kind remarks on the conceptual significance and quality of this work, as well as by the judgment that the revised manuscript is now suitable for publication in *Nature Communications*.
